# Time trends in newly recorded diagnoses of 19 long term conditions before, during, and after the covid-19 pandemic: population based cohort study in England using OpenSAFELY

**DOI:** 10.1136/bmj-2025-086393

**Published:** 2026-01-22

**Authors:** Mark D Russell, Andrea Schaffer, Katie Bechman, Mark Gibson, Jon Massey, Rose Higgins, Brian MacKenna, Peter Inglesby, Seb Bacon, Amir Mehrkar, Ben Goldacre, Edward Alveyn, Victoria Allen, Zijing Yang, Samir Patel, Maryam A Adas, Gurjinder Sandhu, Elizabeth Price, Rouvick M Gama, Kate Bramham, Matthew Hotopf, Sam Norton, Andrew P Cope, James B Galloway

**Affiliations:** 1 Centre for Rheumatic Diseases, King’s College London, SE5 9RJ, UK; 2 Bennett Institute for Applied Data Science, Nuffield Department of Primary Care Health Sciences, University of Oxford, Oxford, UK; 3 King’s College Hospital NHS Foundation Trust, London, UK; 4 Great Western Hospital NHS Foundation Trust, Swindon, UK; 5 Department of Inflammation Biology, King’s College London, London, UK; 6 Centre for Nephrology, Urology, and Transplantation, King’s College London, London, UK; 7 Institute of Psychiatry, Psychology, and Neuroscience, King’s College London, London, UK

## Abstract

**Objective:**

To evaluate temporal changes in rates of newly recorded diagnoses for 19 long term conditions in England in relation to the covid-19 pandemic by disease, age group, sex, socioeconomic status, and ethnicity.

**Design:**

Population based cohort study.

**Setting:**

Primary care and hospital admission data, with the approval of NHS England.

**Participants:**

29 995 025 individuals registered with general practices in England contributing data to the OpenSAFELY-TPP platform.

**Main outcome measures:**

Temporal trends in age and sex standardised incident and prevalent diagnosis rates for 19 long term conditions between 1 April 2016 and 30 November 2024. Differences between expected and observed diagnosis rates after the onset of the covid-19 pandemic were compared using seasonal autoregressive integrated moving-average models, based on modelled projections of expected rates from pre-pandemic patterns.

**Results:**

All 19 conditions showed a sharp decline in newly recorded diagnoses during the first year of the pandemic, followed by variable recovery. As of November 2024, cumulative reductions in diagnoses remained evident for conditions such as depression (734 800 (27.7%) fewer diagnoses than expected; 95% prediction interval (PI) 703 100 to 766 400), asthma (152 900 (16.4%) fewer diagnoses; 95% PI 137 500 to 168 300), chronic obstructive pulmonary disease (COPD) (90 100 (15.8%) fewer diagnoses; 95% PI 81 400 to 98 900), psoriasis (54 700 (17.1%) fewer diagnoses; 95% PI 50 100 to 59 200), and osteoporosis (54 100 (11.5%) fewer diagnoses; 95% PI 47 100 to 61 100). Conversely, diagnoses of chronic kidney disease have increased by 34.8% above expected levels during the pandemic recovery period, corresponding to 359 000 additional diagnoses (95% PI 333 500 to 384 500). Unadjusted subgroup analyses stratified by ethnicity and socioeconomic status indicated that, after an initial decrease, dementia diagnosis rates have risen above pre-pandemic levels for people of white ethnicity and in less deprived socioeconomic areas, but not for those from other ethnicities and more deprived areas.

**Conclusions:**

Since the covid-19 pandemic, there have been fewer diagnoses than expected for conditions such as depression, asthma, COPD, and osteoporosis, in contrast with a rapid increase in diagnoses of chronic kidney disease since 2022. Unadjusted analyses stratified by ethnicity and socioeconomic status suggest differential patterns of recovery, particularly for individuals with dementia. This study highlights the potential for near real time monitoring of disease epidemiology using routinely collected health data, informing strategies to enhance case detection and investigate inequities in healthcare.

## Introduction

The covid-19 pandemic had a major impact on healthcare systems worldwide, disrupting service delivery through redeployment of staff, implementation of stay-at-home guidance, and abrupt shifts in healthcare utilisation.^1^ ^2^ ^3^ ^4^ Studies from earlier in the pandemic highlighted the consequences of these disruptions on care for numerous long term health conditions, with abrupt decreases in newly recorded diagnoses across a wide range of diseases.^5^ ^6^ ^7^ ^8^ ^9^ ^10^ ^11^ ^12^ However, no population level studies have comprehensively evaluated the extent of recovery in recorded diagnosis rates across multiple long term conditions as healthcare services emerge from the pandemic.

The OpenSAFELY platform provides an opportunity to address this knowledge gap. OpenSAFELY enables analysis of primary care and hospital data in a secure environment for up to 58 million people, covering 99% of England’s population.^13^ In proof-of-concept studies, data in OpenSAFELY were used to highlight noticeable decreases in newly recorded diagnoses of autoimmune inflammatory arthritis (eg, rheumatoid arthritis) and gout during the early phase of the pandemic.^6^ ^7^ Although diagnosis rates for these conditions returned to pre-pandemic levels during the second year of the pandemic, no compensatory increase in diagnosis rates above pre-pandemic levels had been observed as of 2023, indicating that a substantial proportion of conditions remained undiagnosed at that point.^6^ ^7^ ^14^

Using population level data in OpenSAFELY, we evaluated changes in diagnosis rates for 19 long term conditions, spanning multiple disease areas, since the covid-19 pandemic, stratified by disease, age group, sex, socioeconomic status, and ethnicity.

## Methods

### Study design and data source

A population level, observational cohort study was performed using linked primary and secondary care data in the OpenSAFELY-TPP platform, which contains health data for people registered with general practices in England using TPP SystmOne software.^13^ OpenSAFELY-TPP coverage varies throughout England, with higher coverage in regions such as East of England and lower coverage in London and South East England.^15^ Previous studies have shown that OpenSAFELY-TPP is representative of the general population of England in terms of age, sex, socioeconomic status, ethnicity, and leading causes of death compared with national estimates from the Office for National Statistics.^15^ Primary care records were linked to the Hospital Episode Statistics dataset through OpenSAFELY.

### Study population and case definitions

The study period was from 1 April 2016 to 30 November 2024. The reference population consisted of all people registered with TPP practices in England at any point during the study period who did not have type 1 opt-outs registered with their practice (type 1 opt-outs indicate those who do not want their personal data being shared with any organisation not involved in their direct care, representing about 3% of the population of England in 2017^16^ ^17^).

Incident and prevalent diagnosis rates were reported for 19 long term conditions: asthma, atopic dermatitis, coronary heart disease, chronic kidney disease stages 3 to 5, coeliac disease, chronic obstructive pulmonary disease (COPD), Crohn’s disease, dementia, depression, type 2 diabetes mellitus, epilepsy, heart failure, multiple sclerosis, osteoporosis, polymyalgia rheumatica, psoriasis, rheumatoid arthritis, stroke/transient ischaemic attack, and ulcerative colitis. These conditions were chosen as examples of long term conditions with well defined diagnostic codes, spanning a range of disease areas, to enable comparisons of the relative impact of the pandemic on different diseases. We defined incident diagnoses as the first appearance of a diagnostic code for each condition in an individual’s primary care or hospital admission records (see supplementary appendix pages 38-43 for code lists). Individuals’ historical primary care records were included to enable retrospective ascertainment and exclusion of prevalent diagnoses before the study period, ensuring that we included only incident diagnoses within the study period. Additionally, people were required to have at least 12 months of continuous general practice registration before their index diagnosis. We included hospital admissions with relevant diagnostic codes listed as the primary cause for that admission. Hospital admissions with diagnostic codes listed in secondary positions were not included, owing to less reliable coding—for example, the inclusion of secondary admission codes for rheumatoid arthritis resulted in incidence rates that were threefold higher than estimates from previous population level studies.^6^ ^18^

We calculated monthly incidence rates of newly recorded diagnoses for each condition by dividing the number of people with index diagnoses in each calendar month, by the number of people in the reference population who were alive and currently registered with TPP general practices in England in that calendar month, without previously recorded diagnostic codes for the condition, who had at least 12 months of continuous practice registration before the start of each month. Those who later deregistered or died had their data censored in subsequent months. Direct age and sex standardisation was performed with reference to the 2013 European standard population.^19^ For computational reasons, we selected 10 year age bands over five year age bands up to age 80 years. For comparison, age and sex standardised diagnosis rates are presented alongside rates without standardisation. Monthly diagnosis rates are presented per 100 000 population alongside rolling averages over three months, representing the mean of the current, previous, and subsequent months. We report diagnosis rates for each condition separately by sex, age band, socioeconomic status (defined as fifths of index of multiple deprivation,^20^ from the lowest fifth (most deprived) to highest fifth (least deprived), or unknown), and ethnicity (categorised into white, mixed, Asian or Asian British, black or black British, Chinese or other ethnic groups, or unknown, as recorded in primary or secondary care records^21^). No age or sex standardisation was performed for subgroup analyses by index of multiple deprivation or ethnicity for computational reasons. Conditions with small numbers of new diagnoses when analysed separately by ethnicity are not shown (coeliac disease, Crohn’s disease, epilepsy, multiple sclerosis, osteoporosis, polymyalgia rheumatica, rheumatoid arthritis, and ulcerative colitis) owing to the potential for disclosure.

We estimated the temporal trends in the number of recorded prevalent diagnoses for each condition by dividing the number of people with prevalent diagnostic codes on April 1 of each year of the study period by the number of those who were alive and currently registered at the same time point. Yearly age and sex standardised estimates of prevalent diagnoses per 100 000 population are presented in addition to estimates without standardisation.

A sensitivity analysis was performed to explore potential reasons for the observed decline in diagnoses of depression during the study period. We used a combined code list that included codes indicative of depressive symptoms (eg, depressed mood) and confirmed depression diagnostic codes (see supplementary appendix page 40 for code lists). To prevent double counting, we assigned a single index date to each individual corresponding to the first occurrence of any code from this combined code list.

### Statistical analysis

Baseline sociodemographic characteristics were tabulated both without inferential statistics for the reference population and separately for those with newly recorded diagnoses of each condition during the study period.

We estimated the differences between expected and observed diagnosis rates after the onset of the covid-19 pandemic for each condition using seasonal autoregressive integrated moving average (SARIMA) models—a forecasting method that accounts for underlying trends and seasonal patterns in time series data (see supplementary appendix pages 36-37 for further details).^5^ ^22^ Monthly diagnosis rates from April 2016 to February 2020 (ie, before the onset of the first covid-19 lockdown in England) were used to model expected diagnosis rates from March 2020 to November 2024, based on the assumption of stable pre-pandemic trends. Absolute and relative differences between expected and observed diagnosis rates were calculated and are presented for the full post-pandemic period (March 2020 to November 2024) and separately for four periods: March 2020 to February 2021, March 2021 to February 2022, March 2022 to February 2023, and March 2023 to November 2024. We estimated absolute differences in numbers of newly recorded diagnoses for the full population of England by extrapolating differences in expected versus observed diagnosis rates for the study population to mid-year population estimates for England (rounded to the nearest 100).^23^ All estimates from forecasted models are reported with 95% prediction intervals (PIs), which reflect the increasing uncertainty as projections extend further from the observed data.

Model selection was verified using residual diagnostics, comprising plots of residuals over time, residual histograms, residual autocorrelation, and partial autocorrelation plots, in addition to Ljung-Box tests.^24^ Bai-Perron tests^25^ were used to detect structural breaks in pre-pandemic time series, with break selection based on the bayesian information criterion, in addition to cumulative sum tests to assess the stability of variables over time. To assess the robustness of SARIMA model specification, we conducted sensitivity analyses using Prophet forecasting methodology—a decomposable time series model with a piecewise linear trend and Fourier seasonality.^26^ The supplementary appendix (pages 36-37) provides further details of sensitivity analyses and assumption tests.

Python 3.11 was used for data management and Stata version 18 and R version 4.41 for statistical analyses. Code for data management and analysis, as well as code lists, are available online (https://github.com/opensafely/disease_incidence). As analyses were primarily descriptive, no correction for multiple hypothesis testing was performed. For statistical disclosure control, frequency counts were rounded to the nearest 5 and non-zero counts <8 were redacted.

### Patient and public involvement

As this study was a secondary analysis of routinely collected, anonymised health data, no patients or members of the public were involved in the design or conduct of these analyses. More broadly, OpenSAFELY has involved patients and the public in various ways, including a public website that provides a detailed description of the platform in language suitable for a lay audience (https://opensafely.org), participation in two citizen juries exploring public trust in OpenSAFELY, co-development of an explainer video (https://www.opensafely.org/about/), patient representation on our OpenSAFELY oversight board, partnering with Understanding Patient Data to produce plain language explanations on the importance of large datasets for research, and presentations at various online public engagement events to key communities. To ensure that patients’ voices are represented, OpenSAFELY are working closely to decide on language choices with appropriate medical research charities (eg, Association of Medical Research Charities).

## Results

Overall, 29 995 025 people in England had data available for analysis in OpenSAFELY-TPP between 1 April 2016 and 30 November 2024. Supplementary appendix table S1 shows the sociodemographic data for these individuals, and for those with newly recorded diagnoses of 19 long term conditions.

**Fig 1:**
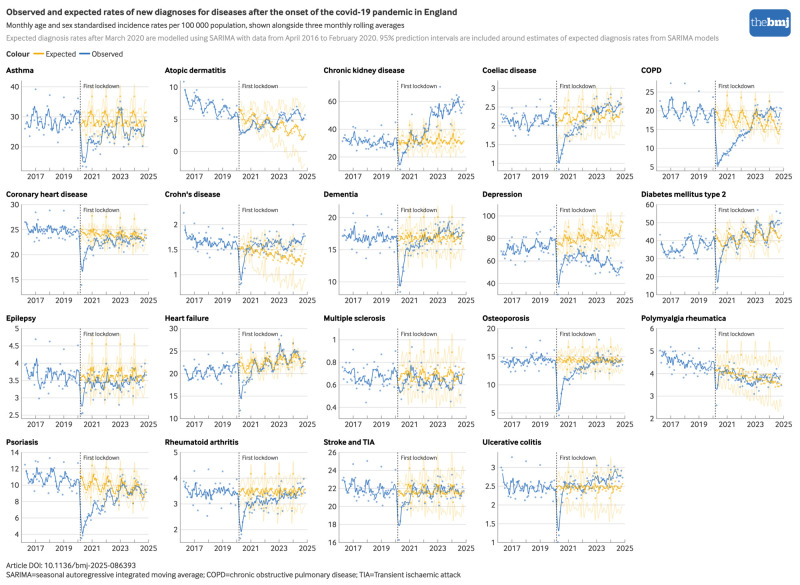
Comparison between observed and expected rates of newly recorded diagnoses for 19 long term conditions in England after the onset of the covid-19 pandemic. Supplementary figure S8 includes sensitivity analyses utilising Prophet forecasting methodology. An interactive version of this graphic is available at https://public.flourish.studio/visualisation/25824634/

Relative to pre-pandemic trends, rates of new diagnoses for all 19 conditions decreased sharply after the onset of the covid-19 pandemic; however, the magnitude of these decreases varied substantially by condition (see supplementary appendix figures S1 and S2). Figure 1, table 1, and supplementary appendix table S2 show the observed compared with expected diagnosis rates after March 2020 (modelled using SARIMA). During the first year of the pandemic, the largest relative deficits in diagnosis rates were observed for COPD (−55.7%, 95% PI −56.8% to −54.6%), psoriasis (−43.6%, −44.9% to −42.1%), atopic dermatitis (−38.6%, −42.4% to −34.3%), osteoporosis (−35.3%, −36.9% to −33.6%), coeliac disease (−32.9%, −36.0% to −29.6%), and asthma (−31.5%, −33.5% to −29.4%). The smallest relative deficits in diagnosis rates were observed for stroke/transient ischaemic attack (−9.3%, −10.8% to −7.7%) and polymyalgia rheumatica (−7.4%, −10.7% to −3.9%).

**Table 1:** Differences between expected and observed rates of newly recorded diagnoses for 19 long term conditions in England after the onset of the covid-19 pandemic. Diagnosis rates are per 100 000 population

Condition	First year of pandemic (March 2020-February 2021)				Cumulative difference for period after onset of pandemic (March 2020-November 2024)			
	Observed diagnosis rate	Expected diagnosis rate (95% PI)	% difference between expected and observed (95% PI)		Observed diagnosis rate	Expected diagnosis rate (95% PI)	% difference between expected and observed (95% PI)	Absolute difference in diagnoses extrapolated to population of England (95% PI)
Asthma	237	346 (336 to 356)	−31.5 (−33.5 to −29.4)		1370	1638 (1611 to 1665)	−16.4 (−17.7 to −15.0)	−152 900 (−168 300 to −137 500)
Atopic dermatitis	40.0	65.1 (60.8 to 69.4)	−38.6 (−42.4 to −34.3)		261	242 (221 to 263)	7.6 (−1.0 to 17.8)	10 500 (−1510 to 22 500)
Coronary heart disease	239	293 (289 to 297)	−18.4 (−19.6 to −17.3)		1269	1369 (1358 to 1379)	−7.3 (−8.0 to −6.6)	−56 900 (−63 000 to −50 800)
Chronic kidney disease	283	379 (364 to 394)	−25.3 (−28.2 to −22.2)		2440	1811 (1766 to 1856)	34.8 (31.5 to 38.2)	359 000 (333 500 to 384 500)
Coeliac disease	17.8	26.6 (25.3 to 27.8)	−32.9 (−36.0 to −29.6)		121	129 (126 to 132)	−6.4 (−8.4 to −4.2)	−4670 (−6290 to −3040)
COPD	98.8	223 (217 to 229)	−55.7 (−56.8 to −54.6)		841	999 (983 to 1014)	−15.8 (−17.1 to −14.5)	−90 100 (−98 900 to −81 400)
Crohn’s disease	15.7	18.3 (17.8 to 18.9)	−14.2 (−16.8 to −11.3)		89.1	80.8 (78.4 to 83.2)	10.3 (7.1 to 13.6)	4720 (3350 to 6090)
Dementia	156	202 (196 to 208)	−22.8 (−25.0 to −20.5)		926	962 (947 to 977)	−3.8 (−5.2 to −2.3)	−20 600 (−29 000 to −12 200)
Depression	657	931 (909 to 953)	−29.4 (−31.0 to −27.7)		3364	4652 (4596 to 4707)	−27.7 (−28.5 to −26.8)	−734 800 (−766 400 to −703 100)
Diabetes mellitus type 2	367	479 (460 to 497)	−23.3 (−26.1 to −20.2)		2372	2386 (2344 to 2429)	−0.6 (−2.4 to 1.2)	−8400 (−32 600 to 15 800)
Epilepsy	38.8	43.9 (42.8 to 45.1)	−11.8 (−14.0 to −9.3)		195	208 (205 to 211)	−6.3 (−7.4 to −5.0)	−7420 (−8930 to −5900)
Heart failure	211	262 (257 to 266)	−19.5 (−21.0 to −18.0)		1244	1283 (1272 to 1294)	−3.0 (−3.9 to −2.2)	−22 100 (−28 400 to −15 900)
Multiple sclerosis	7.3	8.3 (7.8 to 8.7)	−11.4 (−15.7 to −6.5)		36.2	39.0 (37.9 to 40.2)	−7.1 (−9.8 to −4.3)	−1580 (−2240 to −930)
Osteoporosis	112	173 (169 to 177)	−35.3 (−36.9 to −33.6)		727	822 (810 to 834)	−11.5 (−12.8 to −10.2)	−54 100 (−61 100 to −47 100)
Polymyalgia rheumatica	46.5	50.2 (48.4 to 52.0)	−7.4 (−10.7 to −3.9)		217	224 (218 to 230)	−3.1 (−5.6 to −0.6)	−4010 (−7320 to −710)
Psoriasis	69.6	123 (120 to 127)	−43.6 (−44.9 to −42.1)		465	561 (553 to 569)	−17.1 (−18.2 to −15.9)	−54 700 (−59 200 to −50 100)
Rheumatoid arthritis	31.5	41.7 (40.4 to 42.9)	−24.3 (−26.6 to −21.9)		179	198 (194 to 202)	−9.8 (−11.4 to −8.0)	−11 000 (−13 200 to −8880)
Stroke/TIA	237	261 (257 to 265)	−9.3 (−10.8 to −7.7)		1215	1239 (1226 to 1252)	−1.9 (−2.9 to −0.9)	−13 600 (−20 900 to −6390)
Ulcerative colitis	25.6	29.6 (28.4 to 30.8)	−13.5 (−16.8 to −9.8)		144	141 (137 to 144)	2.4 (0.1 to 5.0)	1960 (46 to 3880)

Expected and observed diagnosis rates for each condition are compared during the first year after the onset of the pandemic (1 March 2020 to 28 February 2021) and during the full study period after the onset of the covid-19 pandemic (1 March 2020 to 30 November 2024). Expected diagnosis rates were modelled using seasonal autoregressive integrated moving averages (SARIMA), utilising data from April 2016 to February 2020 under the assumption that pre-pandemic trends would have continued unchanged if the pandemic had not occurred. The relative differences between the number of expected versus observed diagnoses are shown, in addition to absolute differences in expected versus observed diagnoses extrapolated to the full population of England (rounded to the nearest 10 for counts between 1000 and 10 000; and to the nearest 100 for counts above 10 000).

COPD=chronic obstructive pulmonary disease; PI=prediction interval; TIA=transient ischaemic attack.

By November 2024, fewer diagnoses remained than expected for several conditions relative to pre-pandemic trends, with depression showing the largest cumulative reduction between March 2020 and November 2024 (−27.7%, 95% PI −28.5% to −26.8%). After a decrease in new diagnoses of depression during the first year of the pandemic (29.4%, 27.7% to 31.0%), rates increased but remained below pre-pandemic levels, followed by a further decline between 2022 and 2024 (fig 1). When extrapolated to the full population of England, an estimated 27.7% fewer diagnoses of depression (734 800, 95% PI 703 100 to 766 400) than expected occurred between March 2020 and November 2024. In a sensitivity analysis, when codes indicative of depressive symptoms (eg, depressed mood) were included from an expanded depression code list (see supplementary figure S3), the relative deficit in depression diagnoses was attenuated (−20.9%, 95% PI −22.0% to −19.9%); however, the absolute reduction in diagnoses was comparable (738 200 fewer diagnoses, 95% PI 690 700 to 785 700), indicative of the higher diagnosis rate for depression when including these additional codes.

The next largest absolute reductions in diagnoses were seen for asthma (152 900 (16.4%) decrease, 95% PI 137 500 to 168 300), COPD (90 100 (15.8%), 81 400 to 98 900), coronary heart disease (56 900 (7.3%), 50 800 to 63 000), psoriasis (54 700 (17.1%), 50 100 to 59 200), and osteoporosis (54 100 (11.5%), 47 100 to 61 100) (table 1). In contrast, diagnosis rates for other conditions increased above pre-pandemic levels after the initial decreases—most notably for chronic kidney disease (table 1). After a 25.3% decrease (95% PI 22.2 to 28.2) in new diagnoses of chronic kidney disease during the first year of the pandemic, diagnosis rates returned to pre-pandemic levels by late 2020, before increasing above pre-pandemic levels from 2022 onwards (fig 1). Between March 2020 and November 2024, the number of newly recorded diagnoses of chronic kidney disease was 34.8% higher than expected based on pre-pandemic trends (95% PI 31.5 to 38.2), corresponding to an estimated 359 000 additional diagnoses for this disease in England (95% PI 333 500 to 384 500).

**Fig 2:**
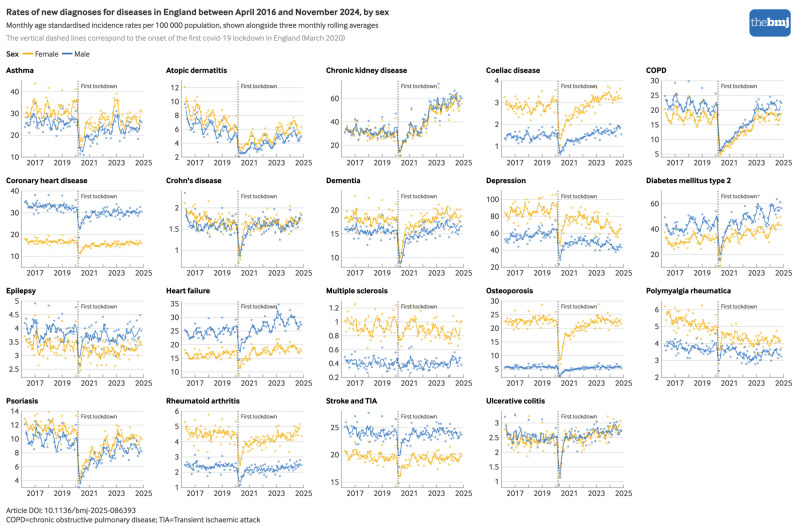
Monthly rates of newly recorded diagnoses per 100 000 population for 19 long term conditions in England between 1 April 2016 and 30 November 2024 by sex. An interactive version of this graphic is available at https://public.flourish.studio/visualisation/25257909/

**Fig 3:**
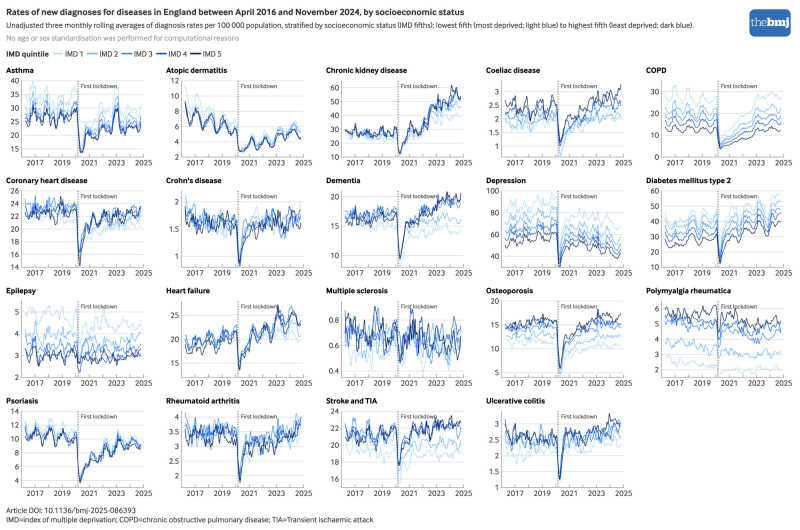
Monthly rates of newly recorded diagnoses per 100 000 population for 19 long term conditions in England between 1 April 2016 and 30 November 2024 by socioeconomic status. An interactive version of this graphic is available at https://public.flourish.studio/visualisation/25824451/

When analysed separately by sex (fig 2), age group (see supplementary figure S4), socioeconomic status (fig 3), and ethnicity (see supplementary figure S5), several inequities were evident in the recovery of diagnosis rates after the initial pandemic phase. For chronic kidney disease, much of the increase in diagnoses during the pandemic recovery period occurred in people aged 80 years and older, and in those from less deprived socioeconomic fifths, with comparable relative increases by ethnicity. For depression, the decrease in diagnoses during the pandemic recovery period occurred predominantly in those aged 20 to 39 years and in those of white or mixed ethnicity, with comparable decreases across socioeconomic fifths. For dementia, after the initial decrease in diagnoses, rates increased above pre-pandemic levels for people of white ethnicity and those from less deprived socioeconomic areas, but not for those of other ethnicities or from more deprived socioeconomic areas. Although for computational reasons, age and sex standardisation could not be performed in subgroup analyses by ethnicity or socioeconomic status, crude and age and sex adjusted estimates were comparable overall (see supplementary figure S2).

Changes were notable in the number of prevalent diagnoses for several conditions after the onset of the pandemic compared with pre-pandemic trends (see supplementary figures S6 and S7). The gradually declining prevalence of dementia before the pandemic then accelerated during the first year of the pandemic, before stabilising from 2021 onwards. Although the prevalence of COPD had remained stable before the pandemic, it decreased after 2020, likely corresponding to the observed decrease in newly recorded diagnoses. In contrast, the declining pre-pandemic prevalence of chronic kidney disease began increasing after 2022, corresponding to the noticeable increase in new diagnoses at that point.

Sensitivity analyses were conducted using Prophet forecasting methodology to evaluate the robustness of estimates from SARIMA models, with forecasted estimates comparable for many diseases, including chronic kidney disease and depression (see supplementary figure S8 and table S3). Estimates from Prophet models compared with SARIMA showed more pronounced reductions in recorded diagnoses for asthma, coeliac disease, COPD, type 2 diabetes mellitus, heart failure, and stroke/transient ischaemic attack, whereas estimates from SARIMA models showed relatively larger increases for Crohn’s disease. Residual diagnostics from SARIMA models supported the robustness of the overall findings (see supplementary figures S9 to S27). The findings of Ljung-Box tests were non-significant for all models, as were cumulative sum tests of residual instability, with no structural breaks seen on Bai-Perron tests of pre-pandemic time series. Small, isolated residual spikes were evident in autocorrelation plots for type 2 diabetes mellitus, osteoporosis, and psoriasis, which persisted after exploration of alternative SARIMA terms; however, these were not statistically significant on Ljung-Box testing, and counterfactuals were closely comparable to those obtained from Prophet forecasts (see supplementary figure S8), supporting the adequacy of the model fit.

## Discussion

Utilising data on more than 29 million individuals in England, our study highlighted noticeable variation in the impact of the covid-19 pandemic on diagnosis rates across a range of diseases and sociodemographic characteristics. New diagnoses of all 19 long term conditions studied decreased sharply during the first year of the pandemic, but the effect was disproportionate for conditions such as COPD, asthma, psoriasis, and osteoporosis. Increases in diagnoses were subsequently observed for several conditions, particularly chronic kidney disease, for which reported diagnoses have increased substantially since 2022. In contrast, cumulative reductions in diagnoses remain evident nearly five years after the onset of the pandemic, most notably for depression. Unadjusted subgroup analyses stratified by ethnicity and socioeconomic status indicated that after an initial decrease, diagnosis rates for dementia increased above pre-pandemic levels for people of white ethnicity and those from less deprived socioeconomic areas, but not for those of other ethnicities or from more deprived areas. Importantly, this study showed the potential for routinely collected health data to enable monitoring of disease epidemiology in near real time.

### What this study adds in comparison with other studies

In our study, sharp decreases in new diagnoses were evident for all 19 long term conditions at the onset of the pandemic, albeit to varying extents. Numerous factors are likely to contribute to these decreases, including fewer people presenting at a time when primary and secondary care services were under enormous pressure,^1^ ^27^ ^28^ stay-at-home guidance,^29^ and reduced access to routine testing and secondary care referrals.^3^ We observed a disproportionate impact during the early phase of the pandemic on diagnosis rates for conditions that are more reliant on elective diagnostic testing, such as COPD and asthma (spirometry and lung function testing), coeliac disease (endoscopy), and osteoporosis (bone densitometry), or less urgent secondary care referral (such as psoriasis). This finding contrasts with smaller decreases for conditions that are more likely to be diagnosed acutely, such as stroke/transient ischaemic attack, epilepsy, and multiple sclerosis.

Our finding of a disproportionate impact during the early phase of the pandemic on respiratory conditions such as COPD and asthma supports the findings from a previous population level study in Wales, which included data up to December 2021.^5^ Using contemporaneous data in OpenSAFELY, our study found that, although new diagnoses of COPD and asthma gradually returned to pre-pandemic levels after these initial decreases, large cumulative reductions in diagnoses were still evident as of November 2024, potentially indicating that these conditions remained undiagnosed in many individuals. An important contributing factor is likely to be the sudden disruption to spirometry and lung function testing during the pandemic, resulting in large backlogs of patients waiting for tests.^30^ Equitable access to spirometry has been identified as a priority area for the NHS as it recovers from the pandemic, given the impact of delayed diagnosis on the initiation of treatments such as inhaled therapies and pulmonary rehabilitation, as well as preventive measures such as vaccination.^30^

A large reduction in diagnoses was also observed for osteoporosis. Delays in diagnosis and the initiation of bone protection treatment can substantially affect the prognosis for people with osteoporosis. Osteoporotic fractures are associated with a high burden of disability and functional impairment,^31^ with estimates of mortality in the first year after hip fracture ranging from 15% to 36%.^32^ Bisphosphonates reduce the risk of hip and vertebral fractures in postmenopausal women by 35% and 54%, respectively,^33^ highlighting the importance of early diagnosis and treatment. Our findings therefore suggest that a national case finding strategy may be warranted to identify people who have not yet had a diagnosis of or been treated for osteoporosis because of the pandemic.

Of all conditions studied, the largest cumulative reduction in diagnoses was observed for depression. After an initial decrease in diagnoses during the early phase of the pandemic, diagnosis rates for depression approached pre-pandemic levels by late 2021 but have declined markedly since 2022, predominantly in those aged 20 to 39 years and in those of white or mixed ethnicity. Although previous studies have reported large reductions in newly recorded mental health diagnoses during the early phase of the pandemic,^5^ ^8^ the recent decrease in new diagnoses of depression warrants further investigation. Our findings are supported by decreases in newly initiated prescriptions of antidepressant drugs and referrals to mental health services for depression in England since 2022, after previous increases.^34^ ^35^ Importantly, however, the observed decline in newly recorded diagnoses may not necessarily equate to a decrease in the underlying incidence of depression. The number of new claimants of disability related benefits for mental health conditions increased by 190% in England between 2019/20 and 2023/24.^36^ One possible explanation for this divergence is that it is taking longer to formally diagnose depression in people with symptoms owing to increasing pressures on the NHS.^37^ ^38^ Primary care services have faced particular challenges from increased demand since the pandemic. Although the total number of face-to-face consultations has increased, so has the proportion of remote consultations, which together with time pressures in consultations, may make it more challenging to identify non-verbal cues associated with depression.^39^ It is also possible that individuals with symptoms of depression may be accessing services without their diagnoses being recorded in primary or secondary care records. After a national drive to expand access to psychological therapies, referrals to NHS Talking Therapies services increased by nearly two thirds between 2013/14 and 2023/24 (from 1.1 million to 1.8 million referrals, respectively), with self-referrals (ie, without necessarily needing to consult a healthcare professional before referral) constituting 69% of all referrals.^40^ Recommendations from the National Institute for Health and Care Excellence (NICE), updated in June 2022, emphasised the importance of therapy based treatments over antidepressants for new diagnoses of less severe depression.^41^ This, in turn, may have contributed to a change in how clinicians record depression diagnoses. In our sensitivity analysis that included additional diagnostic codes for symptoms of depression (eg, depressed mood), the relative deficit in depression diagnoses was attenuated, indicating a shift towards recording symptom related codes over formal depression diagnoses. Previous studies reported similar findings after the introduction of the Quality and Outcomes Framework (QOF) incentivisation scheme in the NHS, which resulted in general practices switching from recording diagnostic codes for depression to symptom related codes.^42^

In contrast, we observed a large increase in newly recorded diagnoses of chronic kidney disease since 2022. Although this may partially represent a catch-up in diagnoses after a disruption to services during the pandemic, the observed increase in chronic kidney disease diagnoses was 35% higher than expected based on pre-pandemic trends. Subgroup analyses highlighted that much of this increase occurred in people aged 80 years and older, and in those from less deprived socioeconomic areas. Numerous factors could have contributed to this increase, and more work is needed to elucidate the reason. Potential explanations could include an accelerated decline in glomerular filtration rate after covid-19 infection,^43^ ^44^ the inclusion of chronic kidney disease as a risk factor in national cardiovascular prevention audits (eg, CVDPREVENT),^45^ initiatives to facilitate the use of novel treatments to prevent the progression of chronic kidney disease (eg, sodium-glucose cotransporter-2 inhibitors),^46^ and changes in coding practices or frequency of laboratory testing (eg, remote albuminuria testing programmes).^47^ In August 2021, NICE published updated guidance on the assessment and management of chronic kidney disease.^48^ These recommendations included screening of individuals at risk, which may have led to enhanced detection after implementation, without necessarily representing true disease progression attributable to the pandemic. Additionally, updated NICE guidelines recommended removal of ethnicity related adjustment factors when estimating glomerular filtration rates. Use of ethnicity based estimated rates was shown to overestimate glomerular filtration rate and under-recognise chronic kidney disease in people of black ethnicity in the UK,^48^ ^49^ which may have contributed to the observed increase in people of black ethnicity meeting thresholds for chronic kidney disease after removal of ethnicity based rates. However, the comparable relative increase in chronic kidney disease diagnoses among those of white ethnicity suggests that this factor alone is unlikely to explain the large overall increase in chronic kidney disease diagnoses.

### Implications for clinicians and policymakers

The methodology utilised in this study provides a mechanism through which temporal changes in diagnosis rates can be monitored and variation by age, sex, ethnicity, socioeconomic status, and region highlighted. Despite England having a universal healthcare system, rates of dementia diagnoses in unadjusted subgroup analyses have risen above pre-pandemic levels for individuals of white ethnicity and those from less deprived socioeconomic areas, whereas diagnosis rates remained lower than expected among those from other ethnic backgrounds and more deprived areas. Our findings have important implications for policymakers and clinicians. The results suggest that nearly five years after the onset of the pandemic large cumulative reductions in diagnoses remain evident for several long term conditions, relative to pre-pandemic trends. Strategies are urgently needed to investigate the reasons for these reductions and decrease the impact of undiagnosed disease and unmet need on patients, the healthcare system, and the economy. Strategies could include national and community based initiatives for case finding, outreach programmes, and screening of people at risk. Importantly, by being able to quantify the relative burden of underdiagnosis by condition, demography, and region, the methodology presented in this study could inform the development of these strategies and help direct the allocation of resources to where they are most needed.

The frequently updated data sources in OpenSAFELY also provide opportunities for early detection of trends in diagnosis, which could be integrated into public health surveillance programmes, such as in response to future pandemics. The ability to detect changes in diagnosis activity at an earlier stage will enable strategies to be implemented proactively to mitigate disruption. Rather than static reports, data in OpenSAFELY can be regularly updated and made publicly available through dashboards at local and national levels.^50^ Additionally, similar methodology can be used to monitor the real world effect of interventions (eg, case finding strategies), while minimising the burden of manual data collection on clinicians and maximising the inclusion of populations who are often under-represented in studies.^51^ ^52^ ^53^ Although the primary focus of this study was to describe changes in healthcare diagnosis activity for long term conditions, the analysis code can be readily adapted to assess care quality benchmarked against clinical guidelines^6^ ^7^ and to evaluate the real world impact of strategies aimed at improving healthcare delivery.^51^

### Strengths and limitations of this study

Our study had several strengths. We used nationally representative data from more than 29 million people to provide updated estimates of incident and prevalent diagnosis rates for 19 long term conditions spanning multiple disease areas. This approach can be expanded to assess numerous other diseases, including rare diseases with limited epidemiological data. While several previous studies had reported abrupt decreases in new diagnoses during the early pandemic,^5^ ^6^ ^7^ ^8^ ^9^ ^10^ ^11^ ^12^ we were able to evaluate whether diagnosis rates had returned to pre-pandemic trends across multiple disease areas as healthcare services emerge from the pandemic. Demonstrating this at a population level requires regularly updated data from primary and secondary care, which is now possible with the introduction of the OpenSAFELY Trusted Research Environment in England.^13^ By showing the feasibility of this approach using routinely collected data from the NHS in England, this study serves as proof-of-principle for data-enabled monitoring of disease epidemiology in other healthcare systems worldwide. Moreover, by collating data sources from different countries, a similar approach would enable comparisons across healthcare systems—as shown in a recent Global Burden of Disease study, which investigated causes of increased disease burden during the early pandemic.^54^

Limitations of this study must also be acknowledged. As with other studies utilising coded health data, the potential for diagnostic misclassification and incomplete or delayed recording of diagnoses must be considered; noting also that the recording of a new diagnostic code does not necessarily equate to the biological onset of a disease—for example, the symptoms and signs of a condition may be monitored or managed for a variable period of time before a formal diagnostic code is recorded. Importantly, although these data can be used to highlight temporal trends in healthcare diagnosis activity, we were unable to infer whether differences represented true changes in underlying disease incidence (eg, after SARS-CoV-2 infection), changes in the recording of diagnoses (eg, as a result of updated guidelines), changes in diagnostic testing (eg, increased testing of B-type natriuretic peptide for heart failure), or changes in service delivery (eg, care provision outside of primary and secondary care). As such, while the acute changes in diagnosis rates were likely initiated by pandemic related disruption, numerous factors beyond the direct consequences of the pandemic could have influenced diagnosis rates in subsequent years, which require further investigation. Similarly, estimates of prevalence from diagnostic coding can be influenced by factors such as coding practices, delays in diagnosis, and mortality, and therefore they may not perfectly match the underlying prevalence of a disease.

Observed trends in diagnosis rates must be considered against the backdrop of abrupt changes in healthcare utilisation and diagnostic testing during the pandemic. The number of general practice appointments in England decreased by one third between March and April 2020, before increasing above pre-pandemic levels.^27^ Pathology testing in primary care decreased by more than 70% during the first month of the pandemic for many common tests, including renal function and glycated haemoglobin, with modestly increased testing above pre-pandemic levels from 2022 onwards.^55^ ^56^ Hospital admissions for non-covid-19 diseases decreased by 34% in 2020, with scheduled and unscheduled admissions decreasing by 47% and 14%, respectively.^57^ To maximise the capture of diagnoses, we utilised data from both primary care and secondary care sources; however, it is important to acknowledge the differences between diagnoses in primary care and those in hospital. We excluded hospital admissions with diagnostic codes recorded in non-primary positions on the basis of reduced coding reliability; although this exclusion could underestimate diagnostic activity if relevant people with a diagnosis were not captured, the concurrent inclusion of primary care records is likely to mitigate this potential bias. Additionally, the forecasting methods in our analyses relied on an assumption of stable pre-pandemic trends that continued after the onset of the pandemic, but this may not be the case for several reasons—for example, changes in national guidance or screening programmes, which can influence diagnosis rates irrespective of the pandemic. As such, the differences between observed and expected diagnosis rates should be viewed as projections based on stable pre-pandemic trends, rather than rates that account for all external factors in the absence of the pandemic. Moreover, the level of uncertainty surrounding projected estimates increases with elapsed time, particularly for less common diseases—although comparable estimates from sensitivity analyses using Prophet supported the robustness of the overall findings.

We were unable to age and sex standardise our subgroup analyses by socioeconomic status or ethnicity owing to the computationally intensive nature of these analyses. As such, these subgroup analyses should be interpreted with caution (eg, if substantial changes in population structure occurred during the study period), although crude and adjusted estimates for the overall cohort were comparable, suggesting this is unlikely to have meaningfully altered our findings. Ten year age bands were utilised for computational reasons and to minimise the risk of disclosivity; however, this may mask heterogeneity within age strata, particularly among the oldest age groups, which could be explored in future analyses. Similarly, we reported trends in diagnosis rates separated into five ethnicity categories for most conditions, owing to small numbers of diagnoses and the potential for disclosure, which precluded more granular reporting of differences within these broad ethnicity categories. Although previous studies have shown OpenSAFELY-TPP to be representative (within 1 percentage point) of the general population of England in terms of demographic characteristics and leading causes of death, primary care coverage by region varies within OpenSAFELY-TPP, which could influence the generalisability of our findings.^15^ Moreover, as this study was performed within the NHS in England, these findings require further investigation in other healthcare systems to assess generalisability globally, including the other UK nations.

It is also important to recognise that methodological differences can influence estimates of absolute incidence rates across studies. For example, incidence rates for depression in our study were lower than those reported in QOF practice disease registers.^58^ A known limitation of QOF registers is that prevalent diagnoses of depression may be recorded as new diagnoses if, for example, a different depression code is entered during a subsequent financial year.^59^ In contrast, our study incorporated a historical look-back to exclude prevalent diagnoses, and individuals were required to have a minimum of 12 months of continuous practice registration before their index diagnosis. Consequently, while absolute incidence estimates may vary between studies owing to differences in methodology or code lists, our approach enables robust comparisons of relative trends in diagnosis rates over time.

### Conclusion

Routinely collected health data on more than 29 million people highlighted large reductions in recorded diagnoses for conditions such as depression, COPD, asthma, and osteoporosis, contrasting with a surge in recorded diagnoses for chronic kidney disease since the covid-19 pandemic. Importantly, this study showed the potential for real time health data to transform disease monitoring, identify inequities across under-served populations, and inform strategies to improve detection of conditions and reduce delays in diagnosis.

**What is already known on this topic**

Early in the covid-19 pandemic, large reductions in newly recorded diagnoses were reported for a range of long term conditions, including chronic obstructive pulmonary disease (COPD), depression, asthma, and rheumatoid arthritis
However, no population level studies have evaluated whether diagnosis rates have subsequently recovered as healthcare services emerge from the pandemic, or compared the impact by disease, age group, sex, socioeconomic status, and ethnicity

**What this study adds**

Large reductions in diagnoses remain evident for conditions such as depression, COPD, asthma, osteoporosis, and psoriasis, in contrast with a large increase in newly recorded diagnoses of chronic kidney disease since the covid-19 pandemic
In unadjusted subgroup analyses, diagnosis rates for dementia have increased above pre-pandemic levels for people of white ethnicity and those from less deprived socioeconomic areas, but not for those from other ethnicities or more deprived areas
This study shows the potential for routinely collected health data to transform monitoring of disease epidemiology in near real time and highlight healthcare inequity

## Data Availability

The code used to analyse the data in this paper is in the supplementary files, and all code is shared openly for review and reuse under MIT open license from https://github.com/opensafely/disease_incidence. All data were linked, stored, and analysed securely using the OpenSAFELY platform (https://www.opensafely.org/) as part of the NHS England OpenSAFELY COVID-19 service. Data include pseudonymised data such as coded diagnoses, drugs, and physiological variables. No free text data are included. No general practice data from patients who have registered a type-1 opt-out with their surgery were included in this study. Detailed pseudonymised patient data are potentially re-identifiable and therefore not shared.

## Web extra

Extra material supplied by authors

Supplementary information: Additional figures S1-S27, tables S1-S3, methods, data, and references

## Notes

Contributors: MDR and JBG conceived the study. JBG, MDR, AS, JM, RH, BMK, PI, SB, AM, and BG curated the data and contributed to software development. MDR led the analysis of the data, supported by JBG and SN. MDR, JBG, AS, SN, KB, MG, ZJ, BMK, RH, and JM provided methodological input. All authors were involved in interpreting the data. MDR drafted the manuscript. All authors reviewed and approved the final manuscript. MDR and JBG are the guarantors and directly verified the underlying data reported in the manuscript. The corresponding author attests that all listed authors meet authorship criteria and that no others meeting the criteria have been omitted.

Access to the underlying identifiable and potentially re-identifiable pseudonymised electronic health record data are tightly governed by various legislative and regulatory frameworks and restricted by best practice. The data in the NHS England OpenSAFELY COVID-19 service are drawn from general practice data across England where TPP is the data processor. TPP developers initiate an automated process to create pseudonymised records in the core OpenSAFELY database, which are copies of key structured data tables in the identifiable records. These pseudonymised records are linked onto key external data resources that have also been pseudonymised through SHA-512 one-way hashing of NHS numbers using a shared salt. University of Oxford, Bennett Institute for Applied Data Science developers and principal investigators, who hold contracts with NHS England, have access to the OpenSAFELY pseudonymised data tables to develop the OpenSAFELY tools. These tools in turn enable researchers with OpenSAFELY data access agreements to write and execute code for data management and data analysis without direct access to the underlying raw pseudonymised patient data, and to review the outputs of this code. All code for the full data management pipeline, from raw data to completed results for this analysis, and for the OpenSAFELY platform as a whole is available for review at github.com/OpenSAFELY. The data management and analysis code for this paper was led by MDR and contributed to by JBG.

Information governance: NHS England is the data controller of the NHS England OpenSAFELY COVID-19 Service; TPP is the data processor; all study authors using OpenSAFELY have the approval of NHS England.^60^ This implementation of OpenSAFELY is hosted within the TPP environment, which is accredited to the ISO 27001 information security standard and is NHS IG Toolkit compliant.^61^ Patient data have been pseudonymised for analysis and linkage using industry standard cryptographic hashing techniques; all pseudonymised datasets transmitted for linkage onto OpenSAFELY are encrypted; access to the NHS England OpenSAFELY COVID-19 service is through a virtual private network connection; the researchers hold contracts with NHS England and only access the platform to initiate database queries and statistical models; all database activity is logged; only aggregate statistical outputs leave the platform environment following best practice for anonymisation of results such as statistical disclosure control for low cell counts.^62^ The service adheres to the obligations of the UK General Data Protection Regulation and the Data Protection Act 2018. The service previously operated under notices initially issued in February 2020 by the secretary of state under Regulation 3(4) of the Health Service (Control of Patient Information) Regulations 2002, which required organisations to process confidential patient information for covid-19 purposes; this set aside the requirement for patient consent.^63^ As of 1 July 2023, the secretary of state has requested that NHS England continue to operate the service under the COVID-19 Directions 2020.^64^ In some cases of data sharing, the common law duty of confidence is met using, for example, patient consent or support from the Health Research Authority Confidentiality Advisory Group.^65^ Taken together, these provide the legal bases to link patient datasets using the service. General practices, which provide access to the primary care data, are required to share relevant health information to support the public health response to the pandemic, and have been informed of how the service operates. This study was supported by Elizabeth Price (national clinical lead for National Early Inflammatory Arthritis Audit) as senior sponsor, and approval to undertake this study under the remit of service evaluation was obtained from King’s College Hospital NHS Foundation Trust.

Transparency: The lead author (the manuscript’s guarantor) affirms that the manuscript is an honest, accurate, and transparent account of the study being reported; that no important aspects of the study have been omitted; and that any discrepancies from the study as originally planned have been explained.

Dissemination to participants and related patient and public communities: The results of this study will be disseminated to the public through diverse channels, including social media, to promote broad engagement and reach. Feedback will be essential to help guide how to address the identified inequities in diagnosis rates for the studied long term conditions.

Provenance and peer review: Not commissioned; externally peer reviewed.
