# Supplementary material for: Time trends in newly recorded diagnoses of 19 long term conditions before, during, and after the covid-19 pandemic: population based cohort study in England using OpenSAFELY
Source: BMJ. 2026 Jan 22;392:e086393. doi: 10.1136/bmj-2025-086393 (PMC12821157; doi:10.1136/bmj-2025-086393)
Supplement: Supplementary file 1 — Supplementary information: Additional figures S1-S27, tables S1-S3, methods, data, and references [file rusm086393.ww1.pdf]

Supplementary Appendix Contents

Supplementary Figure S1. Monthly rates of newly recorded diagnoses for 19 long-term conditions in England between April 1, 2016, to November 30, 2024..... 3

Supplementary Figure S2. Comparison between crude and adjusted rates of new diagnoses for 19 long-term conditions in England between April 1, 2016, to November 30, 2024. 4

Supplementary Figure S3. Monthly diagnosis rates for depression diagnoses, comparing trends with and without the inclusion of additional diagnostic codes for depressive symptoms. 5

Supplementary Figure S4. Monthly diagnosis rates by age band for 19 long-term conditions in England between April 1, 2016, to November 30, 2024..... 6

Supplementary Figure S5. Monthly diagnosis rates by ethnicity for 11 long-term conditions in England between April 1, 2016, to November 30, 2024..... 7

Supplementary Figure S6. Annual prevalence rates for 19 long-term conditions in England between April 1, 2016, and March 31, 2023..... 8

Supplementary Figure S7. Comparison between crude and age and sex-adjusted prevalence rates for 19 long-term conditions in England between April 1, 2016, and March 31, 2023. 9

Supplementary Figure S8. Sensitivity analysis using Prophet to forecast rates of new diagnoses for 19 long-term conditions in England between April 1, 2016, to November 30, 2024..... 10

Supplementary Figure S9. Model validation and residual diagnostics for the SARIMA model utilised for asthma..... 11

Supplementary Figure S10. Model validation and residual diagnostics for the SARIMA model utilised for atopic dermatitis. 12

Supplementary Figure S11. Model validation and residual diagnostics for the SARIMA model utilised for coronary heart disease. 13

Supplementary Figure S12. Model validation and residual diagnostics for the SARIMA model utilised for chronic kidney disease..... 14

Supplementary Figure S13. Model validation and residual diagnostics for the SARIMA model utilised for coeliac disease. 15

Supplementary Figure S14. Model validation and residual diagnostics for the SARIMA model utilised for chronic obstructive pulmonary disease..... 16

Supplementary Figure S15. Model validation and residual diagnostics for the SARIMA model utilised for Crohn's disease..... 17

Supplementary Figure S16. Model validation and residual diagnostics for the SARIMA model utilised for dementia. 18

Supplementary Figure S17. Model validation and residual diagnostics for the SARIMA model utilised for depression..... 19

Supplementary Figure S18. Model validation and residual diagnostics for the SARIMA model utilised for type 2 diabetes mellitus. 20

Supplementary Figure S19. Model validation and residual diagnostics for the SARIMA model utilised for epilepsy..... 21

Supplementary Figure S20. Model validation and residual diagnostics for the SARIMA model utilised for heart failure..... 22

Supplementary Figure S21. Model validation and residual diagnostics for the SARIMA model utilised for multiple sclerosis. 23

Supplementary Figure S22. Model validation and residual diagnostics for the SARIMA model utilised for osteoporosis. 24

Supplementary Figure S23. Model validation and residual diagnostics for the SARIMA model utilised for polymyalgia rheumatica. 25

Supplementary Figure S24. Model validation and residual diagnostics for the SARIMA model utilised for psoriasis. 26

Supplementary Figure S25. Model validation and residual diagnostics for the SARIMA model utilised for rheumatoid arthritis. 27

Supplementary Figure S26. Model validation and residual diagnostics for the SARIMA model utilised for stroke and transient ischaemic attacks. 28

Supplementary Figure S27. Model validation and residual diagnostics for the SARIMA model utilised for ulcerative colitis. 29

Supplementary Table S1. Sociodemographic characteristics for the reference population and for individuals with newly recorded diagnoses of 19 long-term health conditions. 30

Supplementary Table S2. Yearly differences between expected and observed diagnosis rates for 19 long-term conditions after the onset of the COVID-19 pandemic in England. 32

Supplementary Table S3. Sensitivity analysis using Prophet to forecast differences between expected and observed rates of new diagnoses for 19 long-term conditions in England after the onset of the COVID-19 pandemic. 34

Supplementary Methods: Autoregressive Integrated Moving Average (ARIMA) modelling 36

Supplementary Data: Diagnostic codelists 38

References 44

Supplementary Figure S1. Monthly rates of newly recorded diagnoses for 19 long-term conditions in England between April 1, 2016, to November 30, 2024.

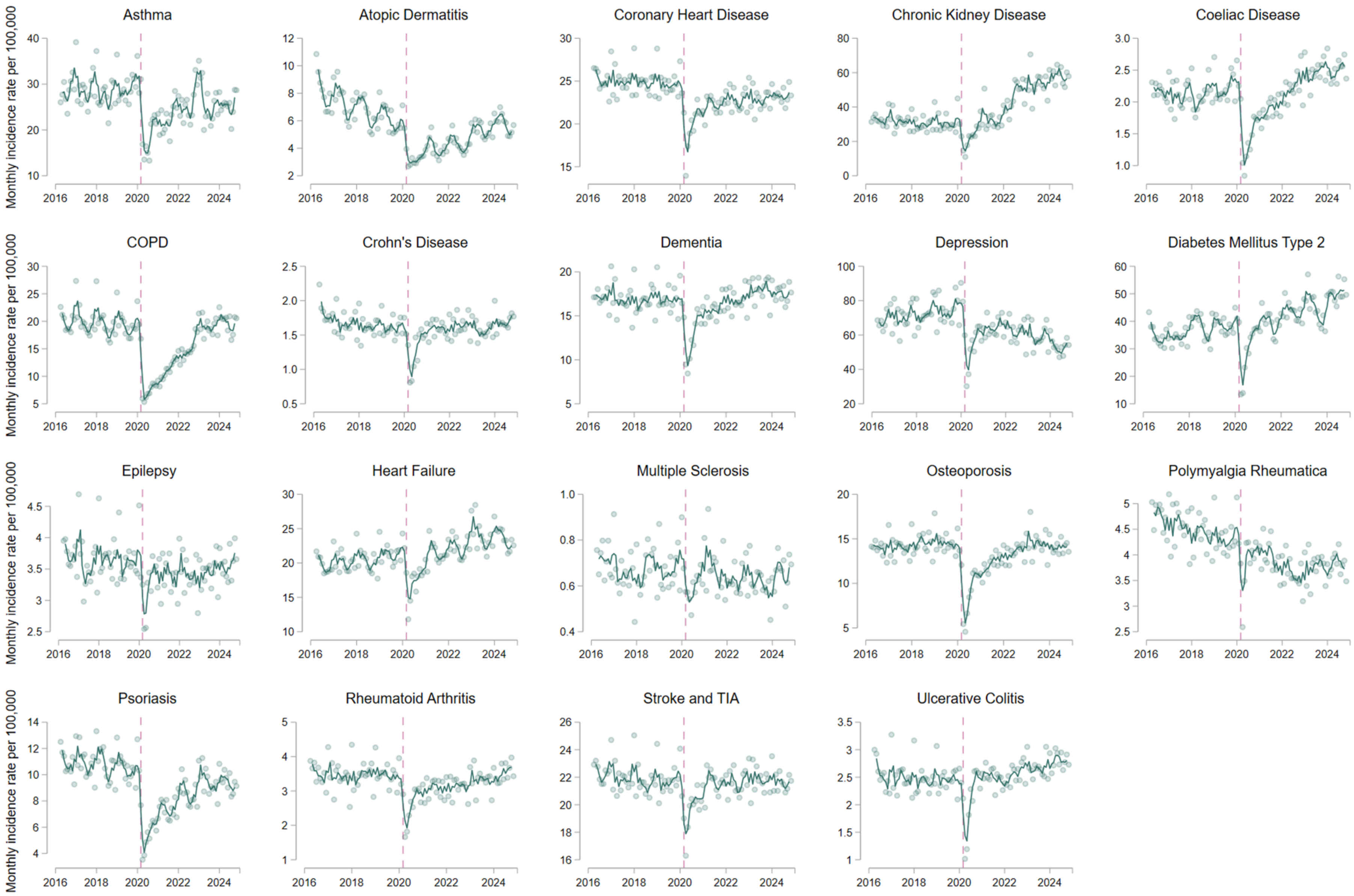

Age and sex-adjusted rates of new diagnoses are shown per 100,000 population. Individual data points represent monthly diagnosis rates for each condition, which are presented alongside 3-monthly rolling averages of the current, preceding and subsequent months. The vertical dashed lines correspond to the onset of the first COVID-19 lockdown in England (March 2020).

Supplementary Figure S2. Comparison between crude and adjusted rates of new diagnoses for 19 long-term conditions in England between April 1, 2016, to November 30, 2024.

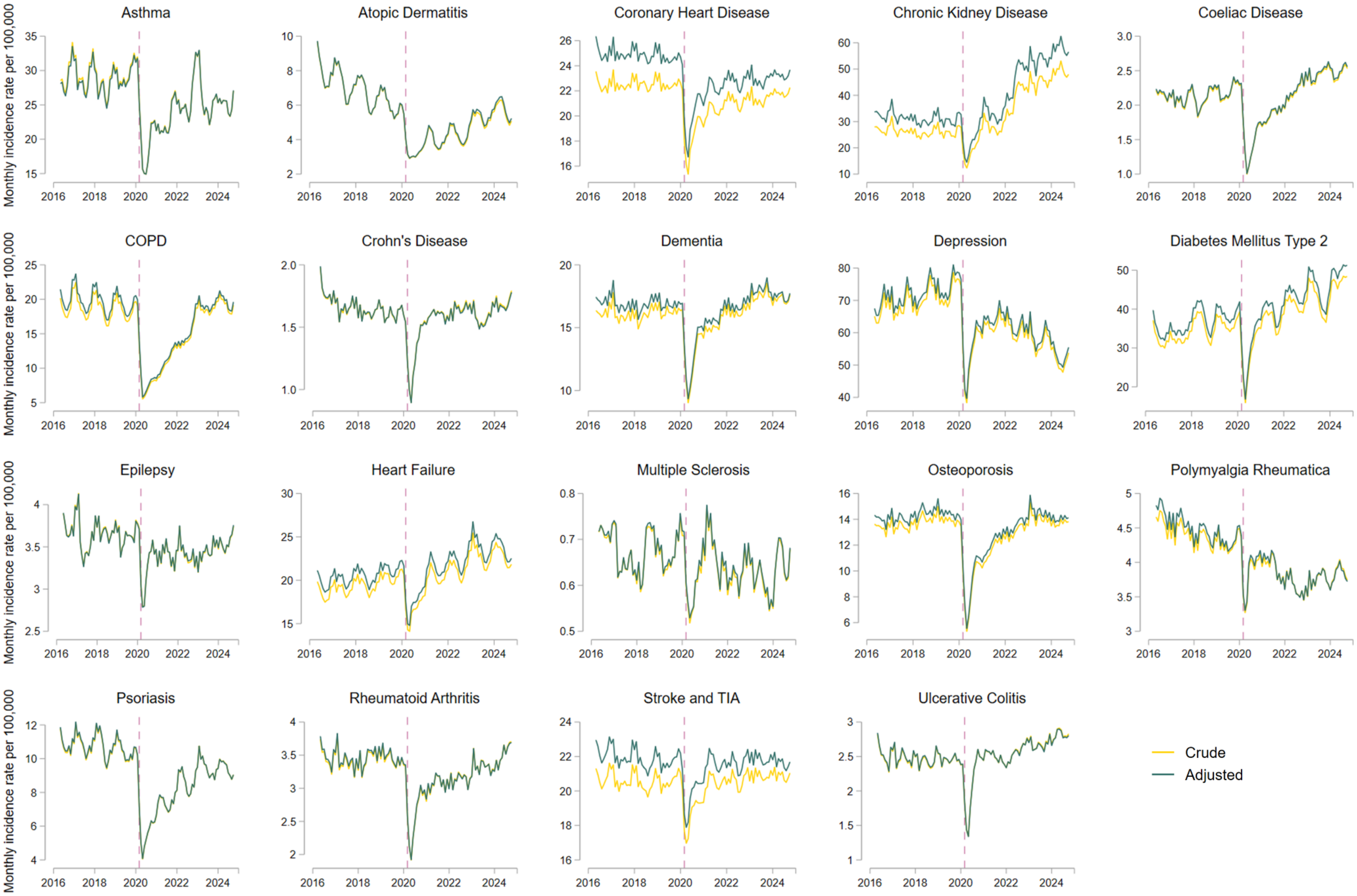

Crude (yellow) and age and sex-adjusted (green) diagnosis rates are shown per 100,000 population as 3-monthly rolling averages of the current, preceding and subsequent months. The vertical dashed lines correspond to the onset of the first COVID-19 lockdown in England (March 2020).

Supplementary Figure S3. Monthly diagnosis rates for depression diagnoses, comparing trends with and without the inclusion of additional diagnostic codes for depressive symptoms.

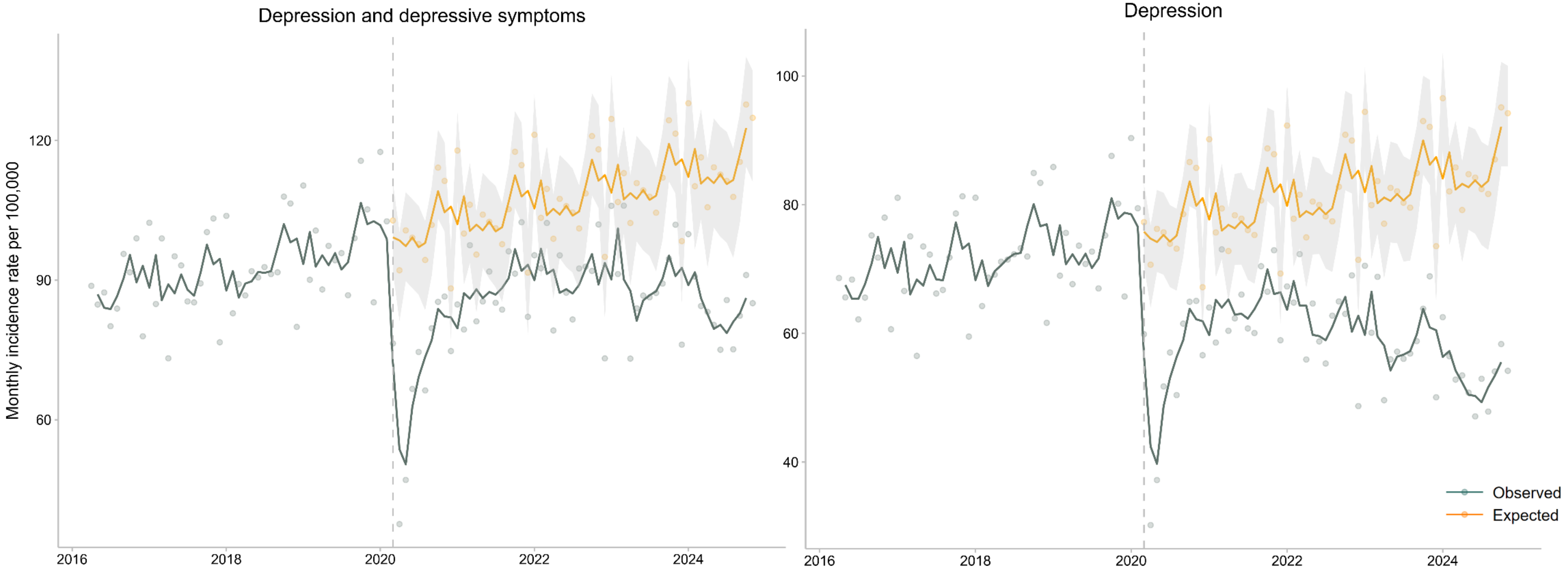

Sensitivity analysis comparing observed (dark green) and expected (orange) rates of newly recorded diagnoses of depression in England after the onset of the COVID-19 pandemic. On the left-hand panel, an expanded depression codelist was used, which included symptomatic codes suggestive of depression (e.g. depressed mood) as well as more definitive depression diagnostic codes (e.g. depression). On the right-hand panel, only definitive depression diagnostic codes were included. Expected diagnosis rates after the onset of the pandemic (March 2020; vertical dashed lines) were estimated using seasonal autoregressive integrated moving averages (SARIMA) models, utilising data from April 1, 2016, to February 28, 2020. Individual data points represent age and sex-adjusted monthly diagnosis rates per 100,000 population, which are presented alongside 3-monthly rolling averages of the current, preceding and subsequent months.

Supplementary Figure S4. Monthly diagnosis rates by age band for 19 long-term conditions in England between April 1, 2016, to November 30, 2024.

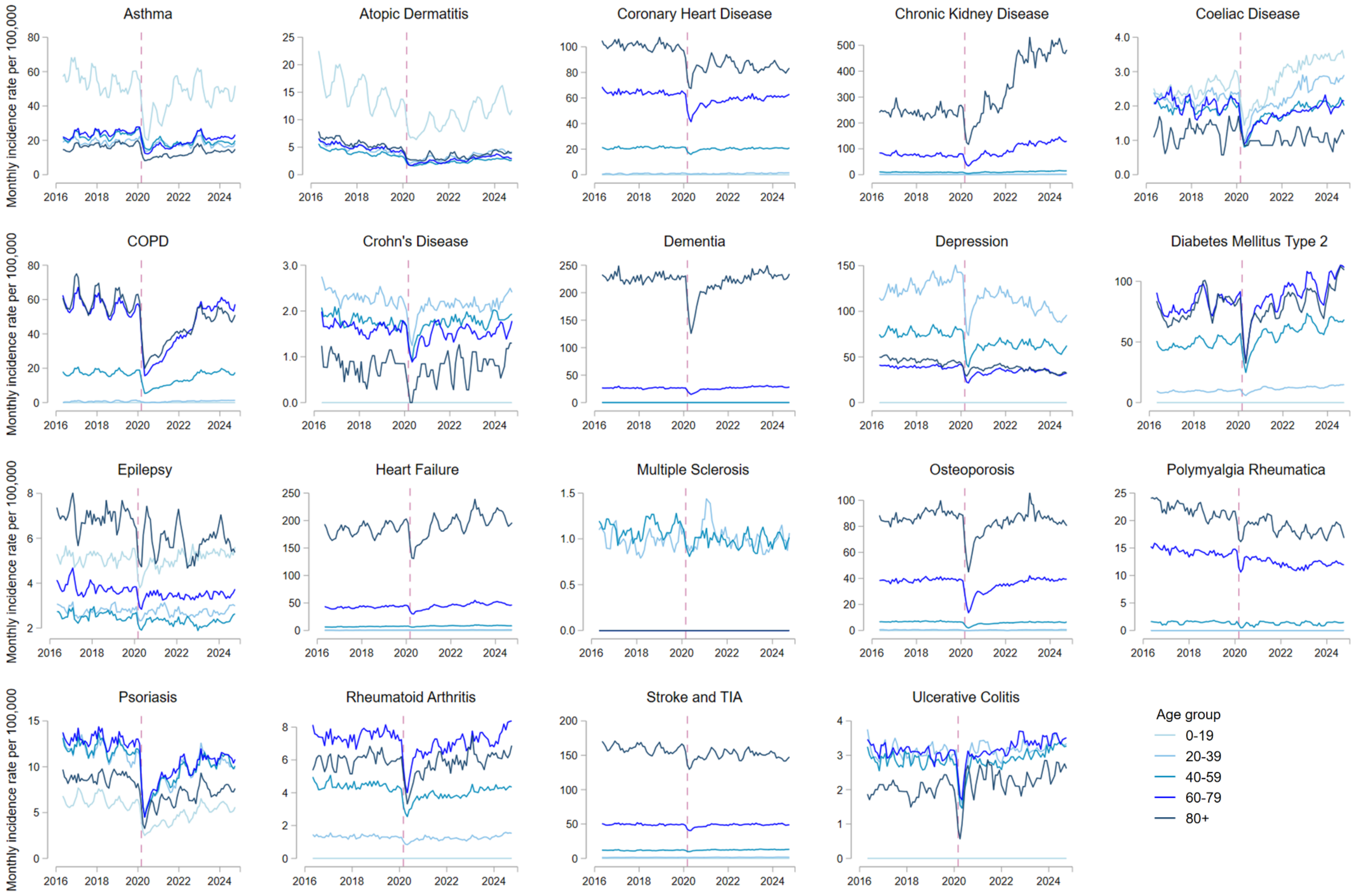

Monthly rates of new diagnoses per 100,000 population are shown for each condition, separated by age band (0-19, 20-39, 40-59, 60-79 and 80+ years). Rates are presented as 3-monthly rolling averages of the current, preceding and subsequent months. The vertical dashed lines correspond to the onset of the first COVID-19 lockdown in England (March 2020). Small count suppression was applied to minimise the risk of disclosure.

Supplementary Figure S5. Monthly diagnosis rates by ethnicity for 11 long-term conditions in England between April 1, 2016, to November 30, 2024.

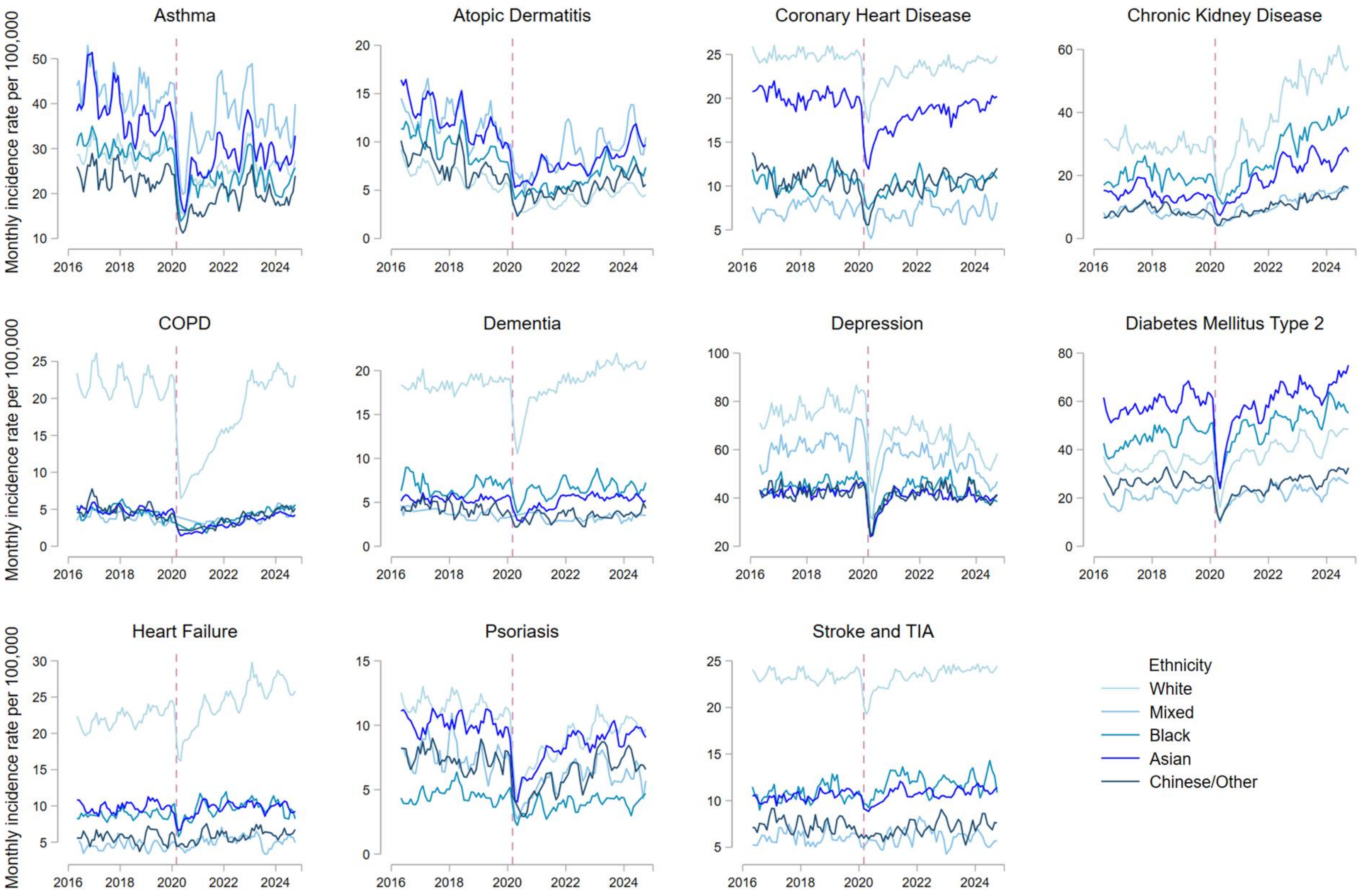

Monthly rates of new diagnoses per 100,000 population are shown for each condition, separated by ethnicity (categorised into White, Mixed, Asian or Asian British, Black or Black British, or Chinese/Other ethnic groups). Rates are presented as 3-monthly rolling averages of the current, preceding and subsequent months. The vertical dashed lines correspond to the onset of the first COVID-19 lockdown in England (March 2020). No age or sex-standardisation was performed for computational reasons. Conditions with small numbers of new diagnoses when analysed separately by ethnicity (coeliac disease, Crohn's disease, epilepsy, multiple sclerosis, osteoporosis, polymyalgia rheumatica, rheumatoid arthritis and ulcerative colitis) are not shown due to the potential for disclosure.

Supplementary Figure S6. Annual prevalence rates for 19 long-term conditions in England between April 1, 2016, and March 31, 2023.

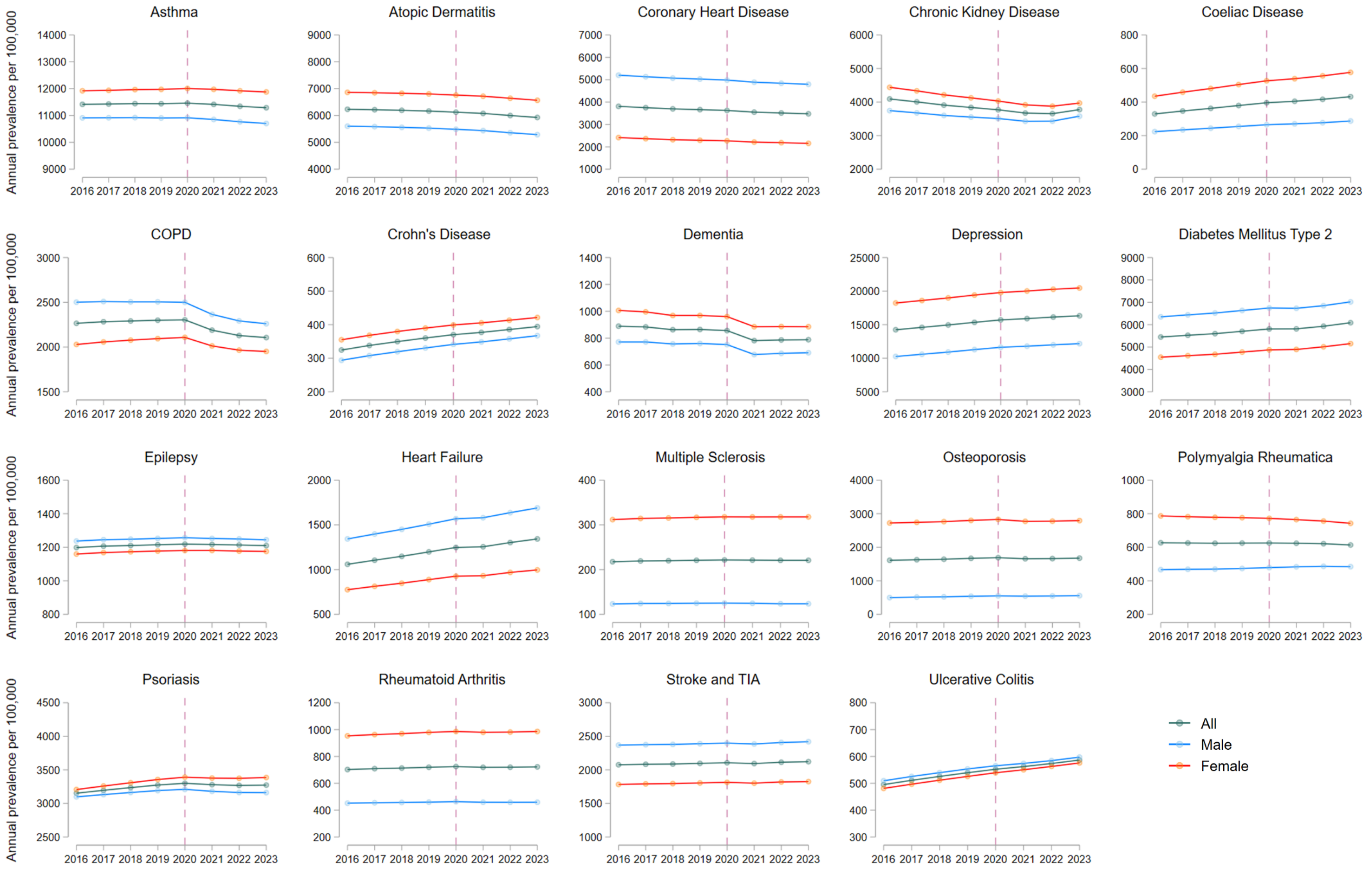

Age and sex-adjusted annual prevalence rates per 100,000 population are shown for each condition overall (green) and separately for males (blue) and females (red). The vertical dashed lines correspond to the onset of the first COVID-19 lockdown in England (March 2020).

Supplementary Figure S7. Comparison between crude and age and sex-adjusted prevalence rates for 19 long-term conditions in England between April 1, 2016, and March 31, 2023.

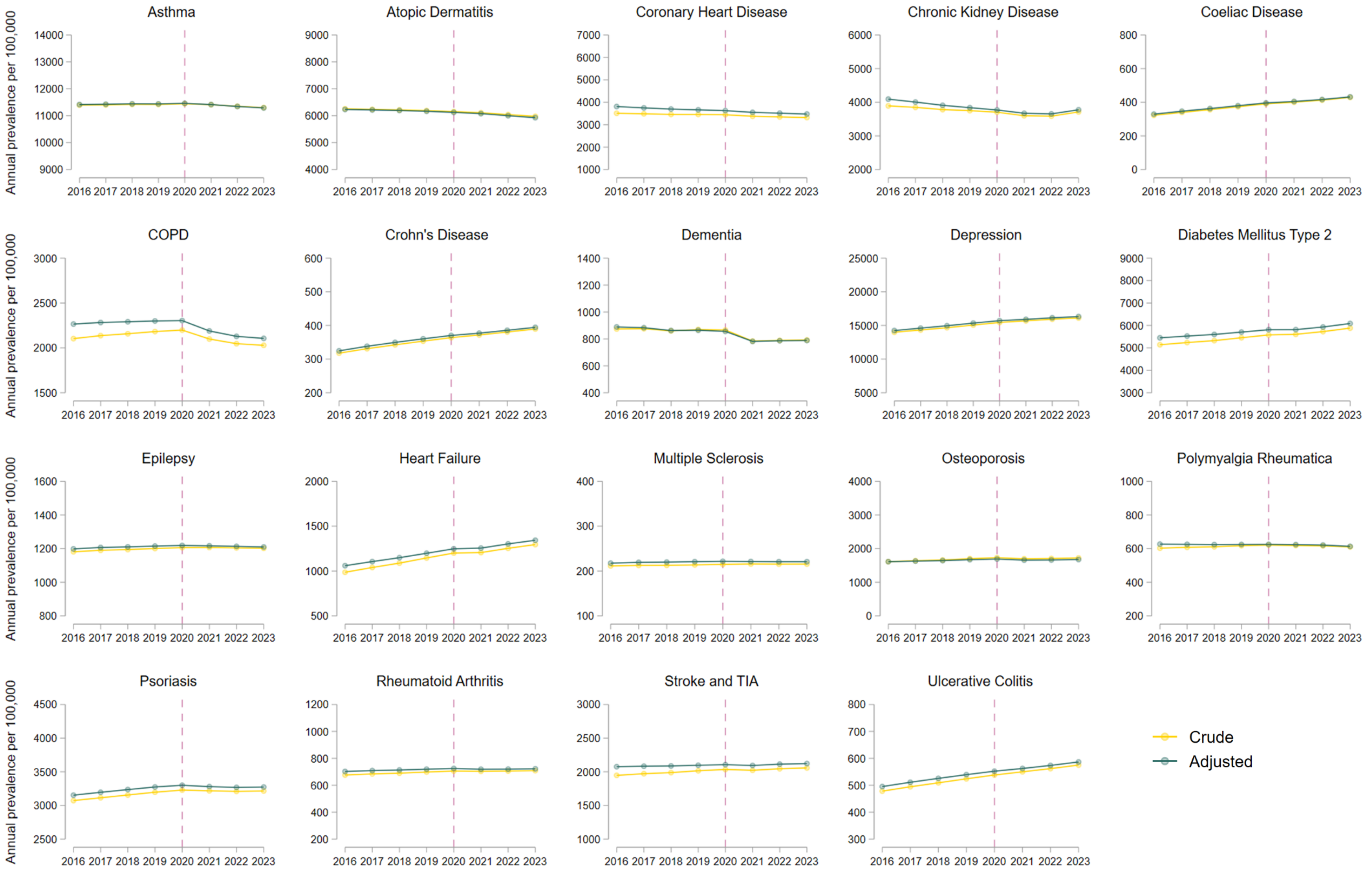

Crude (yellow) and age and sex-adjusted (green) annual prevalence rates per 100,000 population are shown for each condition. The vertical dashed lines correspond to the onset of the first COVID-19 lockdown in England (March 2020).

Supplementary Figure S8. Sensitivity analysis using Prophet to forecast rates of new diagnoses for 19 long-term conditions in England between April 1, 2016, to November 30, 2024.

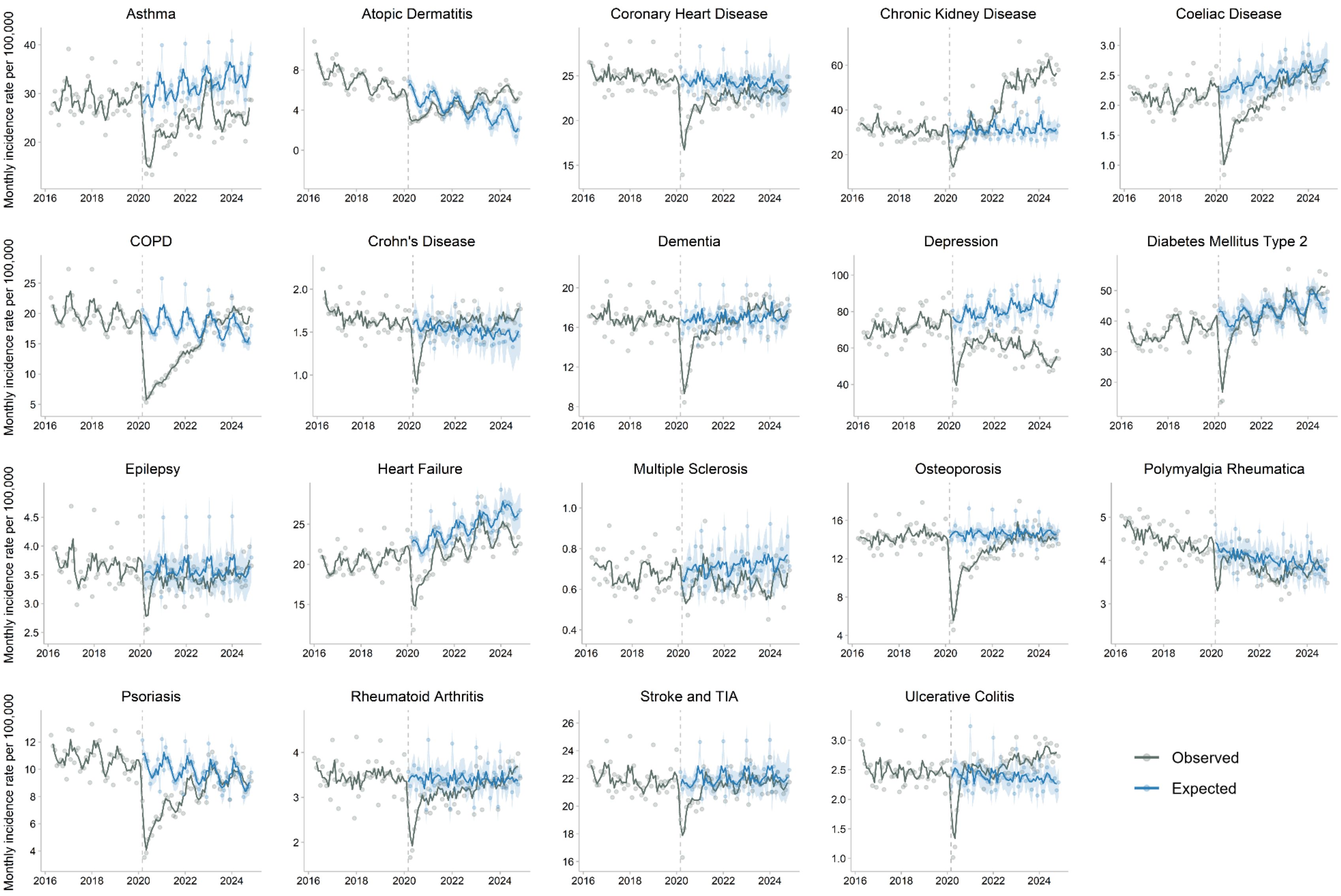

Comparison between observed (dark green) and expected (blue) rates of newly recorded diagnoses for 19 long-term conditions after the onset of the COVID-19 pandemic in England. Expected diagnosis rates after March 2020 (vertical dashed lines) were estimated using Prophet forecasting methodology, utilising data from April 1, 2016, to February 28, 2020. Individual data points are shown, representing age and sex-adjusted monthly diagnosis rates per 100,000 population, in addition to 3-monthly rolling averages of the current, preceding and subsequent months. 95% prediction intervals (shaded in light blue) are shown around the estimates of expected diagnosis rates.

Supplementary Figure S9. Model validation and residual diagnostics for the SARIMA model utilised for asthma.

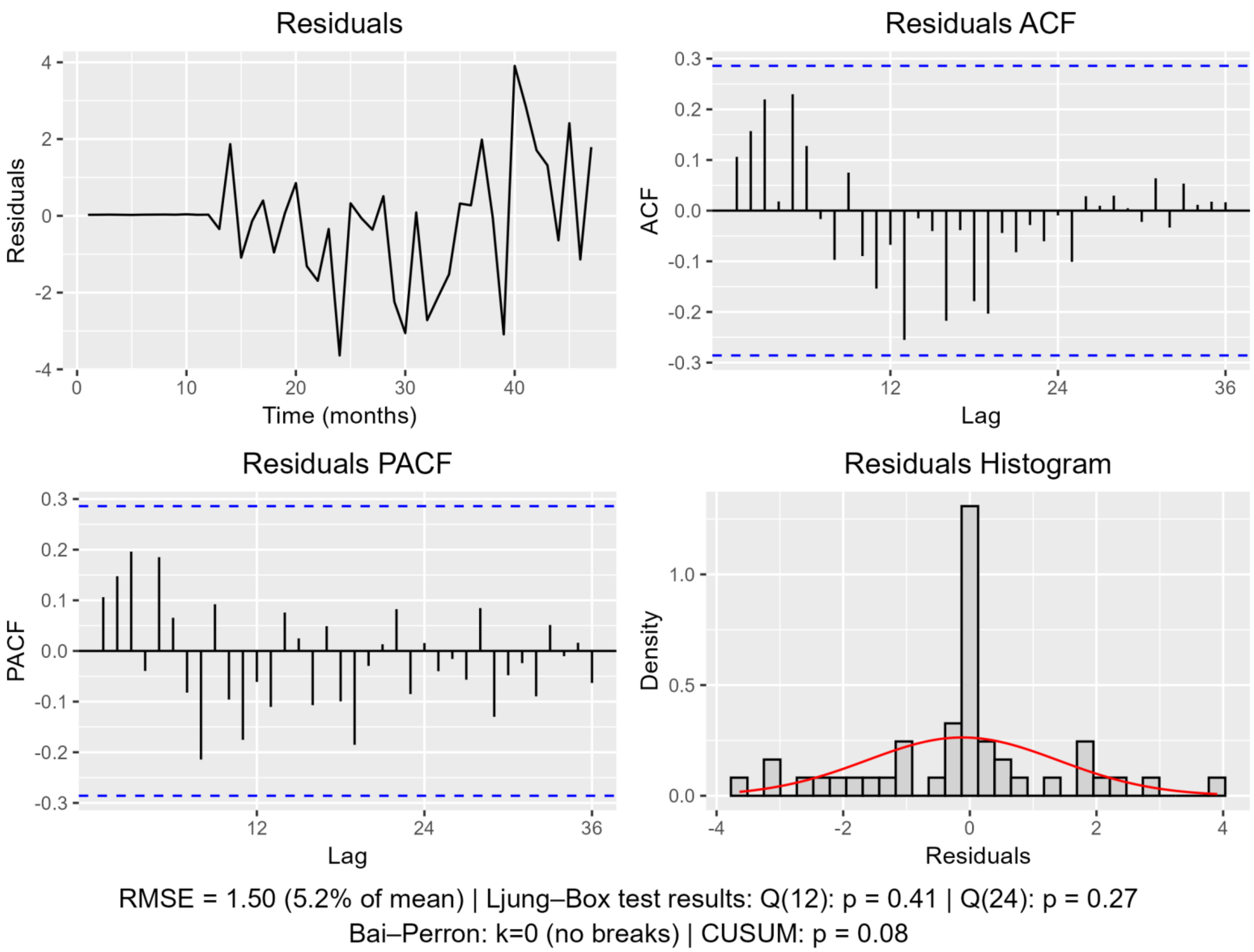

Residual diagnostics are shown for ARIMA(0,0,0)(0,1,1)[12], including plots of residuals over time (top left), autocorrelation (ACF) plots of residuals (top right), partial autocorrelation (PACF) plots of residuals (bottom left), and residual histograms (bottom right). The root mean squared error (RMSE) for the model is shown in absolute terms and as a percentage of the series mean (normalised RMSE). Ljung–Box test results at 12 and 24 lags are shown, assessing for residual autocorrelation. Bai-Perron tests were used to detect structural breaks in pre-pandemic time series, with the optimal number of breaks (k) determined based upon the Bayesian Information Criterion. Cumulative Sum (CUSUM) tests were used to assess parameter stability over time.

Supplementary Figure S10. Model validation and residual diagnostics for the SARIMA model utilised for atopic dermatitis.

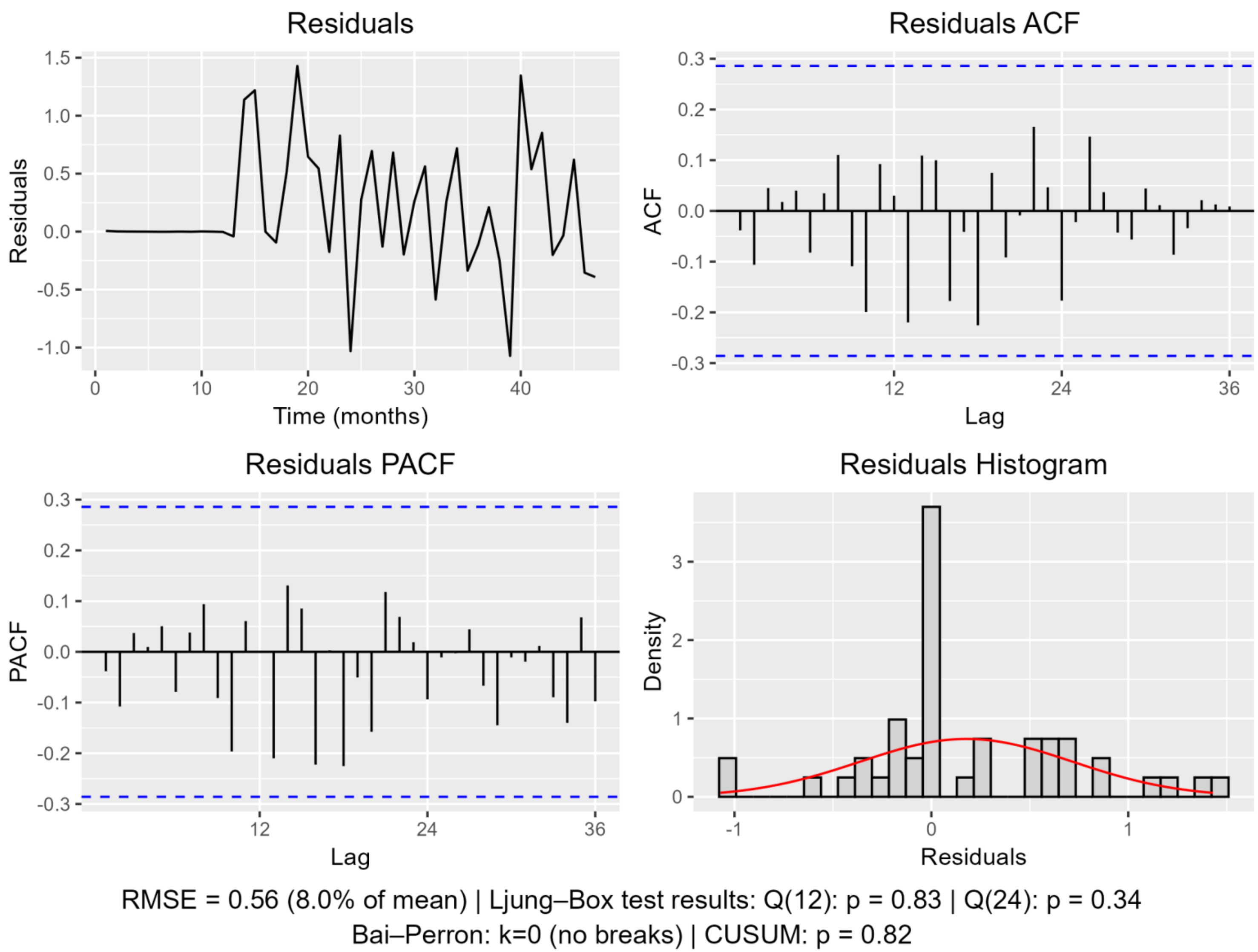

Residual diagnostics are shown for ARIMA(0,1,1)(0,1,1)[12], including plots of residuals over time (top left), autocorrelation (ACF) plots of residuals (top right), partial autocorrelation (PACF) plots of residuals (bottom left), and residual histograms (bottom right). The root mean squared error (RMSE) for the model is shown in absolute terms and as a percentage of the series mean (normalised RMSE). Ljung–Box test results at 12 and 24 lags are shown, assessing for residual autocorrelation. Bai-Perron tests were used to detect structural breaks in pre-pandemic time series, with the optimal number of breaks (k) determined based upon the Bayesian Information Criterion. Cumulative Sum (CUSUM) tests were used to assess parameter stability over time.

Supplementary Figure S11. Model validation and residual diagnostics for the SARIMA model utilised for coronary heart disease.

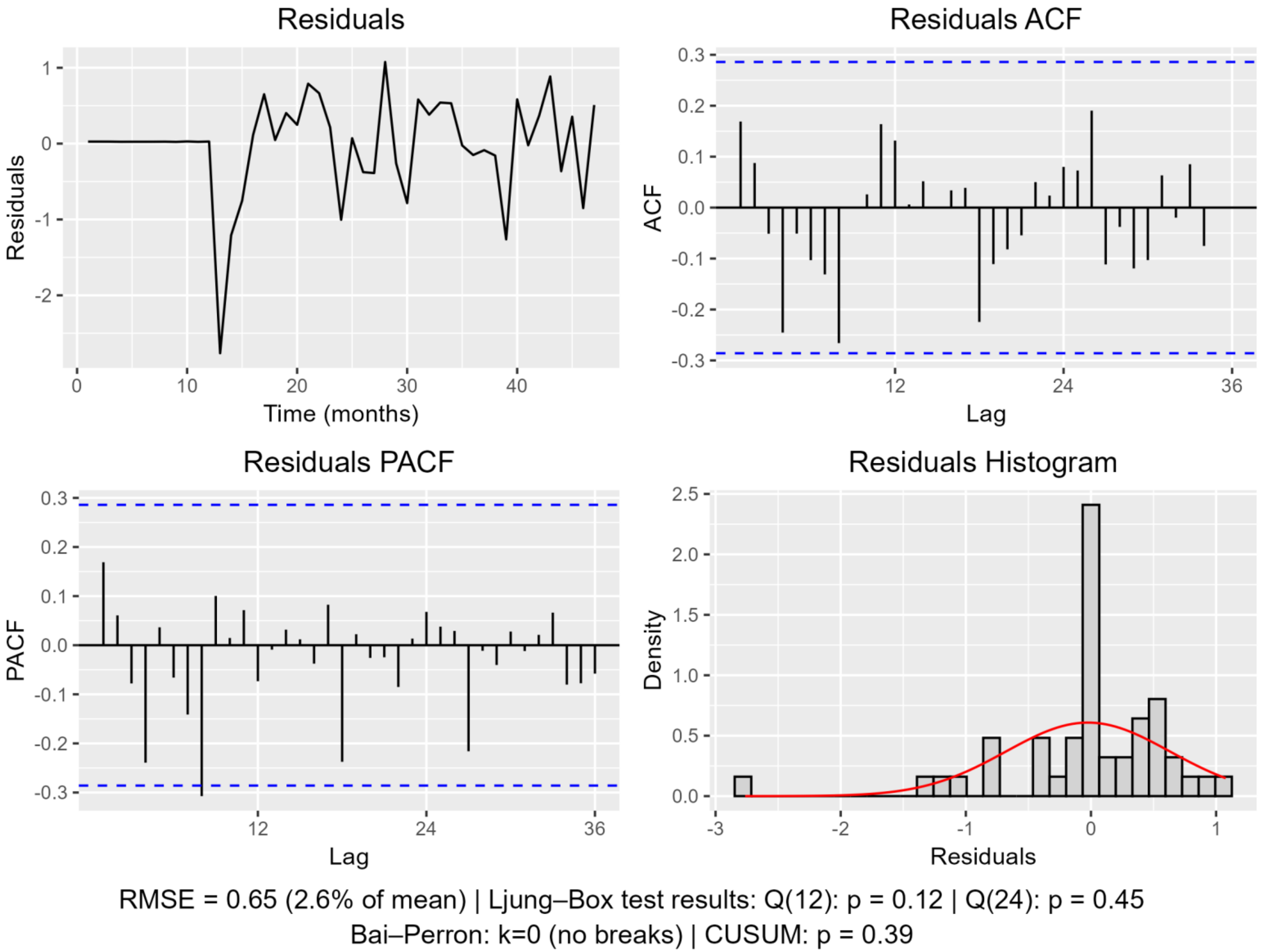

Residual diagnostics are shown for ARIMA(1,0,0)(0,1,1)[12] with drift, including plots of residuals over time (top left), autocorrelation (ACF) plots of residuals (top right), partial autocorrelation (PACF) plots of residuals (bottom left), and residual histograms (bottom right). The root mean squared error (RMSE) for the model is shown in absolute terms and as a percentage of the series mean (normalised RMSE). Ljung–Box test results at 12 and 24 lags are shown, assessing for residual autocorrelation. Bai-Perron tests were used to detect structural breaks in pre-pandemic time series, with the optimal number of breaks (k) determined based upon the Bayesian Information Criterion. Cumulative Sum (CUSUM) tests were used to assess parameter stability over time.

Supplementary Figure S12. Model validation and residual diagnostics for the SARIMA model utilised for chronic kidney disease.

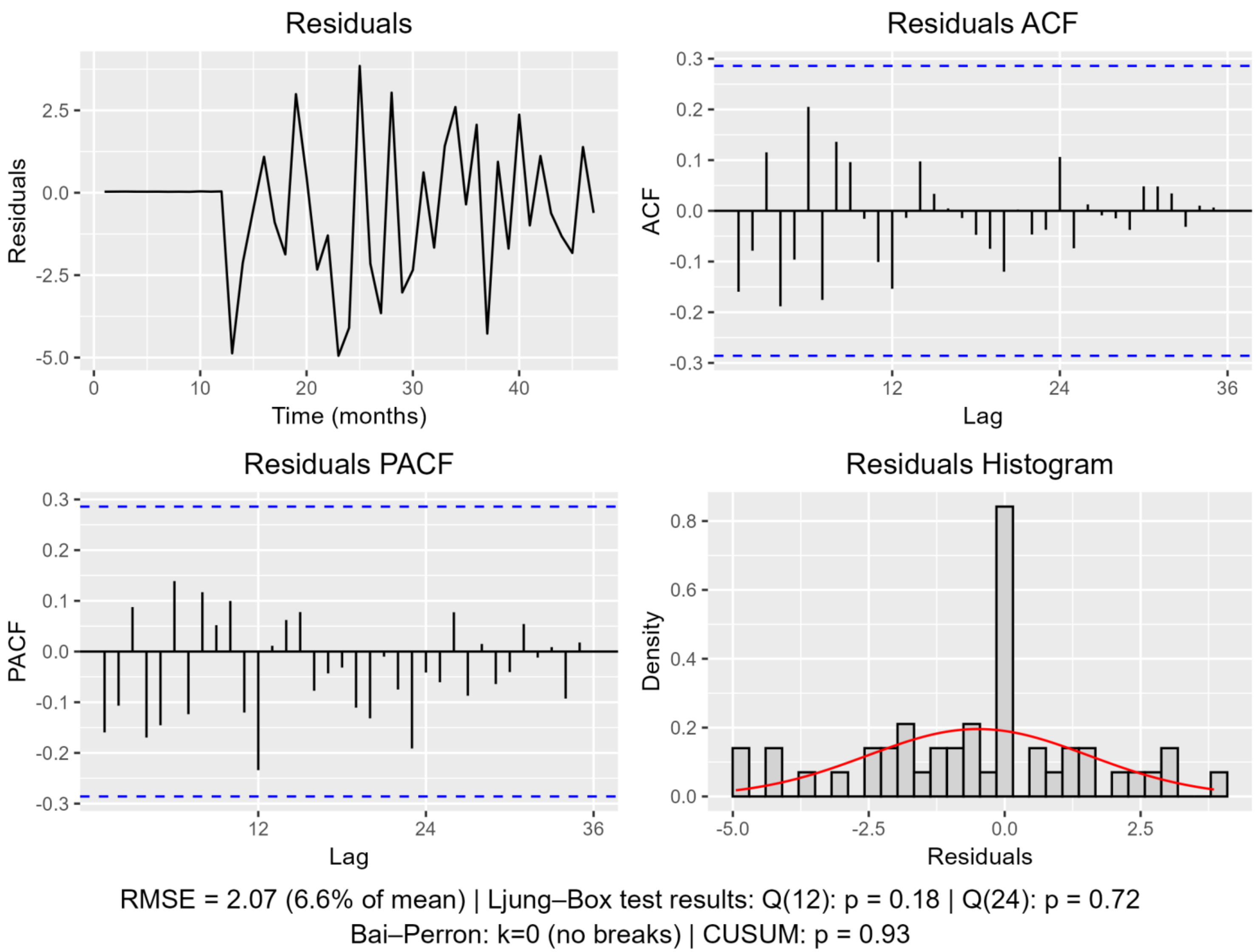

Residual diagnostics are shown for ARIMA(0,0,1)(0,1,2)[12], including plots of residuals over time (top left), autocorrelation (ACF) plots of residuals (top right), partial autocorrelation (PACF) plots of residuals (bottom left), and residual histograms (bottom right). The root mean squared error (RMSE) for the model is shown in absolute terms and as a percentage of the series mean (normalised RMSE). Ljung–Box test results at 12 and 24 lags are shown, assessing for residual autocorrelation. Bai-Perron tests were used to detect structural breaks in pre-pandemic time series, with the optimal number of breaks (k) determined based upon the Bayesian Information Criterion. Cumulative Sum (CUSUM) tests were used to assess parameter stability over time.

Supplementary Figure S13. Model validation and residual diagnostics for the SARIMA model utilised for coeliac disease.

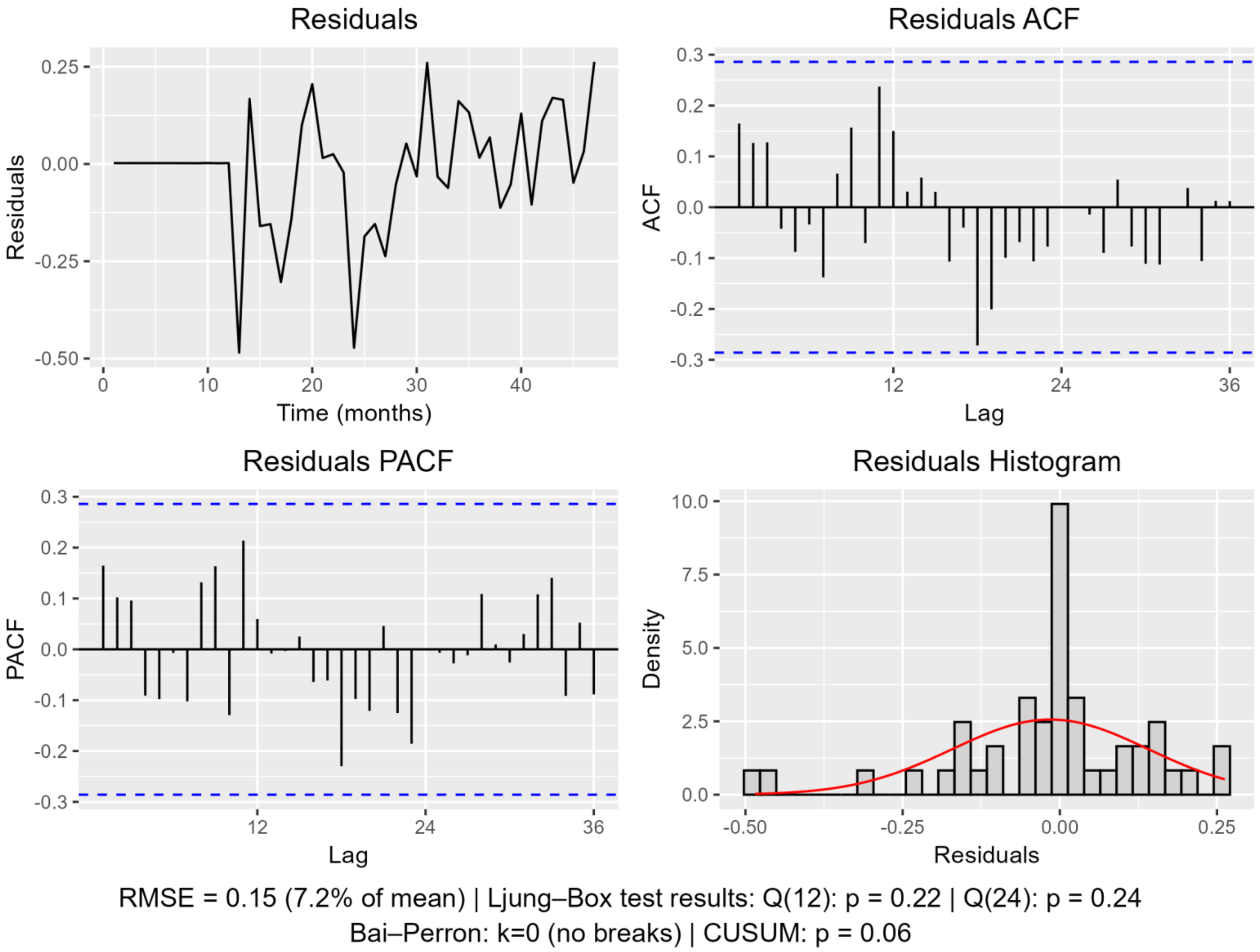

Residual diagnostics are shown for ARIMA(0,0,0)(1,1,1)[12] with drift, including plots of residuals over time (top left), autocorrelation (ACF) plots of residuals (top right), partial autocorrelation (PACF) plots of residuals (bottom left), and residual histograms (bottom right). The root mean squared error (RMSE) for the model is shown in absolute terms and as a percentage of the series mean (normalised RMSE). Ljung–Box test results at 12 and 24 lags are shown, assessing for residual autocorrelation. Bai-Perron tests were used to detect structural breaks in pre-pandemic time series, with the optimal number of breaks (k) determined based upon the Bayesian Information Criterion. Cumulative Sum (CUSUM) tests were used to assess parameter stability over time.

Supplementary Figure S14. Model validation and residual diagnostics for the SARIMA model utilised for chronic obstructive pulmonary disease.

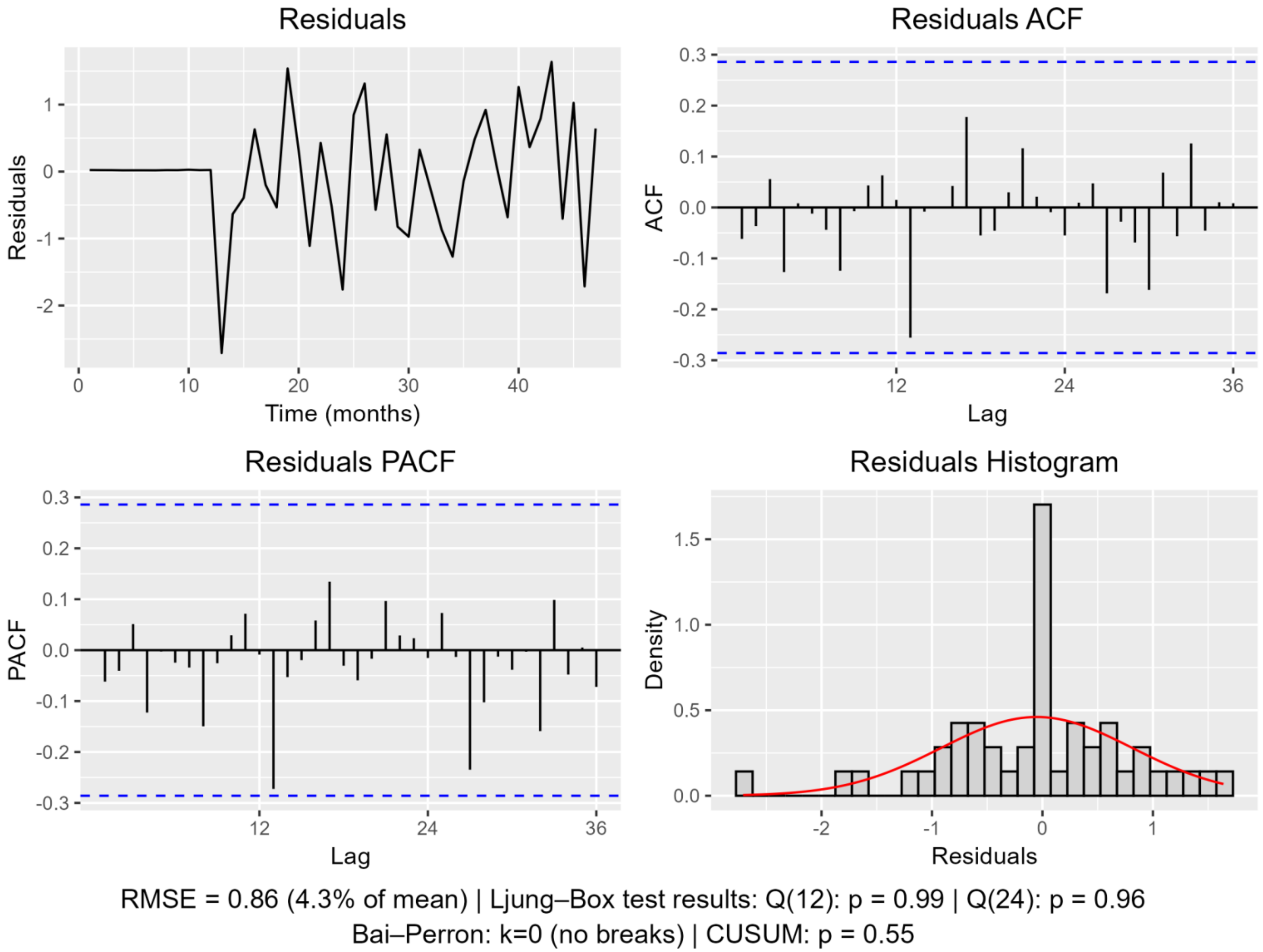

Residual diagnostics are shown for ARIMA(0,0,0)(0,1,1)[12] with drift, including plots of residuals over time (top left), autocorrelation (ACF) plots of residuals (top right), partial autocorrelation (PACF) plots of residuals (bottom left), and residual histograms (bottom right). The root mean squared error (RMSE) for the model is shown in absolute terms and as a percentage of the series mean (normalised RMSE). Ljung–Box test results at 12 and 24 lags are shown, assessing for residual autocorrelation. Bai-Perron tests were used to detect structural breaks in pre-pandemic time series, with the optimal number of breaks (k) determined based upon the Bayesian Information Criterion. Cumulative Sum (CUSUM) tests were used to assess parameter stability over time.

Supplementary Figure S15. Model validation and residual diagnostics for the SARIMA model utilised for Crohn's disease.

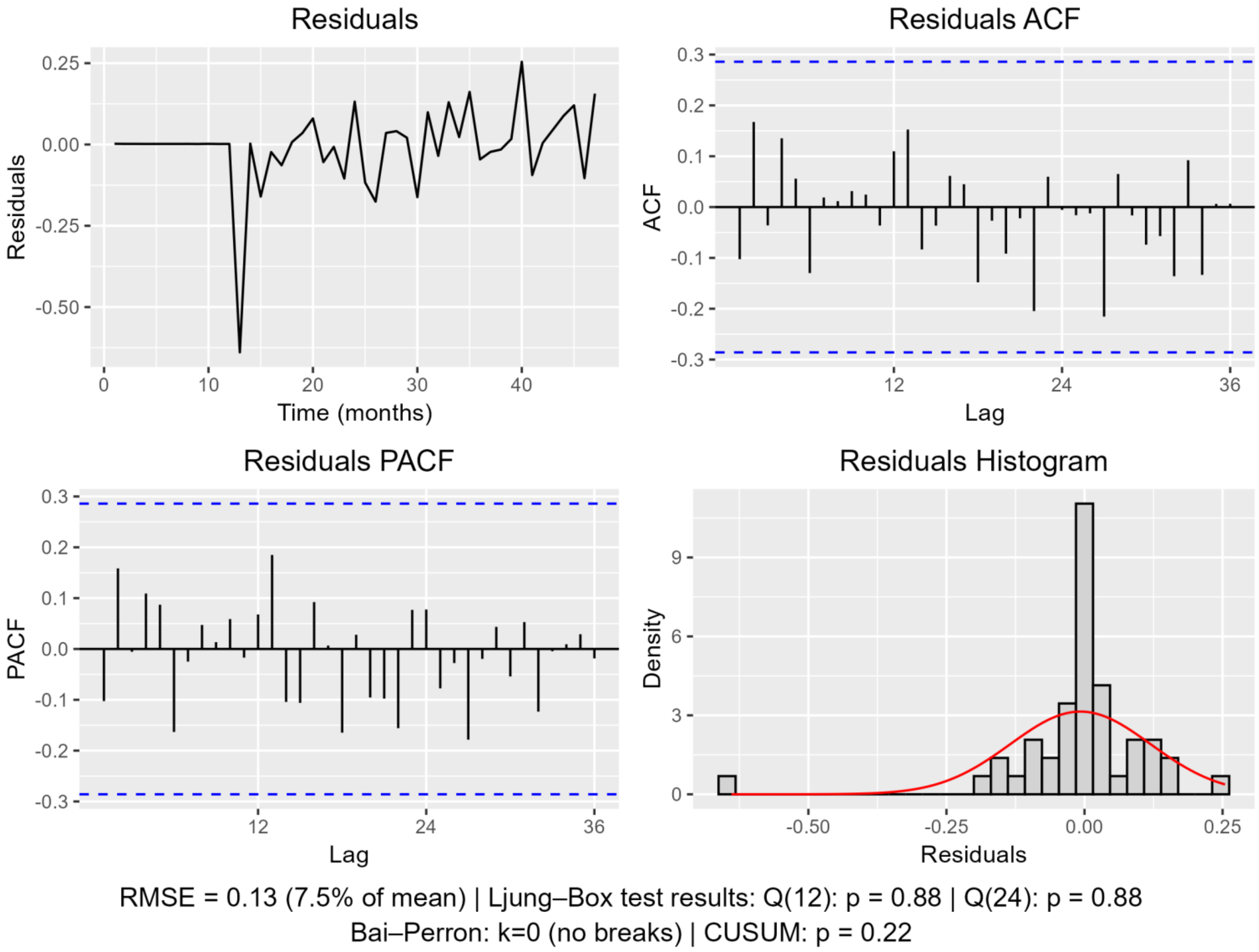

Residual diagnostics are shown for ARIMA(0,0,0)(0,1,1)[12] with drift, including plots of residuals over time (top left), autocorrelation (ACF) plots of residuals (top right), partial autocorrelation (PACF) plots of residuals (bottom left), and residual histograms (bottom right). The root mean squared error (RMSE) for the model is shown in absolute terms and as a percentage of the series mean (normalised RMSE). Ljung–Box test results at 12 and 24 lags are shown, assessing for residual autocorrelation. Bai-Perron tests were used to detect structural breaks in pre-pandemic time series, with the optimal number of breaks (k) determined based upon the Bayesian Information Criterion. Cumulative Sum (CUSUM) tests were used to assess parameter stability over time.

Supplementary Figure S16. Model validation and residual diagnostics for the SARIMA model utilised for dementia.

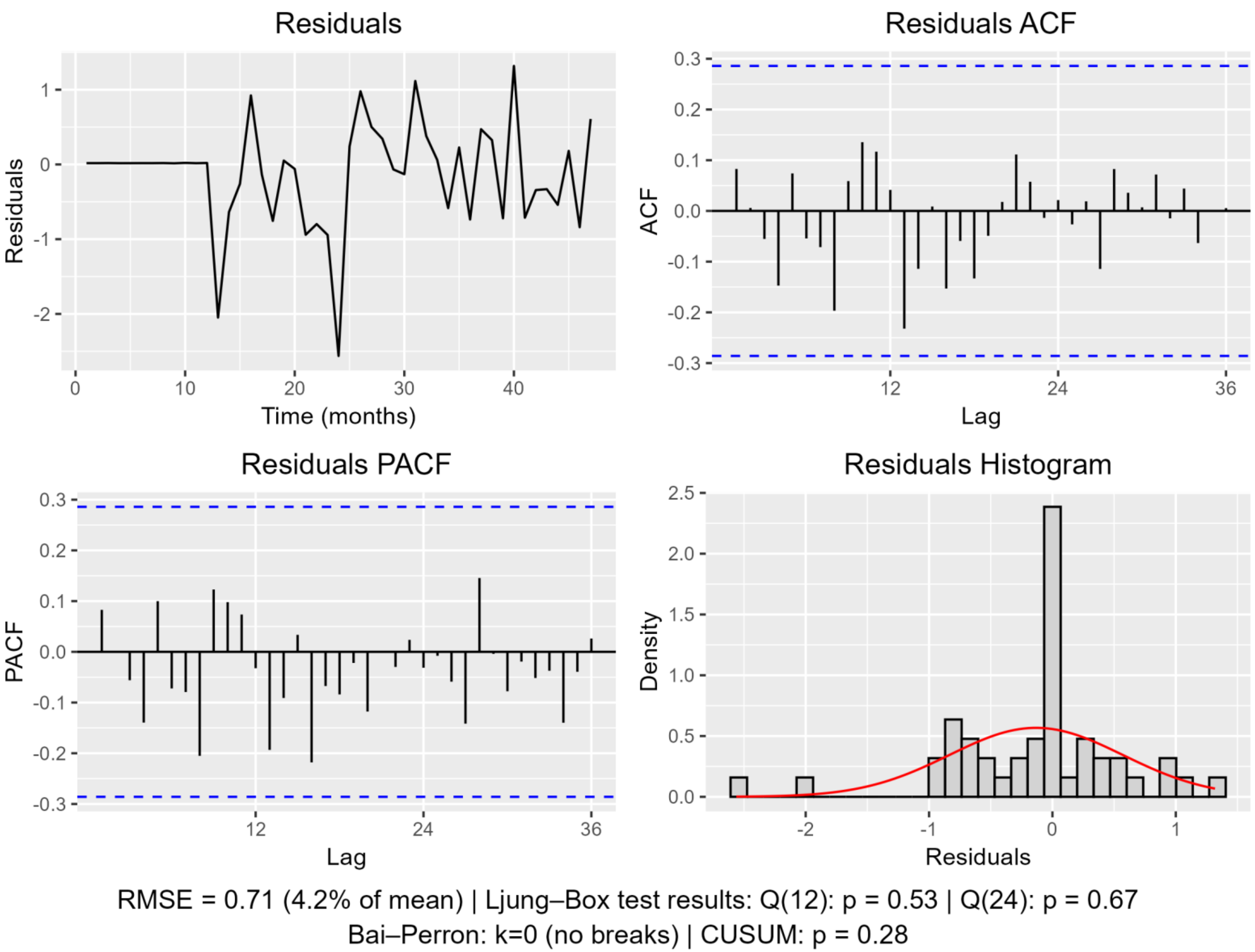

Residual diagnostics are shown for ARIMA(3,0,0)(0,1,1)[12], including plots of residuals over time (top left), autocorrelation (ACF) plots of residuals (top right), partial autocorrelation (PACF) plots of residuals (bottom left), and residual histograms (bottom right). The root mean squared error (RMSE) for the model is shown in absolute terms and as a percentage of the series mean (normalised RMSE). Ljung–Box test results at 12 and 24 lags are shown, assessing for residual autocorrelation. Bai-Perron tests were used to detect structural breaks in pre-pandemic time series, with the optimal number of breaks (k) determined based upon the Bayesian Information Criterion. Cumulative Sum (CUSUM) tests were used to assess parameter stability over time.

Supplementary Figure S17. Model validation and residual diagnostics for the SARIMA model utilised for depression.

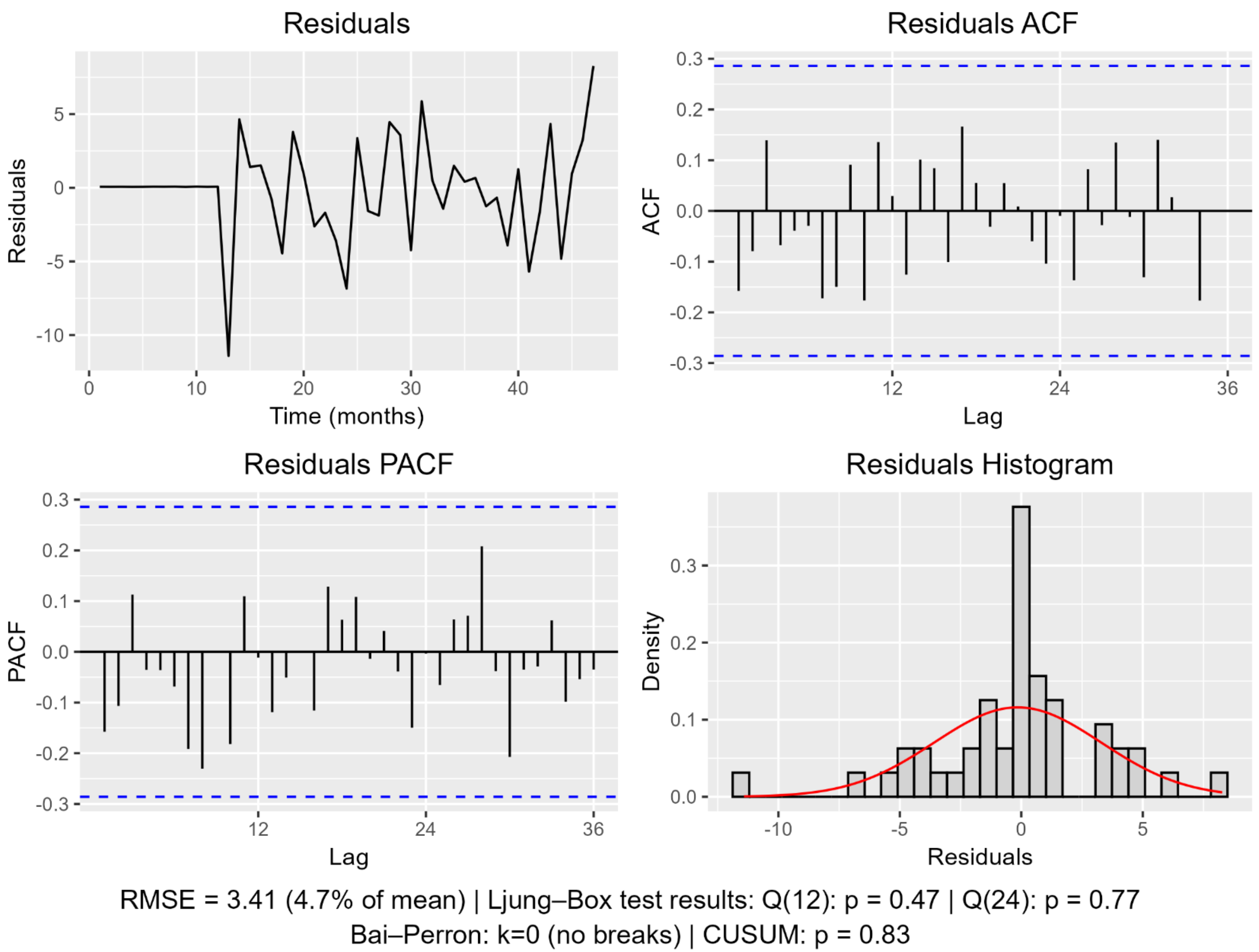

Residual diagnostics are shown for ARIMA(0,0,0)(0,1,1)[12] with drift, including plots of residuals over time (top left), autocorrelation (ACF) plots of residuals (top right), partial autocorrelation (PACF) plots of residuals (bottom left), and residual histograms (bottom right). The root mean squared error (RMSE) for the model is shown in absolute terms and as a percentage of the series mean (normalised RMSE). Ljung–Box test results at 12 and 24 lags are shown, assessing for residual autocorrelation. Bai-Perron tests were used to detect structural breaks in pre-pandemic time series, with the optimal number of breaks (k) determined based upon the Bayesian Information Criterion. Cumulative Sum (CUSUM) tests were used to assess parameter stability over time.

Supplementary Figure S18. Model validation and residual diagnostics for the SARIMA model utilised for type 2 diabetes mellitus.

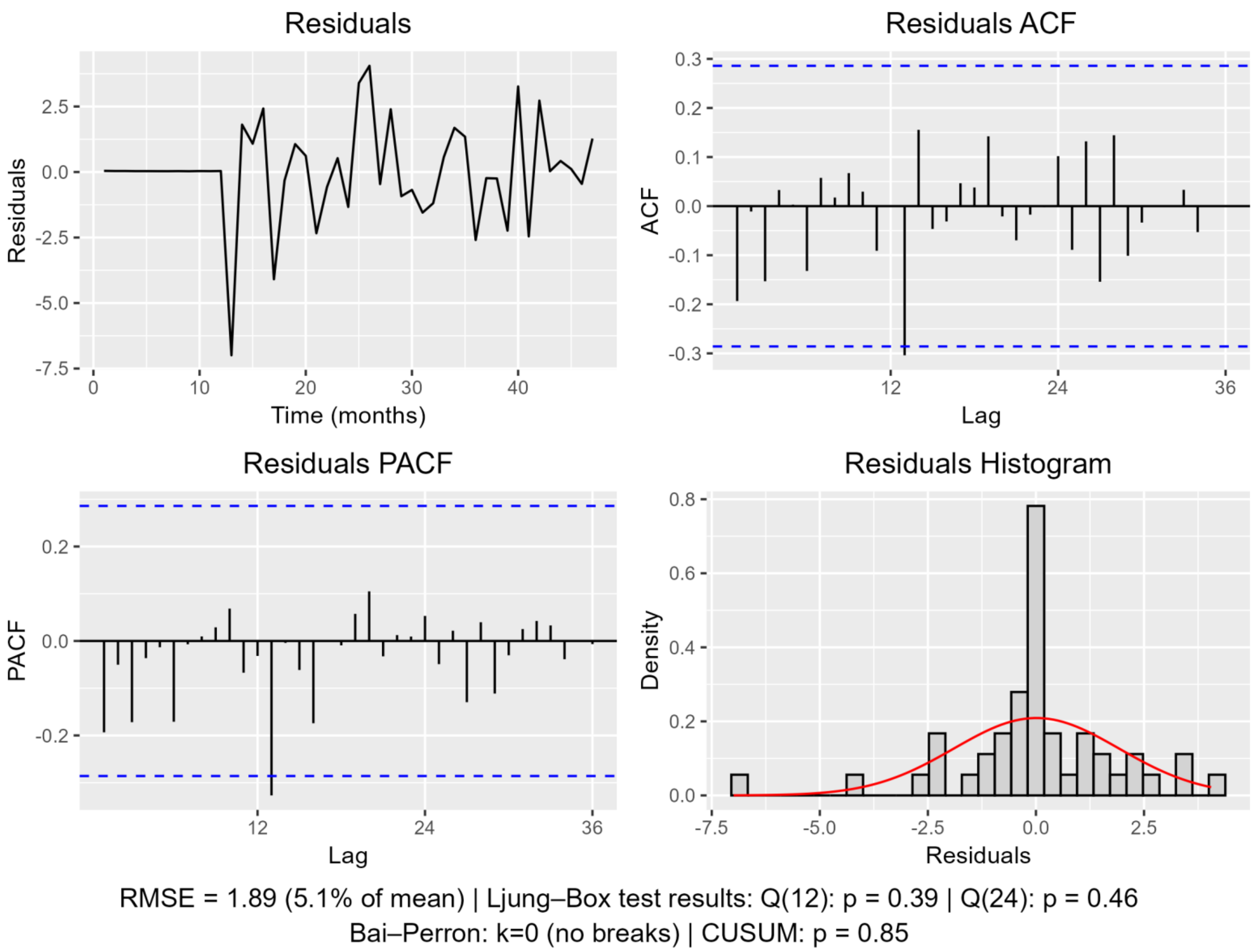

Residual diagnostics are shown for ARIMA(4,0,1)(0,1,1)[12] with drift, including plots of residuals over time (top left), autocorrelation (ACF) plots of residuals (top right), partial autocorrelation (PACF) plots of residuals (bottom left), and residual histograms (bottom right). The root mean squared error (RMSE) for the model is shown in absolute terms and as a percentage of the series mean (normalised RMSE). Ljung–Box test results at 12 and 24 lags are shown, assessing for residual autocorrelation. Bai-Perron tests were used to detect structural breaks in pre-pandemic time series, with the optimal number of breaks (k) determined based upon the Bayesian Information Criterion. Cumulative Sum (CUSUM) tests were used to assess parameter stability over time.

Supplementary Figure S19. Model validation and residual diagnostics for the SARIMA model utilised for epilepsy.

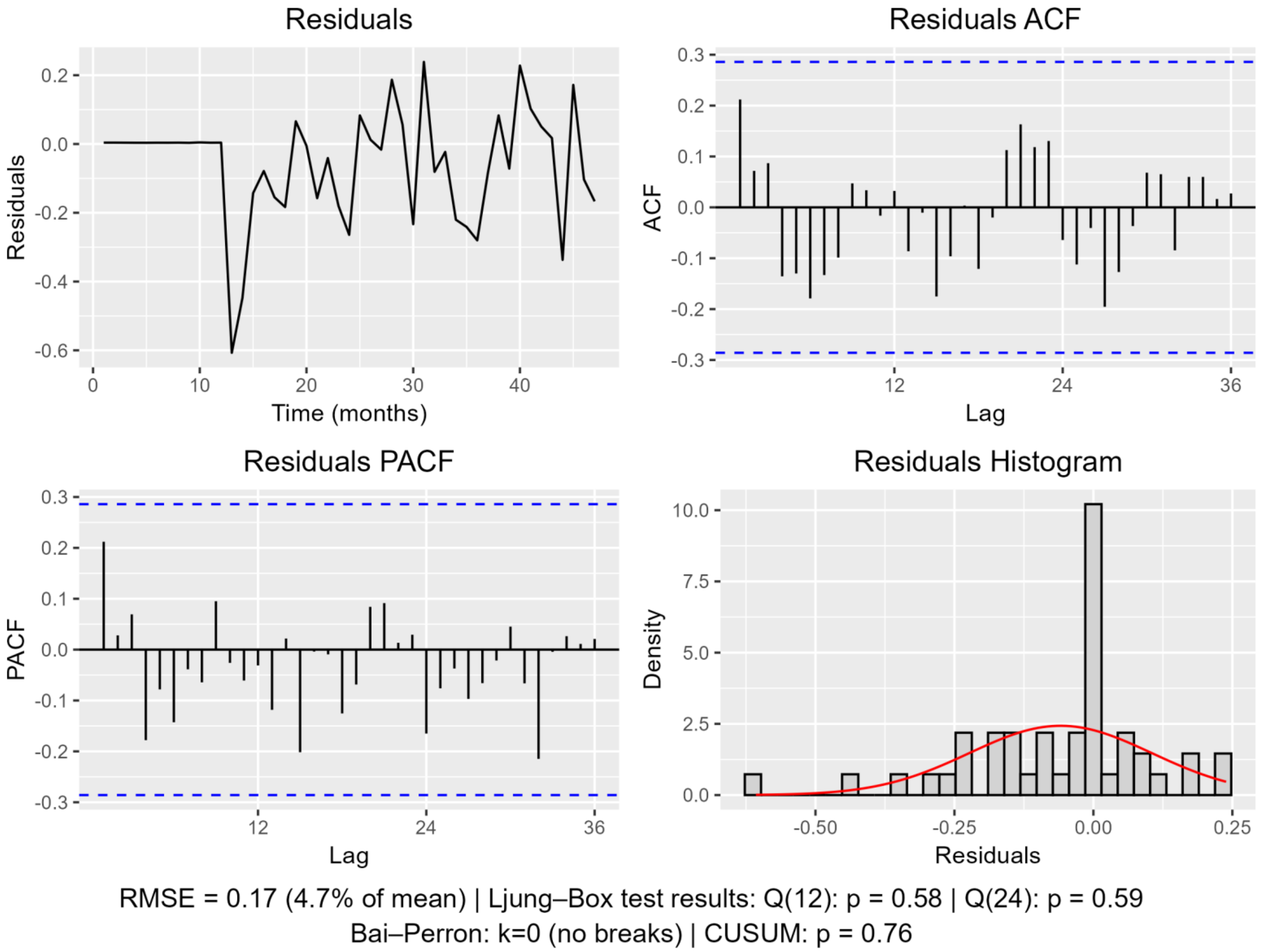

Residual diagnostics are shown for ARIMA(0,0,0)(1,1,1)[12], including plots of residuals over time (top left), autocorrelation (ACF) plots of residuals (top right), partial autocorrelation (PACF) plots of residuals (bottom left), and residual histograms (bottom right). The root mean squared error (RMSE) for the model is shown in absolute terms and as a percentage of the series mean (normalised RMSE). Ljung–Box test results at 12 and 24 lags are shown, assessing for residual autocorrelation. Bai-Perron tests were used to detect structural breaks in pre-pandemic time series, with the optimal number of breaks (k) determined based upon the Bayesian Information Criterion. Cumulative Sum (CUSUM) tests were used to assess parameter stability over time.

Supplementary Figure S20. Model validation and residual diagnostics for the SARIMA model utilised for heart failure.

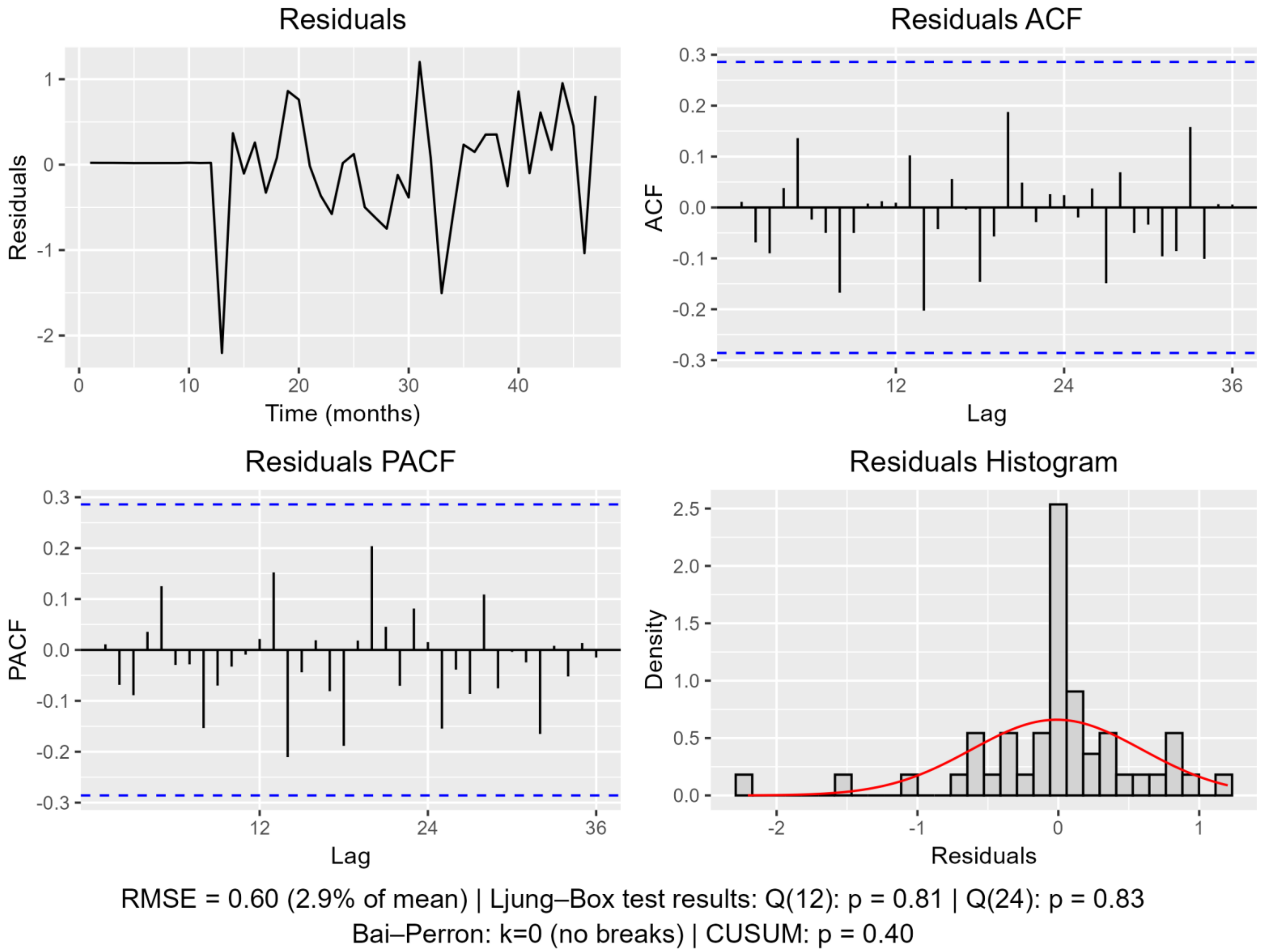

Residual diagnostics are shown for ARIMA(3,0,0)(0,1,1)[12] with drift, including plots of residuals over time (top left), autocorrelation (ACF) plots of residuals (top right), partial autocorrelation (PACF) plots of residuals (bottom left), and residual histograms (bottom right). The root mean squared error (RMSE) for the model is shown in absolute terms and as a percentage of the series mean (normalised RMSE). Ljung–Box test results at 12 and 24 lags are shown, assessing for residual autocorrelation. Bai-Perron tests were used to detect structural breaks in pre-pandemic time series, with the optimal number of breaks (k) determined based upon the Bayesian Information Criterion. Cumulative Sum (CUSUM) tests were used to assess parameter stability over time.

Supplementary Figure S21. Model validation and residual diagnostics for the SARIMA model utilised for multiple sclerosis.

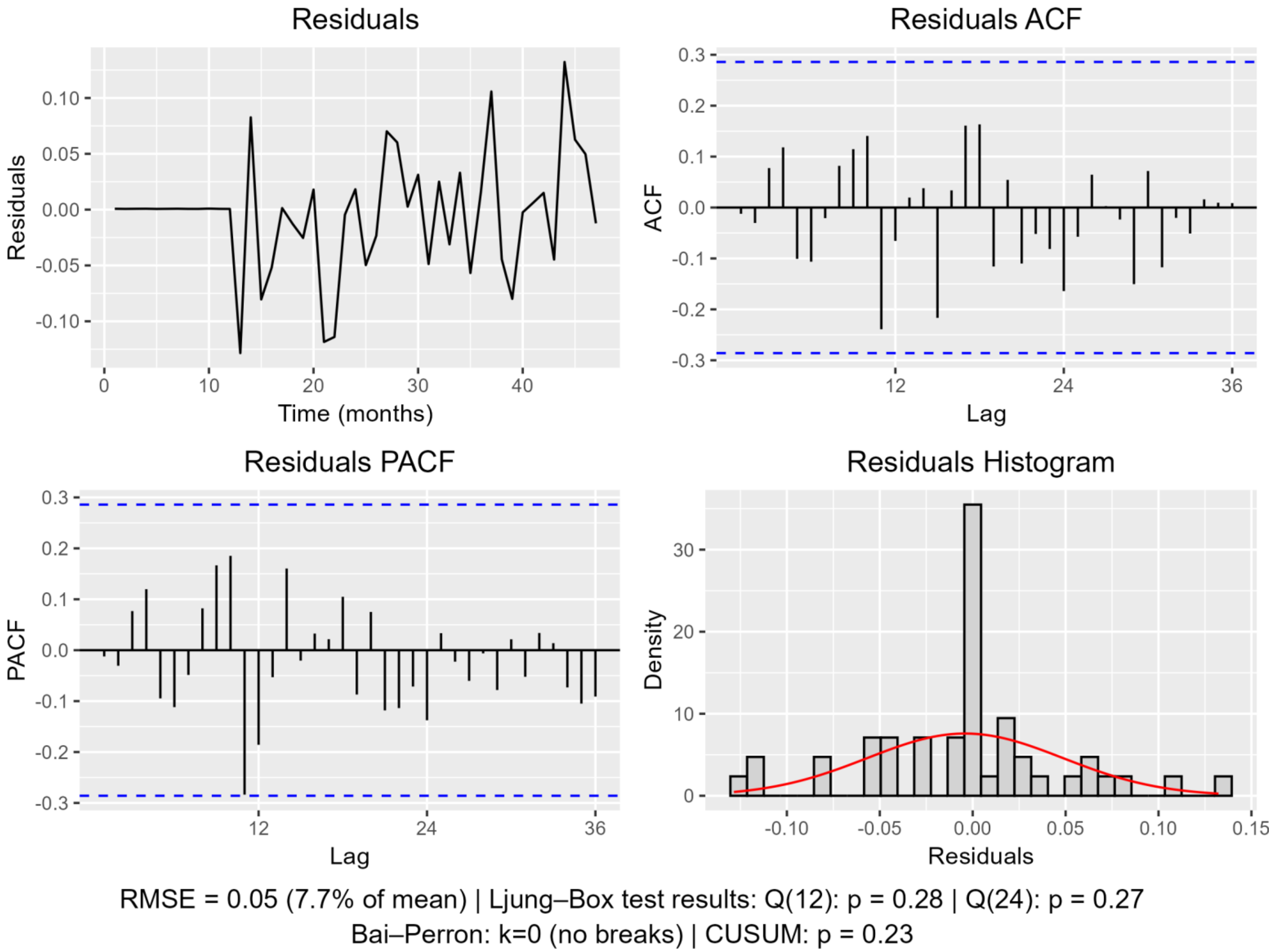

Residual diagnostics are shown for ARIMA(4,0,0)(1,1,0)[12], including plots of residuals over time (top left), autocorrelation (ACF) plots of residuals (top right), partial autocorrelation (PACF) plots of residuals (bottom left), and residual histograms (bottom right). The root mean squared error (RMSE) for the model is shown in absolute terms and as a percentage of the series mean (normalised RMSE). Ljung–Box test results at 12 and 24 lags are shown, assessing for residual autocorrelation. Bai-Perron tests were used to detect structural breaks in pre-pandemic time series, with the optimal number of breaks (k) determined based upon the Bayesian Information Criterion. Cumulative Sum (CUSUM) tests were used to assess parameter stability over time.

Supplementary Figure S22. Model validation and residual diagnostics for the SARIMA model utilised for osteoporosis.

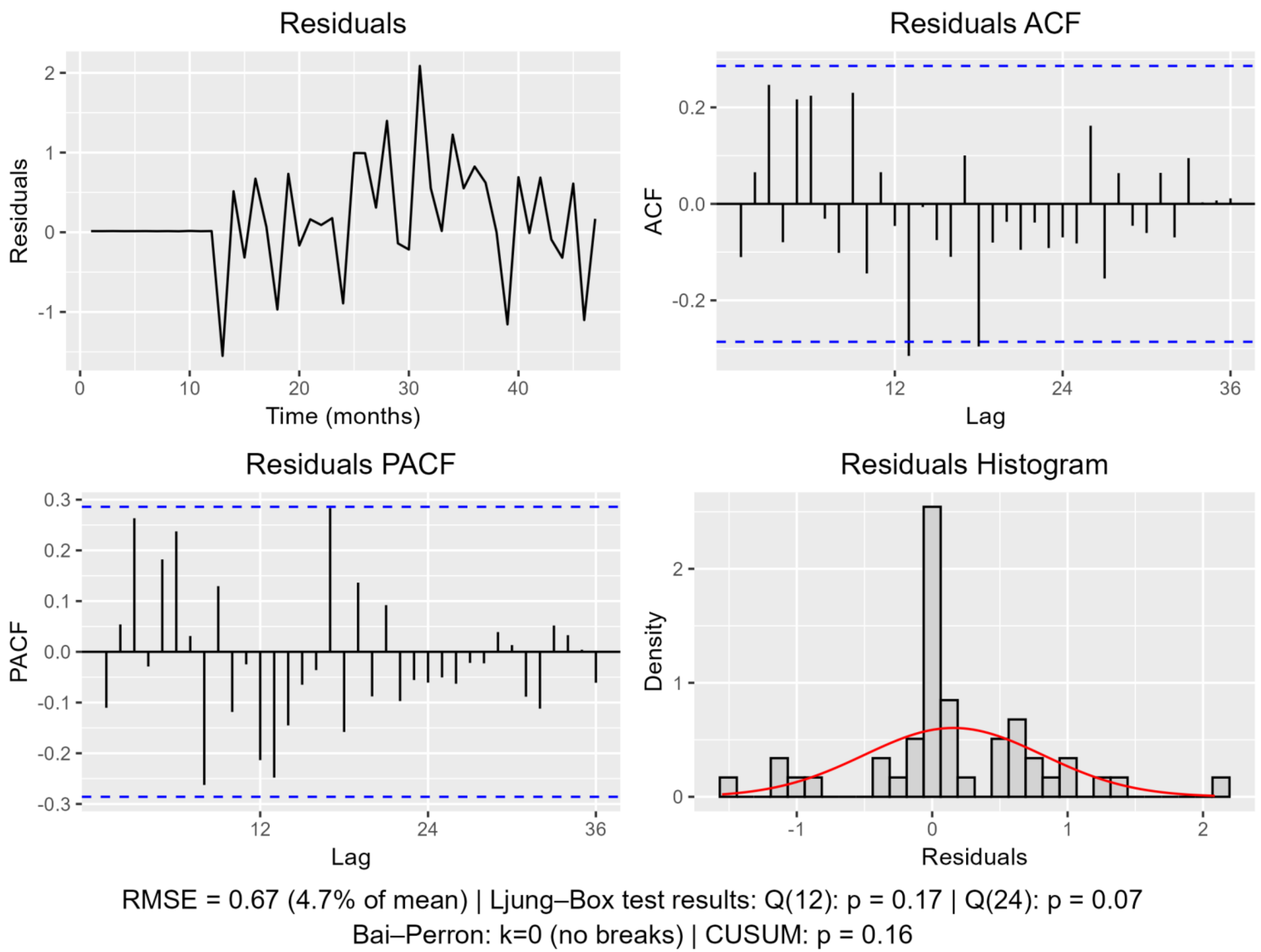

Residual diagnostics are shown for ARIMA(0,0,0)(0,1,1)[12], including plots of residuals over time (top left), autocorrelation (ACF) plots of residuals (top right), partial autocorrelation (PACF) plots of residuals (bottom left), and residual histograms (bottom right). The root mean squared error (RMSE) for the model is shown in absolute terms and as a percentage of the series mean (normalised RMSE). Ljung–Box test results at 12 and 24 lags are shown, assessing for residual autocorrelation. Bai-Perron tests were used to detect structural breaks in pre-pandemic time series, with the optimal number of breaks (k) determined based upon the Bayesian Information Criterion. Cumulative Sum (CUSUM) tests were used to assess parameter stability over time.

Supplementary Figure S23. Model validation and residual diagnostics for the SARIMA model utilised for polymyalgia rheumatica.

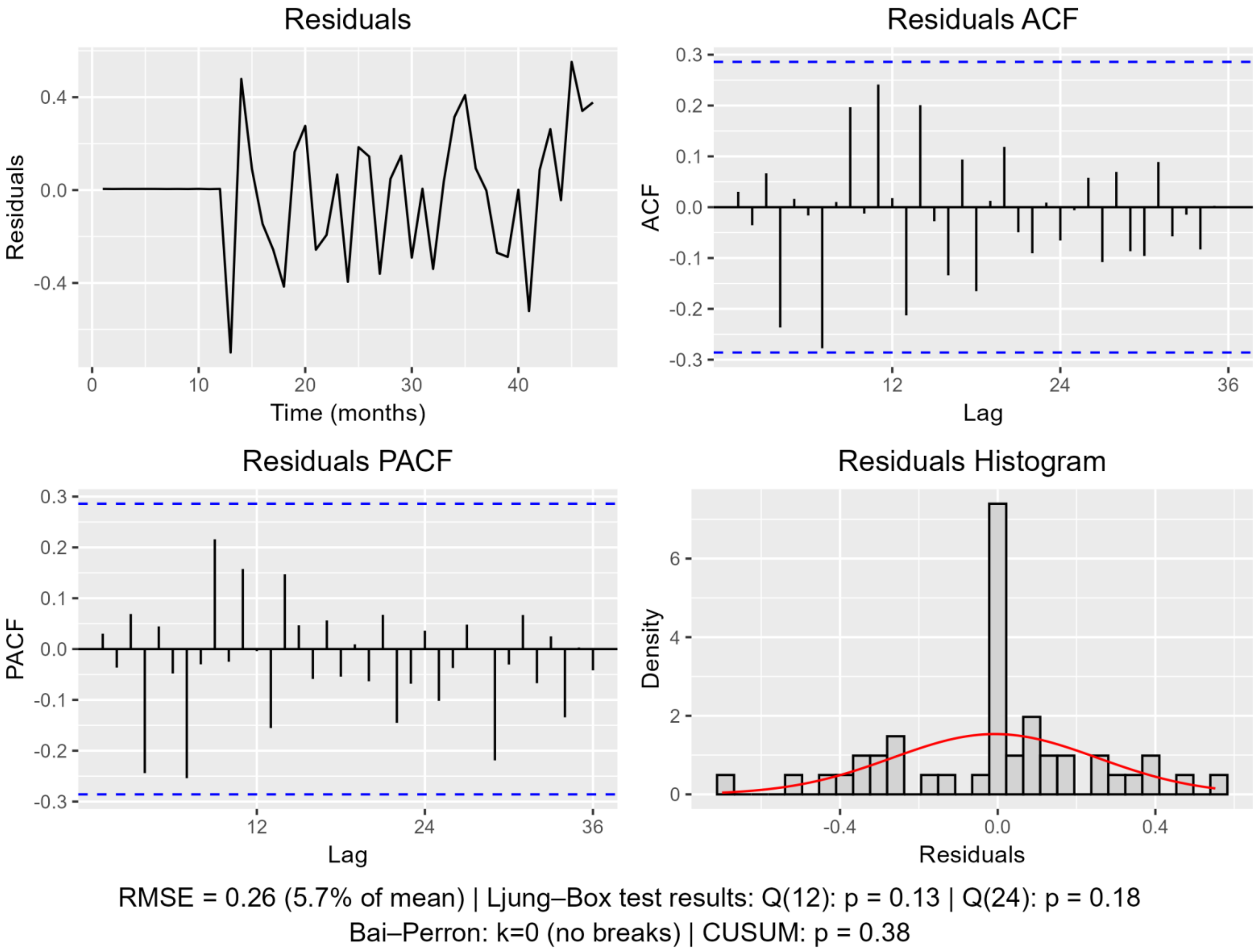

Residual diagnostics are shown for ARIMA(0,0,1)(1,1,0)[12] with drift, including plots of residuals over time (top left), autocorrelation (ACF) plots of residuals (top right), partial autocorrelation (PACF) plots of residuals (bottom left), and residual histograms (bottom right). The root mean squared error (RMSE) for the model is shown in absolute terms and as a percentage of the series mean (normalised RMSE). Ljung–Box test results at 12 and 24 lags are shown, assessing for residual autocorrelation. Bai-Perron tests were used to detect structural breaks in pre-pandemic time series, with the optimal number of breaks (k) determined based upon the Bayesian Information Criterion. Cumulative Sum (CUSUM) tests were used to assess parameter stability over time.

Supplementary Figure S24. Model validation and residual diagnostics for the SARIMA model utilised for psoriasis.

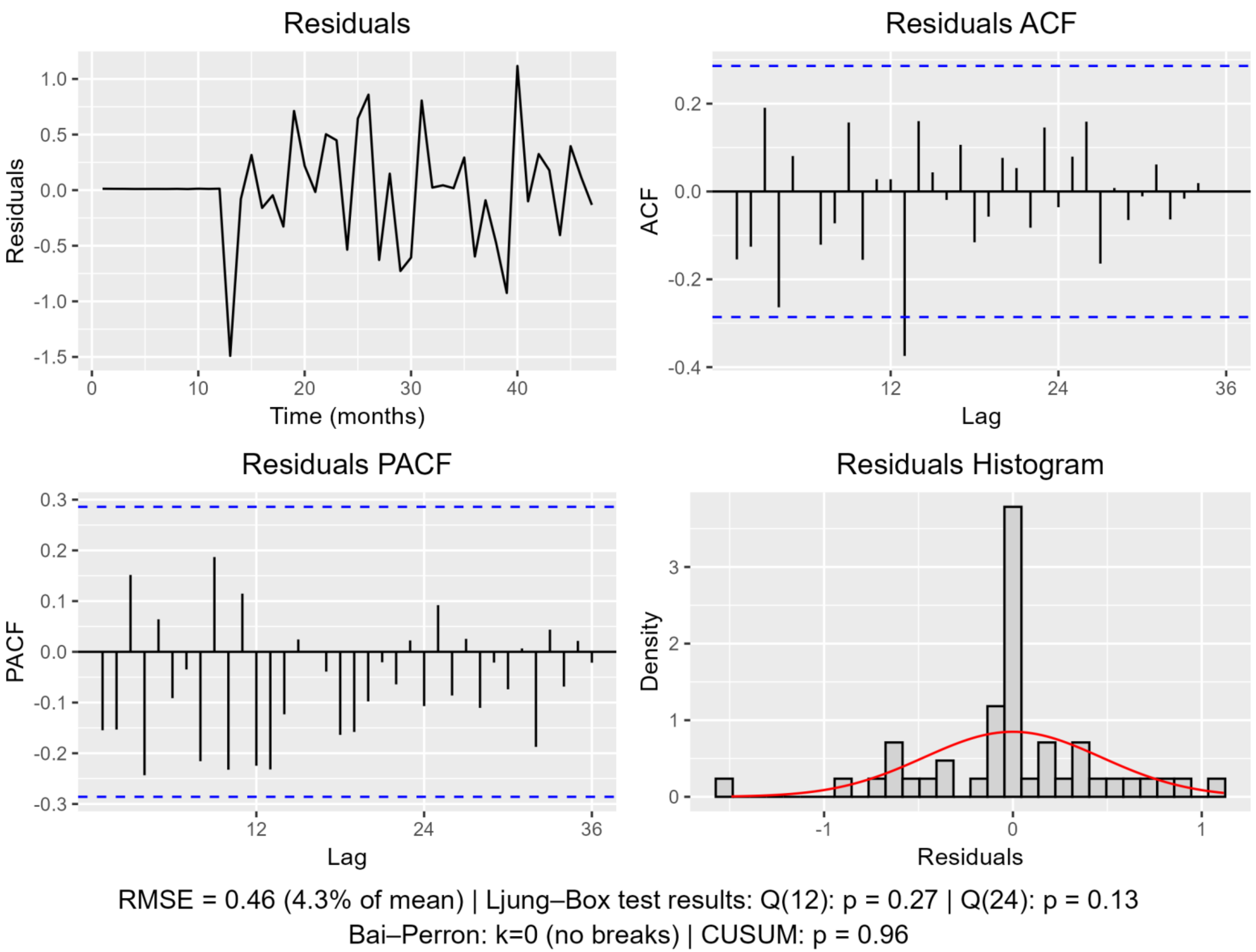

Residual diagnostics are shown for ARIMA(0,0,0)(0,1,1)[12] with drift, including plots of residuals over time (top left), autocorrelation (ACF) plots of residuals (top right), partial autocorrelation (PACF) plots of residuals (bottom left), and residual histograms (bottom right). The root mean squared error (RMSE) for the model is shown in absolute terms and as a percentage of the series mean (normalised RMSE). Ljung–Box test results at 12 and 24 lags are shown, assessing for residual autocorrelation. Bai-Perron tests were used to detect structural breaks in pre-pandemic time series, with the optimal number of breaks (k) determined based upon the Bayesian Information Criterion. Cumulative Sum (CUSUM) tests were used to assess parameter stability over time.

Supplementary Figure S25. Model validation and residual diagnostics for the SARIMA model utilised for rheumatoid arthritis.

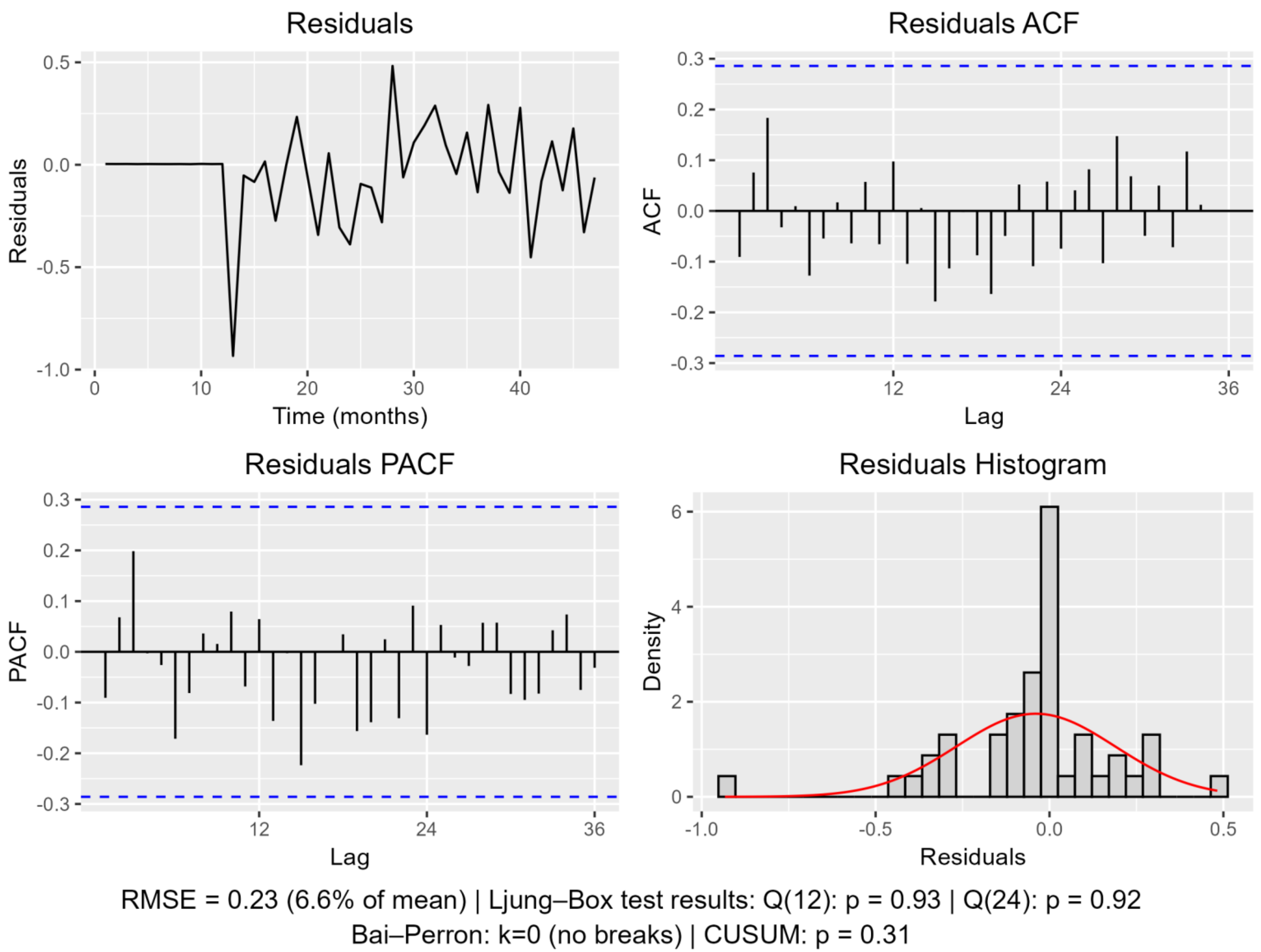

Residual diagnostics are shown for ARIMA(0,0,0)(0,1,1)[12], including plots of residuals over time (top left), autocorrelation (ACF) plots of residuals (top right), partial autocorrelation (PACF) plots of residuals (bottom left), and residual histograms (bottom right). The root mean squared error (RMSE) for the model is shown in absolute terms and as a percentage of the series mean (normalised RMSE). Ljung–Box test results at 12 and 24 lags are shown, assessing for residual autocorrelation. Bai-Perron tests were used to detect structural breaks in pre-pandemic time series, with the optimal number of breaks (k) determined based upon the Bayesian Information Criterion. Cumulative Sum (CUSUM) tests were used to assess parameter stability over time.

Supplementary Figure S26. Model validation and residual diagnostics for the SARIMA model utilised for stroke and transient ischaemic attacks.

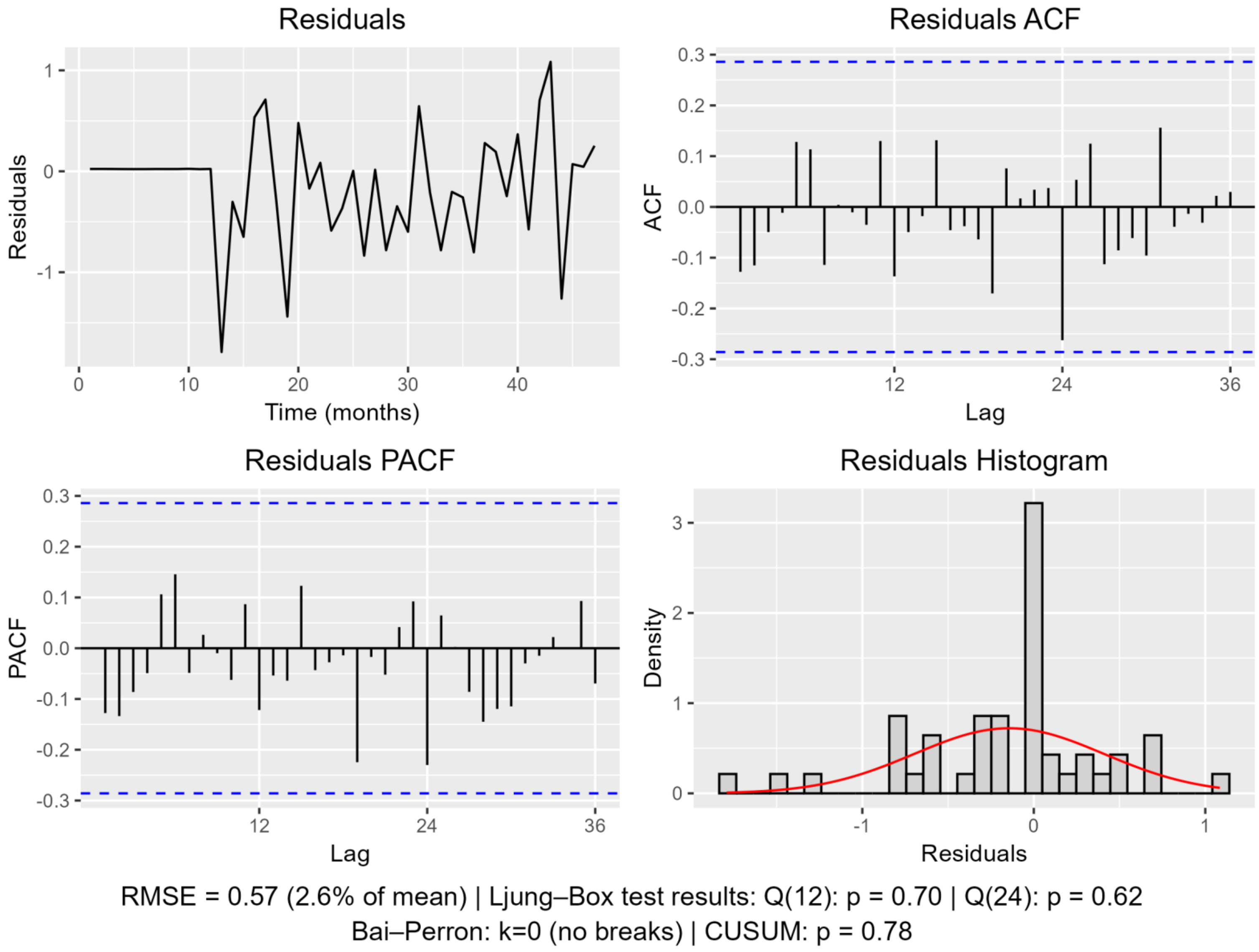

Residual diagnostics are shown for ARIMA(0,0,2)(1,1,0)[12], including plots of residuals over time (top left), autocorrelation (ACF) plots of residuals (top right), partial autocorrelation (PACF) plots of residuals (bottom left), and residual histograms (bottom right). The root mean squared error (RMSE) for the model is shown in absolute terms and as a percentage of the series mean (normalised RMSE). Ljung–Box test results at 12 and 24 lags are shown, assessing for residual autocorrelation. Bai-Perron tests were used to detect structural breaks in pre-pandemic time series, with the optimal number of breaks (k) determined based upon the Bayesian Information Criterion. Cumulative Sum (CUSUM) tests were used to assess parameter stability over time.

Supplementary Figure S27. Model validation and residual diagnostics for the SARIMA model utilised for ulcerative colitis.

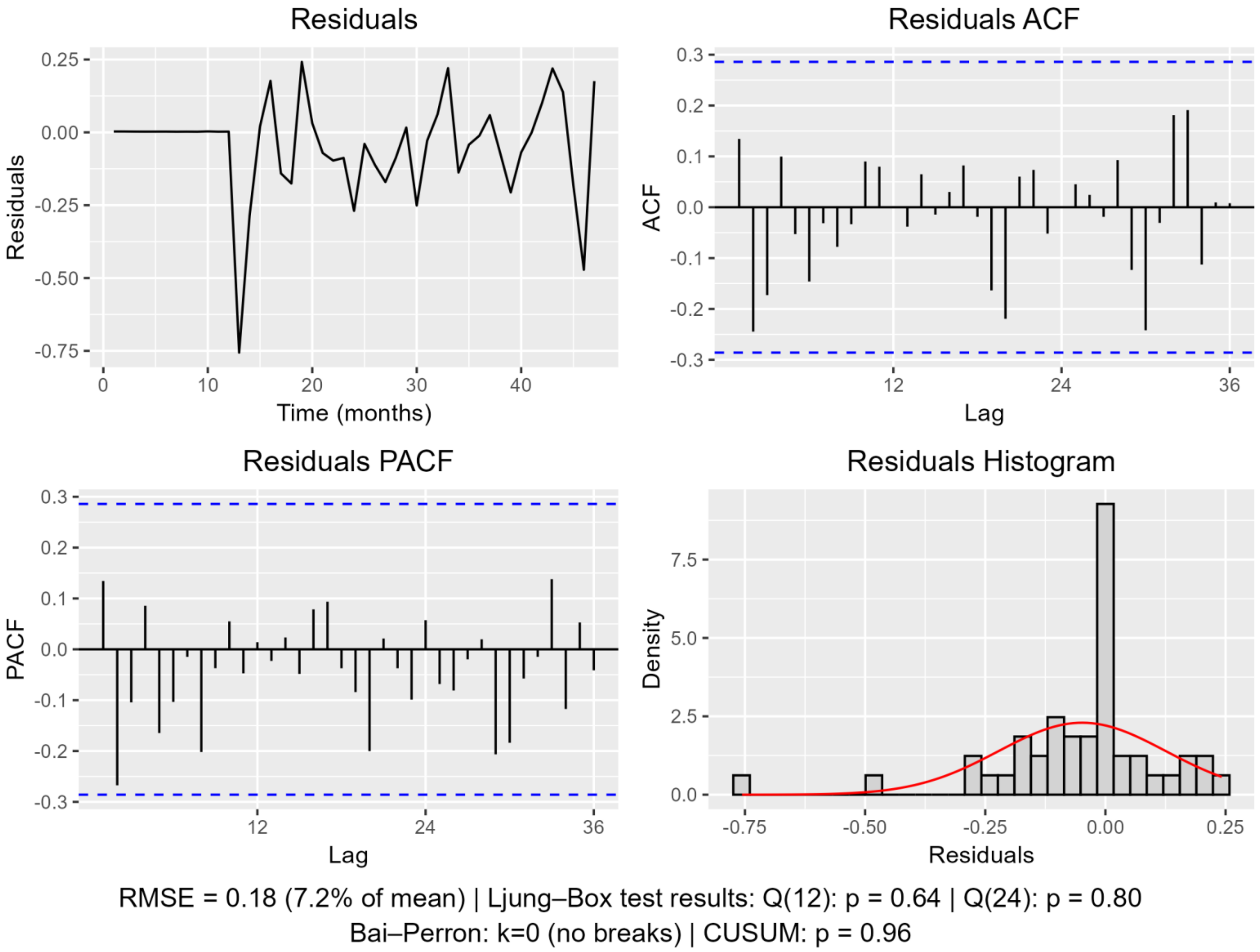

Residual diagnostics are shown for ARIMA(0,0,0)(0,1,1)[12], including plots of residuals over time (top left), autocorrelation (ACF) plots of residuals (top right), partial autocorrelation (PACF) plots of residuals (bottom left), and residual histograms (bottom right). The root mean squared error (RMSE) for the model is shown in absolute terms and as a percentage of the series mean (normalised RMSE). Ljung–Box test results at 12 and 24 lags are shown, assessing for residual autocorrelation. Bai-Perron tests were used to detect structural breaks in pre-pandemic time series, with the optimal number of breaks (k) determined based upon the Bayesian Information Criterion. Cumulative Sum (CUSUM) tests were used to assess parameter stability over time.

Supplementary Table S1. Sociodemographic characteristics for the reference population and for individuals with newly recorded diagnoses of 19 long-term health conditions.

	Reference population n=29,995,025	Asthma n=495,260	Atopic dermatitis n=100,815	Coronary heart disease n=485,565	Chronic kidney disease n=720,530	Coeliac disease n=47,535	COPD n=373,435	Crohn's disease n=37,555	Dementia n=371,675	Depression n=1,246,100
Mean (SD) age, years	42.2 (22.9)	35.9 (24.6)	34.7 (25.7)	67.3 (12.8)	74.2 (12.1)	39.1 (22.1)	66.6 (12.3)	42.6 (19.6)	81.9 (8.2)	38.9 (18.1)
Sex										
Female	15,053,705 (50.2%)	274,005 (55.3%)	56,750 (56.3%)	182,680 (37.6%)	374,475 (52.0%)	31,075 (65.4%)	178,020 (47.7%)	18,955 (50.5%)	219,675 (59.1%)	709,005 (56.9%)
Male	14,941,320 (49.8%)	221,255 (44.7%)	44,065 (43.7%)	302,880 (62.4%)	346,060 (48.0%)	16,460 (34.6%)	195,415 (52.3%)	18,600 (49.5%)	152,000 (40.9%)	537,100 (43.1%)
Ethnicity										
White	23,113,760 (77.0%)	407,670 (82.3%)	77,870 (77.2%)	437,110 (90.0%)	659,890 (91.6%)	41,810 (87.9%)	357,510 (95.7%)	33,210 (88.4%)	351,690 (94.6%)	1,086,600 (87.2%)
Asian/Asian British	2,674,435 (8.9%)	45,420 (9.2%)	12,545 (12.4%)	31,845 (6.6%)	30,635 (4.3%)	4,080 (8.6%)	6,670 (1.8%)	2,570 (6.8%)	9,020 (2.4%)	66,085 (5.3%)
Black/Black British	1,011,970 (3.4%)	11,890 (2.4%)	3,060 (3.0%)	5,725 (1.2%)	13,755 (1.9%)	280 (0.6%)	2,320 (0.6%)	470 (1.3%)	3,740 (1.0%)	22,600 (1.8%)
Mixed	556,300 (1.9%)	11,075 (2.2%)	2,415 (2.4%)	2,815 (0.6%)	3,950 (0.5%)	450 (0.9%)	1,405 (0.4%)	555 (1.5%)	1,260 (0.3%)	20,400 (1.6%)
Chinese or other ethnicity	948,400 (3.2%)	8,545 (1.7%)	2,200 (2.2%)	4,975 (1.0%)	4,755 (0.7%)	445 (0.9%)	1,960 (0.5%)	500 (1.3%)	1,835 (0.5%)	18,755 (1.5%)
Unknown	1,690,165 (5.7%)	10,665 (2.2%)	2,720 (2.7%)	3,095 (0.6%)	7,540 (1.0%)	475 (1.0%)	3,570 (1.0%)	250 (0.7%)	4,135 (1.1%)	31,665 (2.5%)
Index of multiple deprivation										
1 most deprived	5,835,515 (19.5%)	108,705 (21.9%)	21,745 (21.6%)	89,665 (18.5%)	122,155 (17.0%)	7,830 (16.5%)	103,415 (27.7%)	7,200 (19.2%)	63,250 (17.0%)	296,470 (23.8%)
2	5,866,365 (19.6%)	96,160 (19.4%)	19,190 (19.0%)	92,820 (19.1%)	133,690 (18.6%)	8,060 (17.0%)	80,810 (21.6%)	7,170 (19.1%)	69,375 (18.7%)	253,285 (20.3%)
3	6,233,035 (20.8%)	99,180 (20.0%)	20,135 (20.0%)	105,460 (21.7%)	158,980 (22.1%)	9,715 (20.4%)	75,515 (20.2%)	7,750 (20.6%)	82,705 (22.3%)	245,600 (19.7%)
4	5,725,365 (19.1%)	89,470 (18.1%)	18,455 (18.3%)	97,650 (20.1%)	151,580 (21.0%)	9,975 (21.0%)	60,700 (16.3%)	7,340 (19.5%)	78,425 (21.1%)	215,855 (17.3%)
5 least deprived	5,192,805 (17.3%)	82,795 (16.7%)	17,680 (17.5%)	89,610 (18.5%)	139,365 (19.3%)	10,110 (21.3%)	45,585 (12.2%)	6,665 (17.7%)	71,820 (19.3%)	183,980 (14.8%)
Unknown	1,141,940 (3.8%)	18,950 (3.8%)	3,610 (3.6%)	10,355 (2.1%)	14,765 (2.0%)	1,850 (3.9%)	7,410 (2.0%)	1,430 (3.8%)	6,100 (1.6%)	50,910 (4.1%)

Supplementary Table S1 (continued). Sociodemographic characteristics for the reference population and for individuals with newly recorded diagnoses of 19 long-term health conditions.

	Diabetes mellitus type 2 n=822,735	Epilepsy n=76,880	Heart failure n=469,390	Multiple sclerosis n=15,155	Osteoporosis n=298,265	Polymyalgia rheumatica n=94,340	Psoriasis n=209,290	Rheumatoid arthritis n=75,895	Stroke/TIA n=469,075	Ulcerative colitis n=58,490
Mean (SD) age, years	60.2 (14.5)	42.8 (26.3)	75.5 (13.1)	43.7 (14.0)	72.3 (12.2)	72.5 (9.8)	45.6 (20.5)	59.5 (16.3)	72.2 (14.1)	47.3 (19.4)
Sex										
Female	367,810 (44.7%)	35,720 (46.5%)	209,765 (44.7%)	10,375 (68.5%)	239,525 (80.3%)	56,100 (59.5%)	111,475 (53.3%)	50,045 (65.9%)	228,190 (48.6%)	28,790 (49.2%)
Male	454,925 (55.3%)	41,160 (53.5%)	259,625 (55.3%)	4,780 (31.5%)	58,740 (19.7%)	38,240 (40.5%)	97,810 (46.7%)	25,850 (34.1%)	240,885 (51.4%)	29,700 (50.8%)
Ethnicity										
White	670,225 (81.5%)	67,670 (88.0%)	439,390 (93.6%)	13,520 (89.2%)	281,570 (94.4%)	90,770 (96.2%)	181,330 (86.6%)	66,890 (88.1%)	434,490 (92.6%)	51,985 (88.9%)
Asian/Asian British	93,570 (11.4%)	4,585 (6.0%)	16,730 (3.6%)	740 (4.9%)	9,565 (3.2%)	1,550 (1.6%)	15,275 (7.3%)	5,710 (7.5%)	18,270 (3.9%)	3,955 (6.8%)
Black/Black British	25,580 (3.1%)	1,825 (2.4%)	5,220 (1.1%)	370 (2.4%)	1,745 (0.6%)	535 (0.6%)	2,260 (1.1%)	1,355 (1.8%)	6,380 (1.4%)	705 (1.2%)
Mixed	8,415 (1.0%)	1,270 (1.7%)	1,895 (0.4%)	225 (1.5%)	1,050 (0.4%)	255 (0.3%)	2,495 (1.2%)	665 (0.9%)	2,360 (0.5%)	690 (1.2%)
Chinese or other ethnicity	12,295 (1.5%)	965 (1.3%)	2,805 (0.6%)	220 (1.5%)	2,440 (0.8%)	355 (0.4%)	3,150 (1.5%)	820 (1.1%)	3,460 (0.7%)	685 (1.2%)
Unknown	12,650 (1.5%)	565 (0.7%)	3,350 (0.7%)	75 (0.5%)	1,900 (0.6%)	870 (0.9%)	4,775 (2.3%)	455 (0.6%)	4,115 (0.9%)	470 (0.8%)
Index of multiple deprivation										
1 most deprived	191,135 (23.2%)	19,045 (24.8%)	86,670 (18.5%)	2,575 (17.0%)	45,920 (15.4%)	9,775 (10.4%)	42,930 (20.5%)	14,125 (18.6%)	83,355 (17.8%)	9,890 (16.9%)
2	173,025 (21.0%)	15,505 (20.2%)	89,925 (19.2%)	2,920 (19.3%)	52,010 (17.4%)	14,695 (15.6%)	40,185 (19.2%)	14,740 (19.4%)	88,000 (18.8%)	10,875 (18.6%)
3	171,195 (20.8%)	15,125 (19.7%)	102,785 (21.9%)	3,135 (20.7%)	65,305 (21.9%)	22,135 (23.5%)	42,715 (20.4%)	16,560 (21.8%)	102,095 (21.8%)	12,325 (21.1%)
4	146,315 (17.8%)	12,975 (16.9%)	95,890 (20.4%)	3,045 (20.1%)	64,965 (21.8%)	22,780 (24.1%)	39,830 (19.0%)	14,960 (19.7%)	96,855 (20.6%)	11,885 (20.3%)
5 least deprived	120,620 (14.7%)	11,835 (15.4%)	85,715 (18.3%)	2,855 (18.8%)	63,465 (21.3%)	23,205 (24.6%)	36,415 (17.4%)	13,480 (17.8%)	89,600 (19.1%)	11,285 (19.3%)
Unknown	20,440 (2.5%)	2,400 (3.1%)	8,410 (1.8%)	620 (4.1%)	6,605 (2.2%)	1,750 (1.9%)	7,215 (3.4%)	2,025 (2.7%)	9,170 (2.0%)	2,230 (3.8%)

Sociodemographic characteristics are summarised for the reference population (individuals registered with general practices at any point during the study period, with mean age shown at the study midpoint, August 2020), and at the time of diagnosis for individuals from the reference population who had newly recorded diagnoses of one or more of 19 long-term conditions during the study period. Counts have been rounded to the nearest 5, to reduce the risk of disclosure. As such, column totals may differ from the sum of the individual variables. COPD: chronic obstructive pulmonary disease; TIA: transient ischaemic attack.

Supplementary Table S2. Yearly differences between expected and observed diagnosis rates for 19 long-term conditions after the onset of the COVID-19 pandemic in England.

Condition	March 2020 to February 2021		March 2021 to February 2022		March 2022 to February 2023		March 2023 to November 2024	
	Absolute difference between expected and observed diagnoses per 100,000 population (95% PI)	Relative difference between expected and observed diagnoses per 100,000 population (95% PI)	Absolute difference between expected and observed diagnoses per 100,000 population (95% PI)	Relative difference between expected and observed diagnoses per 100,000 population (95% PI)	Absolute difference between expected and observed diagnoses per 100,000 population (95% PI)	Relative difference between expected and observed diagnoses per 100,000 population (95% PI)	Absolute difference between expected and observed diagnoses per 100,000 population (95% PI)	Relative difference between expected and observed diagnoses per 100,000 population (95% PI)
Asthma	-109<br>(-119 to -99.0)	-31.5%<br>(-33.5 to -29.4)	-67.2<br>(-79.3 to -55.2)	-19.4%<br>(-22.1 to -16.5)	-21.3<br>(-33.8 to -8.7)	-6.1%<br>(-9.4 to -2.6)	-70.5<br>(-88.3 to -52.6)	-11.7%<br>(-14.3 to -9.0)
Atopic Dermatitis	-25.1<br>(-29.4 to -20.8)	-38.6%<br>(-42.4 to -34.3)	-8.4<br>(-14.6 to -2.2)	-14.5%<br>(-22.8 to -4.2)	3.6<br>(-5.2 to 12.4)	7.2%<br>(-8.8 to 30.0)	48.2<br>(30.7 to 65.7)	69.5%<br>(35.3 to 127)
Coronary Heart Disease	-54.0<br>(-58.1 to -49.8)	-18.4%<br>(-19.6 to -17.3)	-20.0<br>(-24.7 to -15.4)	-6.9%<br>(-8.4 to -5.4)	-13.2<br>(-18.2 to -8.3)	-4.6%<br>(-6.2 to -2.9)	-12.5<br>(-19.6 to -5.4)	-2.5%<br>(-3.9 to -1.1)
Chronic Kidney Disease	-96.0<br>(-111 to -80.8)	-25.3%<br>(-28.2 to -22.2)	23.9<br>(2.7 to 45.1)	6.2%<br>(0.7 to 12.5)	192<br>(171 to 214)	50.2%<br>(42.2 to 59.2)	509<br>(480 to 538)	76.4%<br>(69.0 to 84.4)
Coeliac Disease	-8.7<br>(-10.0 to -7.5)	-32.9%<br>(-36.0 to -29.6)	-3.9<br>(-5.1 to -2.6)	-14.4%<br>(-18.2 to -10.1)	0.4<br>(-0.9 to 1.7)	1.5%<br>(-3.1 to 6.6)	4.0<br>(2.2 to 5.8)	8.3%<br>(4.4 to 12.5)
COPD	-124<br>(-130 to -119)	-55.7%<br>(-56.8 to -54.6)	-75.8<br>(-82.5 to -69.1)	-35.0%<br>(-36.9 to -32.9)	-18.5<br>(-25.6 to -11.3)	-8.8%<br>(-11.8 to -5.6)	60.6<br>(50.4 to 70.8)	17.4%<br>(14.1 to 21.0)
Crohn's Disease	-2.6<br>(-3.2 to -2.0)	-14.2%<br>(-16.8 to -11.3)	1.6<br>(0.5 to 2.6)	8.9%<br>(2.8 to 15.8)	3.0<br>(1.8 to 4.1)	17.4%<br>(9.7 to 26.3)	6.4<br>(4.7 to 8.1)	22.8%<br>(15.7 to 30.8)
Dementia	-46.2<br>(-52.2 to -40.2)	-22.8%<br>(-25.0 to -20.5)	-10.0<br>(-16.3 to -3.7)	-4.9%<br>(-7.8 to -1.9)	0.7<br>(-6.1 to 7.4)	0.3%<br>(-2.9 to 3.8)	19.4<br>(9.7 to 29.1)	5.5%<br>(2.7 to 8.4)
Depression	-274<br>(-296 to -252)	-29.4%<br>(-31.0 to -27.7)	-173<br>(-198 to -148)	-18.1%<br>(-20.2 to -15.9)	-236<br>(-262 to -210)	-24.0%<br>(-26.0 to -22.0)	-605<br>(-642 to -569)	-34.0%<br>(-35.3 to -32.6)
Diabetes Mellitus Type 2	-111<br>(-130 to -93.0)	-23.3%<br>(-26.1 to -20.2)	-10.4<br>(-30.1 to 9.3)	-2.1%<br>(-5.9 to 2.0)	26.1<br>(6.3 to 45.9)	5.2%<br>(1.2 to 9.5)	81.0<br>(54.8 to 107)	8.9%<br>(5.8 to 12.1)
Epilepsy	-5.2<br>(-6.3 to -4.0)	-11.8%<br>(-14.0 to -9.3)	-2.4<br>(-3.6 to -1.2)	-5.5%<br>(-8.0 to -2.8)	-3.2<br>(-4.5 to -2.0)	-7.4%<br>(-9.9 to -4.7)	-2.2<br>(-3.9 to -0.6)	-2.9%<br>(-5.0 to -0.8)
Heart Failure	-51.0<br>(-56.0 to -46.1)	-19.5%<br>(-21.0 to -18.0)	-3.8<br>(-8.9 to 1.3)	-1.4%<br>(-3.3 to 0.5)	3.5<br>(-1.6 to 8.6)	1.3%<br>(-0.6 to 3.2)	12.6<br>(5.8 to 19.3)	2.6%<br>(1.2 to 4.0)
Multiple Sclerosis	-0.9<br>(-1.4 to -0.5)	-11.4%<br>(-15.7 to -6.5)	-0.2<br>(-0.6 to 0.3)	-2.0%<br>(-7.1 to 3.9)	-0.6<br>(-1.1 to 0)	-6.8%<br>(-12.5 to -0.3)	-1.1<br>(-1.9 to -0.3)	-7.8%<br>(-12.7 to -2.4)
Osteoporosis	-61.1<br>(-65.5 to -56.7)	-35.3%<br>(-36.9 to -33.6)	-23.3<br>(-28.6 to -18.0)	-13.5%<br>(-16.0 to -10.7)	-8.3<br>(-14.0 to -2.5)	-4.8%<br>(-7.8 to -1.5)	-2.3<br>(-10.6 to 6.0)	-0.8%<br>(-3.4 to 2.1)
Polymyalgia Rheumatica	-3.7<br>(-5.6 to -1.9)	-7.4%<br>(-10.7 to -3.9)	-1.7<br>(-4.0 to 0.6)	-3.5%<br>(-7.8 to 1.3)	-3.4<br>(-6.1 to -0.7)	-7.2%<br>(-12.3 to -1.6)	1.8<br>(-2.4 to 6.0)	2.3%<br>(-3.0 to 8.2)
Psoriasis	-53.8<br>(-56.9 to -50.7)	-43.6%<br>(-44.9 to -42.1)	-29.3<br>(-32.9 to -25.6)	-24.3%<br>(-26.5 to -21.9)	-11.4<br>(-15.2 to -7.7)	-9.7%<br>(-12.5 to -6.7)	-1.4<br>(-6.6 to 3.9)	-0.7%<br>(-3.2 to 2.0)
Rheumatoid Arthritis	-10.1<br>(-11.4 to -8.8)	-24.3%<br>(-26.6 to -21.9)	-4.5<br>(-6.2 to -2.8)	-10.8%<br>(-14.2 to -7.0)	-3.6<br>(-5.4 to -1.9)	-8.7%<br>(-12.5 to -4.7)	-1.1<br>(-3.7 to 1.5)	-1.5%<br>(-4.8 to 2.1)
Stroke/TIA	-24.2<br>(-28.7 to -19.8)	-9.3%<br>(-10.8 to -7.7)	-0.8<br>(-5.8 to 4.2)	-0.3%<br>(-2.2 to 1.7)	-0.9<br>(-6.8 to 5.0)	-0.3%<br>(-2.5 to 2.0)	2.0<br>(-7.1 to 11.0)	0.4%<br>(-1.5 to 2.5)
Ulcerative Colitis	-4.0<br>(-5.2 to -2.8)	-13.5%<br>(-16.8 to -9.8)	0.2<br>(-1.3 to 1.6)	0.5%<br>(-4.2 to 5.7)	1.8<br>(0.2 to 3.4)	6.2%<br>(0.8 to 12.2)	5.5<br>(3.2 to 7.7)	10.5%<br>(5.9 to 15.6)

Absolute and relative differences between expected and observed rates of new diagnoses per 100,000 population for 19 long-term conditions, shown separately for time periods after the onset of the COVID-19 pandemic in England. Expected diagnoses rates were modelled using seasonal autoregressive integrated moving averages (SARIMA), utilising data from April, 2016, to February, 2020. COPD: chronic obstructive pulmonary disease; TIA: transient ischaemic attack; PI: prediction interval.

Supplementary Table S3. Sensitivity analysis using Prophet to forecast differences between expected and observed rates of new diagnoses for 19 long-term conditions in England after the onset of the COVID-19 pandemic.

	**First year of pandemic (March 2020 to February 2021)**			**Cumulative difference for period after the onset of the pandemic (March 2020 to November 2024)**			
**Condition**	Observed diagnosis rate per 100,000 population	Expected diagnosis rate per 100,000 population (95% PI)	Percentage difference between expected and observed (95% PI)	Observed diagnosis rate per 100,000 population	Expected diagnosis rate per 100,000 population (95% PI)	Percentage difference between expected and observed (95% PI)	Absolute difference in diagnoses extrapolated to full population of England (95% PI)
Asthma	237	361<br>(355 to 367)	-34.4%<br>(-35.5 to -33.3)	1,370	1,818<br>(1,802 to 1,835)	-24.7%<br>(-25.3 to -24.0)	-255,600<br>(-264,900 to -246,300)
Atopic Dermatitis	40.0	64.8<br>(62.3 to 67.2)	-38.3%<br>(-40.5 to -35.8)	261	242<br>(236 to 248)	7.8%<br>(5.1 to 10.6)	10,700<br>(7,240 to 14,300)
Coronary Heart Disease	239	295<br>(292 to 298)	-18.9%<br>(-19.7 to -18.1)	1,269	1,384<br>(1,372 to 1,396)	-8.3%<br>(-9.1 to -7.5)	-65,500<br>(-72,200 to -58,800)
Chronic Kidney Disease	283	373<br>(363 to 383)	-24.2%<br>(-26.1 to -22.1)	2,440	1,792<br>(1,766 to 1,818)	36.2%<br>(34.3 to 38.2)	369,900<br>(355,200 to 384,700)
Coeliac Disease	17.8	27.7<br>(27.0 to 28.5)	-35.7%<br>(-37.4 to -34.0)	121	141<br>(139 to 142)	-14.1%<br>(-15.2 to -13.0)	-11,290<br>(-12,320 to -10,270)
COPD	98.8	226<br>(222 to 230)	-56.3%<br>(-57.0 to -55.5)	841	1,030<br>(1,021 to 1,038)	-18.4%<br>(-19.1 to -17.7)	-107,900<br>(-112,800 to -103,000)
Crohn's Disease	15.7	18.9<br>(18.4 to 19.4)	-16.7%<br>(-18.8 to -14.5)	89.1	86.7<br>(85.2 to 88.1)	2.8%<br>(1.1 to 4.5)	1,360<br>(540 to 2,190)
Dementia	156	203<br>(200 to 206)	-22.9%<br>(-24.1 to -21.7)	926	967<br>(959 to 974)	-4.2%<br>(-5.0 to -3.5)	-23,300<br>(-27,700 to -18,900)
Depression	657	930<br>(914 to 947)	-29.4%<br>(-30.6 to -28.1)	3,364	4,647<br>(4,611 to 4,683)	-27.6%<br>(-28.2 to -27.1)	-732,300<br>(-752,900 to -711,600)
Diabetes Mellitus Type 2	367	487<br>(473 to 502)	-24.7%<br>(-26.8 to -22.4)	2,372	2,475<br>(2,443 to 2,506)	-4.2%<br>(-5.4 to -2.9)	-58,720<br>(-76,700 to -40,800)
Epilepsy	38.8	43.3<br>(42.3 to 44.4)	-10.5%<br>(-12.6 to -8.4)	195	205<br>(202 to 207)	-4.8%<br>(-5.9 to -3.6)	-5,570<br>(-6,970 to -4,180)
Heart Failure	211	271<br>(268 to 275)	-22.4%<br>(-23.4 to -21.3)	1,244	1,406<br>(1,395 to 1,416)	-11.5%<br>(-12.2 to -10.8)	-92,400<br>(-98,400 to -86,300)
Multiple Sclerosis	7.3	8.3<br>(8.0 to 8.6)	-12.2%<br>(-15.1 to -9.0)	36.2	40.8<br>(40.1 to 41.5)	-11.1%<br>(-12.6 to -9.6)	-2,580<br>(-2,980 to -2,180)
Osteoporosis	112	175<br>(172 to 178)	-36.0%<br>(-37.0 to -34.9)	727	834<br>(828 to 841)	-12.8%<br>(-13.5 to -12.2)	-61,000<br>(-64,700 to -57,400)
Polymyalgia Rheumatica	46.5	50.6<br>(49.5 to 51.8)	-8.2%<br>(-10.2 to -6.1)	217	230<br>(227 to 232)	-5.7%<br>(-6.7 to -4.7)	-7,470<br>(-8,900 to -6,030)
Psoriasis	69.6	123<br>(121 to 125)	-43.4%<br>(-44.5 to -42.4)	465	559<br>(554 to 564)	-16.8%<br>(-17.5 to -16.0)	-53,400<br>(-56,200 to -50,700)
Rheumatoid Arthritis	31.5	41.3<br>(40.2 to 42.3)	-23.6%<br>(-25.5 to -21.5)	179	195<br>(192 to 197)	-8.2%<br>(-9.3 to -7.0)	-9,100<br>(-10,500 to -7,720)
Stroke/TIA	237	262<br>(260 to 265)	-9.7%<br>(-10.6 to -8.8)	1,215	1,255<br>(1,247 to 1,263)	-3.2%<br>(-3.8 to -2.6)	-22,800<br>(-27,200 to -18,420)
Ulcerative Colitis	25.6	29.1<br>(28.4 to 29.9)	-12.0%<br>(-14.3 to -9.6)	144	136<br>(134 to 138)	6.2%<br>(4.9 to 7.6)	4,810<br>(3,810 to 5,810)

Expected and observed diagnosis rates for each condition are compared during the first year after the onset of the COVID-19 pandemic (March 1, 2020, to February 28, 2021) and during the full study period after the onset of the pandemic (March 1, 2020, to November 30, 2024). Expected diagnosis rates were modelled using Prophet forecasting methodology, utilising data from April, 2016, to February, 2020, under the assumption that pre-pandemic trends would have continued unchanged if the pandemic had not occurred. The relative differences between the number of expected vs. observed diagnoses are shown, in addition to the absolute differences in expected vs. observed diagnoses extrapolated to the full population of England (rounded to the nearest 10 for counts between 1,000 and 10,000; and to the nearest 100 for counts above 10,000). Estimates are shown with 95% prediction intervals (PI). COPD: chronic obstructive pulmonary disease; TIA: transient ischaemic attack.

Supplementary Methods: Autoregressive Integrated Moving Average (ARIMA) modelling

Autoregressive Integrated Moving Average (ARIMA) models were used to analyse time-series of monthly diagnosis rates for the studied long-term conditions, accounting for autocorrelation and seasonality trends within these data. ARIMA (*p,d,q*) models combine an autoregressive component (where *p* is the number of autoregressive terms), a differencing component (where *d* is the order of differencing), and a moving-average component to induce stationarity (where *q* is the order of the moving-average). For time-series with underlying seasonality, Seasonal ARIMA (SARIMA) can be used to account for these trends. SARIMA models are specified as (*p,d,q*)x(*P,D,Q*)*S*, where *P* and *Q* are the autoregressive and moving-average components of the seasonal model, *D* is the seasonal order of differencing, and *S* is the seasonality in months.

To identify optimal SARIMA terms for each long-term condition, we first used the *auto.arima()* function within the *Forecast* package in R, based upon the approaches outlined by Schaffer *et al.* and Qi *et al.*[1-3] Initial model selection was performed by applying iterative, non-stepwise selection of differencing, seasonality, autoregressive, and moving-average terms to minimise the AIC (Akaike Information Criterion) and BIC (Bayesian Information Criterion), using age and sex-standardised diagnosis rates per 100,000 population from April 2016 to February 2020 (i.e. prior to the onset of the COVID-19 pandemic). Serial Kwiatkowski–Phillips–Schmidt–Shin (KPSS) tests were used to test for stationarity of the time series, with differencing applied to non-stationary time series where appropriate. Model verification was then performed, with residual diagnostics evaluated by comparing plots of residuals over time, residual histograms, residual autocorrelation and partial autocorrelation plots, in addition to Ljung–Box tests at 12 and 24 lags to assess white-noise behaviour and for remaining autocorrelation. Model accuracy was assessed using the root mean squared error (RMSE), expressed both in absolute terms and as a percentage of the series mean (normalised RMSE) to facilitate interpretability across scales. Bai-Perron tests were used to detect structural breaks in pre-pandemic time series, with break selection based upon the BIC. Cumulative Sum (CUSUM) tests were used to assess parameter stability over time. For model terms where there were violations of assumptions and/or poor fit on visual inspection, alternative SARIMA terms were explored iteratively, with model fit, diagnostics and visual inspection then re-evaluated. The SARIMA model specifications utilised for each long-term condition are listed below.

	**SARIMA terms (p, d, q) (P, D, Q)[S]**	**AIC**	**BIC**	**Sigma^2 estimate**	**Log likelihood**
Asthma	(0,0,0)(0,1,1)[12]	146.7	149.8	3.1	-71.3
Atopic Dermatitis	(0,1,1)(0,1,1)[12]	76.6	81.2	0.47	-35.3
Coronary Heart Disease	(1,0,0)(0,1,1)[12] with drift	94.8	101.1	0.62	-43.4
Chronic Kidney Disease	(0,0,1)(0,1,2)[12]	182.6	188.8	6.3	-87.3
Coeliac Disease	(0,0,0)(1,1,1)[12] with drift	-6.5	-0.28	0.04	7.3
COPD	(0,0,0)(0,1,1)[12] with drift	109.2	113.9	1.05	-51.6
Crohn's Disease	(0,0,0)(0,1,1)[12] with drift	-26.2	-21.5	0.02	16.1
Dementia	(3,0,0)(0,1,1)[12]	99.8	107.5	0.76	-44.9
Depression	(0,0,0)(0,1,1)[12] with drift	209.8	214.5	16.5	-101.9
Depression (sensitivity)	(0,0,0)(0,1,1)[12] with drift	227.1	231.8	29.4	-110.5
Diabetes Mellitus Type 2	(4,0,1)(0,1,1)[12] with drift	186.2	198.6	6.0	-85.1
Epilepsy	(0,0,0)(1,1,1)[12]	11.0	15.7	0.04	-2.5
Heart Failure	(3,0,0)(0,1,1)[12] with drift	102.5	111.8	0.56	-45.3
Multiple Sclerosis	(4,0,0)(1,1,0)[12]	-78.6	-69.3	0.004	45.3
Osteoporosis	(0,0,0)(0,1,1)[12]	89.2	92.3	0.62	-42.6
Polymyalgia Rheumatica	(0,0,1)(1,1,0)[12] with drift	23.4	29.6	0.10	-7.7
Psoriasis	(0,0,0)(0,1,1)[12] with drift	68.6	73.3	0.31	-31.3
Rheumatoid Arthritis	(0,0,0)(0,1,1)[12]	17.2	20.4	0.07	-6.6
Stroke/TIA	(0,0,2)(1,1,0)[12]	80.9	87.1	0.47	-36.4
Ulcerative Colitis	(0,0,0)(0,1,1)[12]	-4.1	-0.98	0.04	4.0

All monthly forecasted estimates from SARIMA models were presented with 95% prediction intervals, obtained via residual bootstrapping with 10,000 replications as implemented in the *Forecast* package in R. Differences between observed and expected diagnosis rates were reported for each long-term condition over the following time-periods: full period after the onset of the pandemic (March, 2020, to November, 2024); first year of the pandemic (March, 2020, to February, 2021); second year (March, 2021, to February, 2022); third year (March, 2022, to February, 2023); and from March, 2023, to November, 2024. Differences in new diagnoses with relation to the full population of England were estimated by multiplying forecasted rates for absolute differences in observed vs. expected diagnosis rates (mean, lower and upper bounds) by the mid-year population estimates for England (obtained from the Office for National Statistics).[4] 95% prediction intervals for periods including multiple months were calculated by: (1) converting monthly bounds obtained from the *Forecast* package to standard errors, as SE = (upper - lower) / (2 * 1.96); (2) summing predicted rates and adding variances; and (3) constructing 95% prediction intervals as mean +/- 1.96 * sqrt(total variance).

To assess the robustness of SARIMA model specification, we conducted a sensitivity analysis by modelling the monthly time-series using *Prophet*.[5] *Prophet* specifies a decomposable structure with a piecewise-linear trend and seasonal components represented by Fourier terms. In these models, yearly seasonality was modelled additively, and forecasts were generated with 95% prediction intervals obtained from *Prophet*'s simulation-based procedure. *Prophet* forecasts and intervals were compared with those from SARIMA to assess the robustness of conclusions to model choice.

Supplementary Data: Diagnostic codelists

Incident diagnoses were defined as the first appearance of a diagnostic code for a condition in the primary care or hospitalisation record of individuals from the reference population who did not previously have recorded diagnostic codes for that specific condition.

Primary care diagnoses were defined using the NHS England Primary Care Domain Reference Set codelists, which are collections of SNOMED codes published by NHS England and updated on a regular basis.[6] These reference sets can be used by clinicians, policy makers, and researchers to collate information on individuals with recorded diagnoses of a particular condition – for example, for use alongside the Quality and Outcomes Framework (QOF) business rules – thereby facilitating comparisons between practices and studies over time.

A comparable set of reference codelists do not exist for hospitalisations, which are based upon the ICD-10 coding system. As such, clinicians within the author team generated a series of ICD-10 codelists for each studied condition, based upon clinical expertise and with reference to other published codelists (for example, OpenCodelists, and the London School for Hygiene and Tropical Medicine Data Compass). Hospitalisations where relevant diagnostic codes were listed as the primary cause for that admission were included in estimates of incident and prevalent diagnoses; however, hospitalisations where diagnostic codes were listed in secondary positions were not included, due to less reliable coding. For example, the inclusion of secondary admission codes for rheumatoid arthritis resulted in incidence rates that were 3-fold higher than estimates obtained from previous population-level studies.[7,8]

Prevalent diagnoses were defined as individuals from the reference population who had prevalent diagnostic codes for a condition in either their primary care or hospitalisation record. For several conditions (defined below), SNOMED codes representing resolved diagnoses of a particular condition also exist – i.e. suggesting the condition is no longer present. Individuals who had resolved codes for a diagnosis that was not superseded by another non-resolved diagnostic code within the study period were not classed as prevalent diagnoses.

Asthma

Primary care codelists (SNOMED)

https://www.opencodelists.org/codelist/nhsd-primary-care-domain-refsets/ast_cod/3a005293

https://www.opencodelists.org/codelist/nhsd-primary-care-domain-refsets/astadmsn_cod/3f5c6982

Secondary care codelist (ICD-10)

https://www.opencodelists.org/codelist/user/markdrussell/asthma-secondary-care/2a250f1b

Resolved codelist (SNOMED)

https://www.opencodelists.org/codelist/nhsd-primary-care-domain-refsets/astres_cod/530fc5c8

Atopic dermatitis

Primary care codelists (SNOMED)

https://www.opencodelists.org/codelist/nhsd-primary-care-domain-refsets/atopic-dermatitis-codes/5816a06b

Secondary care codelist (ICD-10)

https://www.opencodelists.org/codelist/user/markdrussell/atopic-dermatitis-secondary-care/645c3567

Resolved codelist not available

Coronary heart disease

Primary care codelists (SNOMED)

https://www.opencodelists.org/codelist/nhsd-primary-care-domain-refsets/chd_cod/0f32f87c

Secondary care codelist (ICD-10)

https://www.opencodelists.org/codelist/user/markdrussell/coronary-heart-disease-secondary-care/11159be6

Resolved codelist not available

Chronic kidney disease

Primary care codelists (SNOMED)

https://www.opencodelists.org/codelist/nhsd-primary-care-domain-refsets/ckdatrisk2_cod/4529f7b4

Secondary care codelist (ICD-10)

https://www.opencodelists.org/codelist/user/markdrussell/chronic-kidney-disease-secondary-care/167b1bd2

Resolved codelist (SNOMED)

https://www.opencodelists.org/codelist/nhsd-primary-care-domain-refsets/ckdres_cod/305aab93

Coeliac disease

Primary care codelists (SNOMED)

https://www.opencodelists.org/codelist/nhsd-primary-care-domain-refsets/coeliac-disease-codes/22e654c2

Secondary care codelist (ICD-10)

https://www.opencodelists.org/codelist/user/markdrussell/coeliac-secondary-care/001e6893

Resolved codelist not available

Chronic obstructive pulmonary disease

Primary care codelists (SNOMED)

https://www.opencodelists.org/codelist/nhsd-primary-care-domain-refsets/copd_cod/3a5ef7dc

https://www.opencodelists.org/codelist/nhsd-primary-care-domain-refsets/copdadmsn_cod/35d15b44

Secondary care codelist (ICD-10)

https://www.opencodelists.org/codelist/user/markdrussell/COPD_admission/43348250

Resolved codelist (SNOMED)

https://www.opencodelists.org/codelist/nhsd-primary-care-domain-refsets/copdres_cod/67806ca0

Crohn's disease

Primary care codelists (SNOMED)

https://www.opencodelists.org/codelist/nhsd-primary-care-domain-refsets/crohns-disease-codes/3f6f3b7c

Secondary care codelist (ICD-10)

https://www.opencodelists.org/codelist/user/markdrussell/crohns-disease-secondary-care/7ab8e8a6

Resolved codelist not available

Dementia

Primary care codelists (SNOMED)

https://www.opencodelists.org/codelist/nhsd-primary-care-domain-refsets/dem_cod/21b792ed

Secondary care codelist (ICD-10)

https://www.opencodelists.org/codelist/user/markdrussell/dementia-secondary-care/45e74246

Resolved codelist not available

Depression

Primary care codelists (SNOMED)

https://www.opencodelists.org/codelist/nhsd-primary-care-domain-refsets/depr_cod/477e1261

https://www.opencodelists.org/codelist/nhsd-primary-care-domain-refsets/depsupp_cod/74fe2c6e

Secondary care codelist (ICD-10)

https://www.opencodelists.org/codelist/user/markdrussell/depression-secondary-care/192ddbc8

Resolved codelist (SNOMED)

https://www.opencodelists.org/codelist/nhsd-primary-care-domain-refsets/depres_cod/1369b693

Depression (broader definition, including symptomatic codes)

Primary care codelists (SNOMED)

https://www.opencodelists.org/codelist/user/markdrussell/depression_broad/68217c3b/

Secondary care codelist (ICD-10)

https://www.opencodelists.org/codelist/user/markdrussell/depression-secondary-care/192ddbc8

Resolved codelist (SNOMED)

https://www.opencodelists.org/codelist/nhsd-primary-care-domain-refsets/depres_cod/1369b693

Diabetes mellitus (type 2)

Primary care codelists (SNOMED)

https://www.opencodelists.org/codelist/nhsd-primary-care-domain-refsets/dmtype2audit_cod/130b114a

Secondary care codelist (ICD-10)

https://www.opencodelists.org/codelist/user/markdrussell/type-2-diabetes-secondary-care/2f8a8f07

Resolved codelist (SNOMED)

https://www.opencodelists.org/codelist/nhsd-primary-care-domain-refsets/dmres_cod/62d83746

Epilepsy

Primary care codelists (SNOMED)

https://www.opencodelists.org/codelist/nhsd-primary-care-domain-refsets/epil_cod/28ffb643

Secondary care codelist (ICD-10)

https://www.opencodelists.org/codelist/user/markdrussell/epilepsy-secondary-care/13c85bdc

Resolved codelist (SNOMED)

https://www.opencodelists.org/codelist/nhsd-primary-care-domain-refsets/epilres_cod/26b50493

Heart failure

Primary care codelists (SNOMED)

https://www.opencodelists.org/codelist/nhsd-primary-care-domain-refsets/hf_cod/70b49c5d

https://www.opencodelists.org/codelist/nhsd-primary-care-domain-refsets/hflvsd_cod/44634425

Secondary care codelist (ICD-10)

https://www.opencodelists.org/codelist/user/markdrussell/heart-failure-secondary-care/7822ef0e

Resolved codelist (SNOMED)

https://www.opencodelists.org/codelist/nhsd-primary-care-domain-refsets/hfres_cod/627991fd

Multiple sclerosis

Primary care codelists (SNOMED)

https://www.opencodelists.org/codelist/nhsd-primary-care-domain-refsets/multiple-sclerosis-codes/5ac96061

Secondary care codelist (ICD-10)

https://www.opencodelists.org/codelist/user/markdrussell/multiple-sclerosis-secondary-care/2cd7cf11

Resolved codelist not available

Osteoporosis

Primary care codelists (SNOMED)

https://www.opencodelists.org/codelist/nhsd-primary-care-domain-refsets/osteo_cod/1dd61122

Secondary care codelist (ICD-10)

https://www.opencodelists.org/codelist/user/markdrussell/osteoporosis-secondary-care/4b4cc232

Resolved codelist (SNOMED)

https://www.opencodelists.org/codelist/user/markdrussell/osteoporosis-resolved/02d12889

Polymyalgia rheumatica

Primary care codelists (SNOMED)

https://www.opencodelists.org/codelist/nhsd-primary-care-domain-refsets/polymyalgia-rheumatica-pmr-codes/5563e075

Secondary care codelist (ICD-10)

https://www.opencodelists.org/codelist/user/markdrussell/polymyalgia-rheumatica-pmr-secondary-care/670ef55d

Resolved codelist not available

Psoriasis

Primary care codelists (SNOMED)

https://www.opencodelists.org/codelist/nhsd-primary-care-domain-refsets/adult-and-child-psoriasis-codes/325020f7

Secondary care codelist (ICD-10)

https://www.opencodelists.org/codelist/user/markdrussell/psoriasis-secondary-care/7d6ba89c

Resolved codelist not available

Rheumatoid arthritis

Primary care codelists (SNOMED)

https://www.opencodelists.org/codelist/nhsd-primary-care-domain-refsets/rheumatoid-arthritis-disorders/04d006ea

Secondary care codelist (ICD-10)

https://www.opencodelists.org/codelist/user/markdrussell/rheumatoid-arthritis-secondary-care/245780e9

Resolved codelist not available

Stroke/transient ischaemic attack

Primary care codelists (SNOMED)

https://www.opencodelists.org/codelist/nhsd-primary-care-domain-refsets/strk_cod/78779ff3

https://www.opencodelists.org/codelist/nhsd-primary-care-domain-refsets/tia_cod/1625df86

Secondary care codelist (ICD-10)

https://www.opencodelists.org/codelist/user/markdrussell/stroke-and-tia-secondary-care/780628b0

Resolved codelist not available

Ulcerative colitis

Primary care codelists (SNOMED)

https://www.opencodelists.org/codelist/nhsd-primary-care-domain-refsets/ulcerative-colitis-uc-codes/518bc7a7

Secondary care codelist (ICD-10)

https://www.opencodelists.org/codelist/user/markdrussell/ulcerative-colitis-secondary-care/61a97571

Resolved codelist not available

Ethnicity codelist

Primary care codelists (SNOMED)

https://www.opencodelists.org/codelist/opensafely/ethnicity-snomed-0removed/22911876

Where ethnicity was not recorded in an individual's primary care record, ethnicity data were extracted from the individual's secondary care (SUS) record, where available.

References

1. Schaffer AL, Dobbins TA, Pearson SA. Interrupted time series analysis using autoregressive integrated moving average (ARIMA) models: a guide for evaluating large-scale health interventions. *BMC Med Res Methodol* 2021; **21**(1): 58.
2. Qi C, Osborne T, Bailey R, et al. Impact of COVID-19 pandemic on incidence of long-term conditions in Wales: a population data linkage study using primary and secondary care health records. *British Journal of General Practice* 2023; **73**(730): e332-e9.
3. Hyndman RJ, Khandakar Y. Automatic Time Series Forecasting: The forecast Package for R. *Journal of Statistical Software* 2008; **27**(3): 1 - 22.
4. Office for National Statistics. Estimates of the population for the UK, England and Wales, Scotland and Northern Ireland. 2024. https://www.ons.gov.uk/peoplepopulationandcommunity/populationandmigration/populationestimates/datasets/populationestimatesforukenglandandwalesscotlandandnorthernireland (accessed 13/09/2025.
5. Taylor S, Letham B. Prophet: Automatic Forecasting Procedure. 2021. https://CRAN.R-project.org/package=prophet. (accessed 13/09/2025.
6. NHS England. Primary Care Domain Reference Set Portal. 2025. https://digital.nhs.uk/data-and-information/data-collections-and-data-sets/data-collections/quality-and-outcomes-framework-qof/quality-and-outcome-framework-qof-business-rules/primary-care-domain-reference-set-portal (accessed 13/09/2025.
7. Scott IC, Whittle R, Bailey J, et al. Rheumatoid arthritis, psoriatic arthritis, and axial spondyloarthritis epidemiology in England from 2004 to 2020: An observational study using primary care electronic health record data. *The Lancet Regional Health – Europe* 2022; **23**.
8. Russell MD, Galloway JB, Andrews CD, et al. Incidence and management of inflammatory arthritis in England before and during the COVID-19 pandemic: a population-level cohort study using OpenSAFELY. *The Lancet Rheumatology* 2022; **4**(12): e853-e63.
